# Misidentification of runs of homozygosity islands in cattle caused by interference with copy number variation or large intermarker distances

**DOI:** 10.1186/s12711-018-0414-x

**Published:** 2018-08-22

**Authors:** Wilson Nandolo, Yuri T. Utsunomiya, Gábor Mészáros, Maria Wurzinger, Negar Khayadzadeh, Rafaela B. P. Torrecilha, Henry A. Mulindwa, Timothy N. Gondwe, Patrik Waldmann, Maja Ferenčaković, José F. Garcia, Benjamin D. Rosen, Derek Bickhart, Curt P. van Tassell, Ino Curik, Johann Sölkner

**Affiliations:** 1 Division of Livestock Sciences (NUWI), University of Natural Resources and Life Sciences, Gregor-Mendel Strasse 33, 1180 Vienna, Austria; 2 Lilongwe University of Agriculture and Natural Resources, P. O. Box 219, Lilongwe, Malawi; 3 School of Agricultural and Veterinarian Sciences, Jaboticabal, Department of Preventive Veterinary Medicine and Animal Reproduction, São Paulo State University (UNESP), São Paulo, Brazil; 4 National Livestock Resources Research Institute, P.O Box 96, Tororo, Uganda; 5 Department of Animal Breeding and Genetics, Swedish University of Agricultural Sciences, Box 7023, 750 07 Uppsala, Sweden; 6 Department of Animal Science, Faculty of Agriculture, University of Zagreb, Svetošimunska cesta 25, 10000 Zagreb, Croatia; 7 School of Veterinary Medicine, Araçatuba, Department of Support, Production and Animal Health, São Paulo State University (UNESP), São Paulo, Brazil; 8 Animal Genomics and Improvement Laboratory, Beltsville, MD 20705-2350 USA

## Abstract

**Background:**

Runs of homozygosity (ROH) islands are stretches of homozygous sequence in the genome of a large proportion of individuals in a population. Algorithms for the detection of ROH depend on the similarity of haplotypes. Coverage gaps and copy number variants (CNV) may result in incorrect identification of such similarity, leading to the detection of ROH islands where none exists. Misidentified hemizygous regions will also appear as homozygous based on sequence variation alone. Our aim was to identify ROH islands influenced by marker coverage gaps or CNV, using Illumina BovineHD BeadChip (777 K) single nucleotide polymorphism (SNP) data for Austrian Brown Swiss, Tyrol Grey and Pinzgauer cattle.

**Methods:**

ROH were detected using clustering, and ROH islands were determined from population inbreeding levels for each marker. CNV were detected using a multivariate copy number analysis method and a hidden Markov model. SNP coverage gaps were defined as genomic regions with intermarker distances on average longer than 9.24 kb. ROH islands that overlapped CNV regions (CNVR) or SNP coverage gaps were considered as potential artefacts. Permutation tests were used to determine if overlaps between CNVR with copy losses and ROH islands were due to chance. Diversity of the haplotypes in the ROH islands was assessed by haplotype analyses.

**Results:**

In Brown Swiss, Tyrol Grey and Pinzgauer, we identified 13, 22, and 24 ROH islands covering 26.6, 389.0 and 35.8 Mb, respectively, and we detected 30, 50 and 71 CNVR derived from CNV by using both algorithms, respectively. Overlaps between ROH islands, CNVR or coverage gaps occurred for 7, 14 and 16 ROH islands, respectively. About 37, 44 and 52% of the ROH islands coverage in Brown Swiss, Tyrol Grey and Pinzgauer, respectively, were affected by copy loss. Intersections between ROH islands and CNVR were small, but significantly larger compared to ROH islands at random locations across the genome, implying an association between ROH islands and CNVR. Haplotype diversity for reliable ROH islands was lower than for ROH islands that intersected with copy loss CNVR.

**Conclusions:**

Our findings show that a significant proportion of the ROH islands in the bovine genome are artefacts due to CNV or SNP coverage gaps.

**Electronic supplementary material:**

The online version of this article (10.1186/s12711-018-0414-x) contains supplementary material, which is available to authorized users.

## Background

A run of homozygosity (ROH) refers to a continuous stretch of homozygous loci in the genome [1]. ROH are typically detected based on the genotypes at single nucleotide polymorphisms (SNPs) that are derived either from high-throughput microarrays or next-generation sequencing data [2]. ROH can either appear by chance or simply be artefacts caused by imperfect SNP coverage in the design of a chip. The proportion of an individual’s genome that is located within ROH is an approximate measure of inbreeding [3], where longer ROH most likely derive from more recent common ancestors [4]. Estimates of ROH inbreeding coefficients have been shown to be more accurate pedigree inbreeding coefficients [2, 5].

Studies on ROH in some European cattle breeds have revealed the presence of distinct genomic regions with ROH that are common between individuals, within a breed and even across breeds. These common ROH are called ROH hotspots or ROH islands [3] and in this paper, we use the term “ROH islands”. Zavarez et al. [6] found three ROH islands on chromosomes 4, 7 and 12 and four ROH islands on the X chromosome in Nellore cattle. Karimi [7] identified ROH islands on chromosomes 7 and 21 in *Bos indicus*, and on *Bos taurus* (BTA) chromosomes 5, 6, 7, 16 and 21. Particularly notable, ROH islands present in a large proportion of the individuals of the population have been identified on BTA6 in the Brown Swiss, Pinzgauer and Tyrol Grey bovine breeds [8]. The distribution and pattern of ROH islands can indicate a pattern of selection events and this is of interest for any breeding program. Thus, it is important to know the location and distribution of the ROH islands for a given population.

The reasons why ROH islands occur are not well understood. Theoretically, ROH islands within a breed may be explained by shared recent ancestry [9]. Szmatoła et al. [10] hypothesized that such ROH islands may be due to selection at functionally important quantitative trait loci, which would imply high local linkage disequilibrium (LD) in those genomic regions. In humans, Nothnagel et al. [11] noted that regional LD between SNPs is not sufficient to explain the occurrence of ROH islands.

False ROH can be detected if the maximum gap allowed between homozygous SNPs is too large. This applies especially to short runs and in the case of low-density SNP chips, as indicated by Ferenčaković et al. [12]. Other reasons for detecting false ROH are reference genome assembly problems, the occurrence of rare alleles in the reference genome and local ascertainment bias resulting from sampling of the SNPs that are included in the SNP panel. ROH islands may also be due to biological factors, such as differences in chromosome structure that are perceived as stretches of homozygous genotypes by the SNP assays. For instance, it is hypothesized that ROH islands may be related to the centromeric location of the ROH [2], although in cattle this would hold only if the ROH island is found within the first few Mb of a chromosome, since all bovine chromosomes are acrocentric. ROH islands may also result from the existence of structural variants (SV). SV are genomic rearrangements that affect more than 50 base pairs (bp) of sequence and can be due to deletions, insertions, inversions, transpositions, duplications and translocations [13]. Typical SV are copy number variants (CNV), defined as DNA segments of one kilobase (kb) or more that are present in variable copy number in comparison with a reference genome [14].

**Fig. 1:**
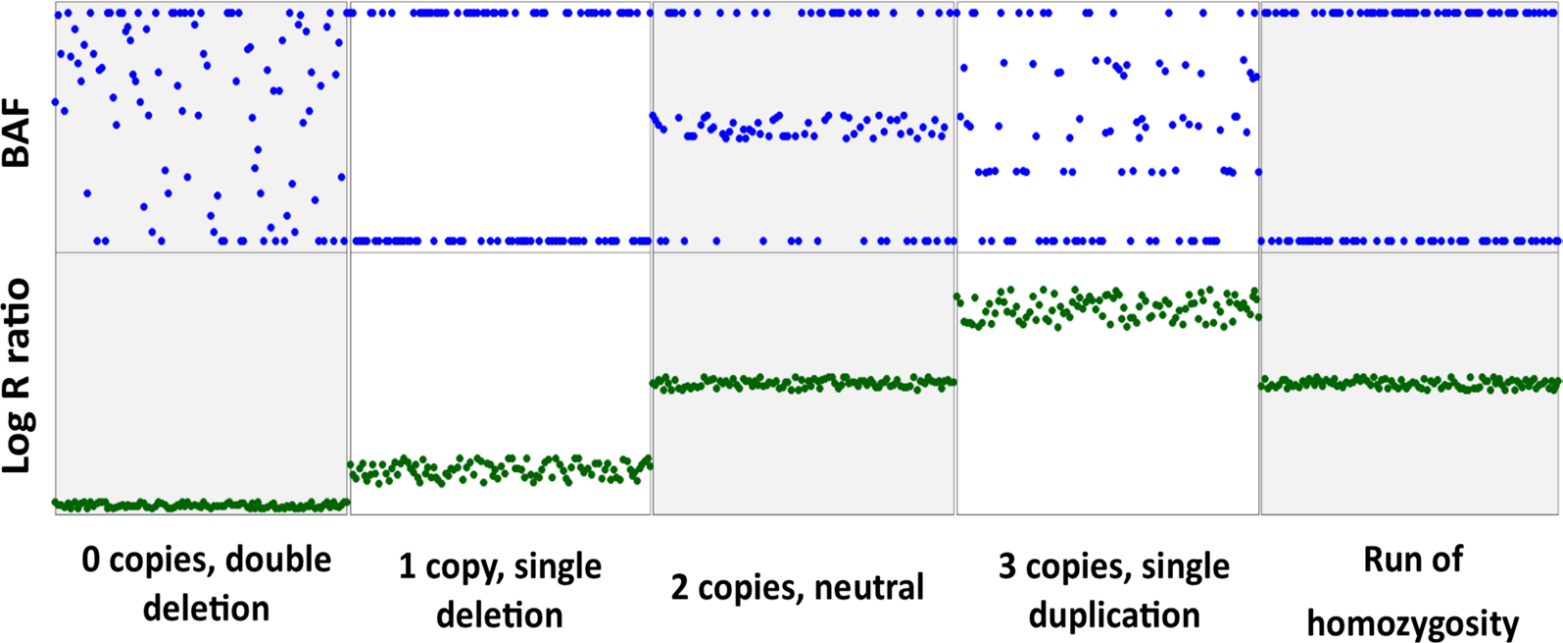
BAF and LRR plots for scenarios with different copy numbers and homozygous alleles. The plots show how some regions that contain CNV can be erroneously determined as having ROH. The normal state is to carry two copies of an allele, and the BAF values are distributed between intermediate and extreme values, while the LRR ratio has intermediate values. As the number of copies increases, the LRR values move towards the higher extreme while the BAF values disperse into more intermediate values based on copy number. When the copy number is very large, this segment may also be mistyped as being homozygous and ROH algorithms may detect it as a ROH. A segment, which is truly homozygous has extreme BAF values and intermediate LRR values

This paper explores the possibility that ROH islands are artefacts resulting from limitations of the algorithms that are used to detect ROH with Illumina assay SNP chip data. The GenTrain algorithm used by Illumina assays depends on the intensity of the signals emitted by a probe at a specific marker compared to expected intensity (log R ratio, LRR), and the proportion of hybridized sample that carries the B allele as designated by the hybridization assay (B-allele frequency, BAF), usually normalized to 0.0, 0.5, and 1.0 [15]. As illustrated in Fig. 1, a genome segment with a double deletion has random BAF values, and very low LRR values (implying a low signal intensity) [13]. A segment with a copy loss has lower LRR values and its BAF values tend towards the extremes (very high and very low). The GenTrain algorithm may mistype this hemizygotic region as being homozygous and ROH algorithms may detect this as a ROH. Thus, taking CNV into account is very important to eliminate erroneously detected ROH.

Thus, one of the first steps towards understanding why ROH islands exist is to distinguish true ROH from ROH artefacts. The objective of this paper was to identify artefactual ROH islands resulting from large intermarker distances (IMD) and/or interference with copy number variation (CNV) that were detected by two methodologies.

## Methods

### Data and data preparation

We used Illumina BovineHD BeadChip (777 K) genotype data from three Austrian cattle breeds. The dataset comprised 48 Brown Swiss, 120 Tyrol Grey and 119 Pinzgauer bulls. Quality control of the genotype data was done using PLINK [16] with the following parameters: call rate higher than 0.9, departure from Hardy–Weinberg equilibrium at the 0.001 level, missing genotype rate less than 0.05 and missing data rate less than 0.1. The numbers of animals that passed quality control for ROH analysis were 46, 117 and 118 for Brown Swiss, Tyrol Grey and Pinzgauer, respectively.

### ROH analysis

ROH were detected using a clustering algorithm implemented in the SNP & Variation Suite (SVS) based on the paper of Zhang et al. [17]. ROH were detected using ROH lengths of more than 1, 2, 4, 8 and 16 Mb, each. Different numbers of heterozygotes and missing SNPs were allowed for each of the ROH length categories (1, 2, 4, 8, 16 for heterozygotes and 4, 8, 16, 32, 64 for missing SNPs for 1 to 2, 2 to 4, 4 to 8, 8 to 16, > 16 Mb, respectively) as recommended by Ferenčaković et al. [12]. ROH for each individual were merged using the BEDTools software [18].

### Detection of CNV

CNV were detected by using two algorithms: the multivariate copy number analysis method implemented in the Golden Helix SVS v8.5 software (Golden Helix, Inc., Bozeman, MT, www.goldenhelix.com) and a hidden Markov model (HMM) implemented in PennCNV [19].

#### Detection of CNV with the SNP & Variation Suite

LRR and BAF data were extracted from the Illumina BovineHD final reports and imported into SVS. Only markers with GC scores higher than 0.7 were used. Wave detection and correction on autosomes were based on the University of Maryland assembly of *Bos taurus*, release 3.1 (UMD3.1, bosTau6) with a minimum training marker distance of 1000 kb. Using the recommended wave factor threshold of 0.05 [20], the numbers of animals that passed this step were equal to 40, 64 and 89 for Brown Swiss, Tyrol Grey and Pinzgauer, respectively. Means of CNV segments were computed using the SVS multivariate copy number analysis method (CNAM) and the optimal segmenting procedure. The maximum number of segments allowed per 10,000 markers was 20; the minimum number of markers per segment was 3; and the maximum pairwise segment *P* value was 0.005 (with 2000 permutations per pair). The copy number segment list was discretized using a three-state model (− 1, 0,1) based on a segmentation mean threshold of 0.3 as used by Zhou et al. [21]. In the three-state model, “− 1” denotes a copy loss, “0” denotes copy neutral and “1” denotes copy gain.

#### Detection of CNV in PennCNV

BAF and LRR ratio data were extracted into individual raw files from the Illumina final reports using the “split_illumina_report.pl” script. These data were used to generate files of population frequencies of the B allele (PFB) for each breed using “compile_pfb.pl” script. A GC content file for bosTau6 was downloaded from http://hgdownload.cse.ucsc.edu/gbdb/bosTau6/bbi/gc5Base.bw. The file was converted to the appropriate wiggle track format using BigWig and BigBed Tools [22]. Appropriate “gcmodel” files were generated for each breed using the “cal_gc_snp.pl” script based on a 1000-kb span (500 kb on each side of a marker). The CNV calling procedure was run with the “gcmodel” option for detection and adjustment of the intensity values [19] using the “detect_cnv.pl” script. Post-processing of the CNV calls was done using the “filter_cnv.pl” script with the same quality control parameters as those used in SVS: absolute wave factor value (0.05), minimum number of markers per segment (3), and LRR standard deviation (0.3). In addition, a BAF drift threshold of 0.01 was used. Using the 1.5× inter-quartile range rule [23], the distribution of the number of CNV calls per sample was used to re-run the filter procedure to exclude samples with CNV calls greater than 59, 120 and 114 in Brown Swiss, Tyrol Grey and Pinzgauer, respectively, as done by Ghani et al. [24]. The numbers of animals that remained at this point were 41, 55 and 98 for Brown Swiss, Tyrol Grey and Pinzgauer, respectively. Finally, CNV calls with gaps shorter than 20% of their combined CNV lengths were merged using the “clean_cnv.pl” script.

#### Sample sizes after quality control

To obtain consensus results between the two CNV calling algorithms used, only the animals, which passed quality control in both analyses, were considered to determine the proportion of CNV in regions of ROH islands. The final numbers of animals were 37, 52 and 87 for Brown Swiss, Tyrol Grey and Pinzgauer, respectively.

### Computation of CNV regions

CNV that overlapped by at least 1 bp were merged using BEDTools [18] as done by Prinsen et al. [20]. CNV regions (CNVR) were divided into three categories: gain, loss or both (for regions with copy gains, copy losses and both copy gains and losses for different samples), respectively. A consensus list of CNVR (CNVR derived from CNV detected by both SVS and PennCNV) was generated using the “intersect” procedure of BEDTools [18], while the overlaps between the CNVR in this study and other studies were determined using the “merge” procedure of the same software.

### Determination of ROH islands

ROH detected in the genome of the animals in the final sample were used to determine ROH islands. Inbreeding levels of the markers were computed by calculating the proportion of individuals for which the marker was homozygous. ROH islands were defined as regions where the inbreeding level for markers passed the 99th percentile of the genome-wide distribution of inbreeding levels.

### Determination of possibly invalid ROH islands

The following metrics were computed from the CNV and ROH analyses:

- Proportion of individuals inbred at each marker.
- Proportions of individuals with CNV at each marker by category of copy state (gain, loss or both).
- Mean IMD within the ROH island; using Tukey’s box-plot method for identifying outliers [23], the upper limit for defining outliers was 9.2365 kb.

ROH islands that overlapped with CNVR and that had a mean IMD longer than 9.2365 kb were considered as likely artefacts.

### Testing for significance of overlaps between ROH islands and copy number variant regions

A permutation test was performed to check whether the overlaps between ROH and CNVR were due to chance. The positions of the ROH islands for each breed for each algorithm used to detect CNV were randomized 10,000 times with the constraint that ROH islands on the same chromosome should be more than 1 Mb apart. The intersection of the CNVR and the ROH islands based on the test data (ROHD) were compared with the intersections between the CNVR and the randomized ROH islands (ROHR) using a t-test.

### Haplotype diversity within ROH islands and intersections of ROH islands and CNVR with copy losses

Genotyping data of the animals that passed quality control were phased using the genetic model of coalescence with recombination implemented in the SHAPEIT software [25] with default options. Each ROH island was assigned to one of seven categories based on whether it presented a coverage gap and/or a copy loss and/or a copy gain or none of these issues. Each ROH island was split into 100-kb blocks using the “ghap.blockgen” function in GHap [26], which is a package in R [27]. The average size of bovine haplotype blocks ranges from 10 to 20 kb but can be as long as 700 kb [28, 29]. The 100-kb size was selected as a conservative size that is within the range of reported maximum haplotype block sizes. Effective numbers of haplotypes for each haplotype block were computed as the inverse of the sum of the squares of the frequencies of haplotypes within a block. Expected block heterozygosity ($H$) was used as a measure of haplotype diversity, and was computed as:

$$H = 1 - \varSigma p^{2} ,$$

where $p$ is the frequency of each haplotype in the block. Haplotype diversity and (effective) number of haplotypes were compared between breeds and different categories of ROH islands: ROH islands with no coverage gap; ROH islands with a coverage gap, and ROH islands with a copy loss (copy loss only or copy loss with copy gain and/or coverage gap).

## Results and discussion

### Distribution, sizes and coverage of ROH islands

The genomes of each of the 176 animals in the dataset of interest had ROH. The mean inbreeding coefficients based on the overall sum of ROH (F_ROH_) with a minimum length of 1 Mb were 13.3, 5.8 and 5.6% in Brown Swiss, Tyrol Grey and Pinzgauer, respectively (see Additional file 1: Table S1). The largest sum of ROH was 588 Mb (F_ROH_ = 20%) in a Tyrol Grey individual, while the smallest was 45 Mb (F_ROH_ = 2%) in a Pinzgauer individual. In the three breeds, 59 ROH islands were identified based on 99th percentile marker inbreeding level cut-off points of 45.95, 19.23 and 17.24%, respectively, of which only 44 were unique (see Additional file 1: Table S2). The ROH islands are shown in the Manhattan plots in Figure S1 (see Additional file 2: Figure S1) and their descriptive statistics are in Table 1. Two ROH islands were common to Brown Swiss and Pinzgauer; three were common to Tyrol Grey and Pinzgauer; and five were common to all three breeds, two on BTA6 and one each on BTA7, 10 and 12. Overall, BTA6 had the largest number of ROH islands (five in Brown Swiss, six in Pinzgauer and two in Tyrol Grey), which is consistent with the results of Ferenčaković et al. [8] and Karimi et al. [7] in other *B. taurus* breeds. There were no significant differences in the size and genome coverage of ROH islands.

**Table 1:** Size of ROH islands in Brown Swiss, Tyrol Grey and Pinzgauer cattle

Breed	Number of autosomes with ROH islands	Number of ROH islands	ROH island length (bp)				Coverage (Mb)
			Min	Median	Mean	Max	
Brown Swiss	8	13	34,863	1,662,891	2,049,006	6,624,458	26.637
Tyrol Grey	17	22	16,770	1,397,826	1,771,718	5,948,811	38.978
Pinzgauer	14	24	5309	1,194,240	1,493,608	4,651,919	35.847

### Distribution, size and coverage of CNVR

In total, 306 (187), 606 (153) and 528 (178) CNVR were identified in Brown Swiss, Tyrol Grey and Pinzgauer, respectively, using PennCNV and (SVS). Thirty, 50 and 71 consensus CNVR were found in Brown Swiss, Tyrol Grey and Pinzgauer, respectively. A full list of the CNVR is in Table S3 (see Additional file 1: Table S3), and a summary is in Table 2. PennCNV detected more CNV with copy gain than SVS. Overall, most of the CNVR had copy losses. Based on PennCNV’s CNV calls, the largest number of CNVR was identified on BTA19 (37, 62 and 47 for Brown Swiss, Tyrol Grey and Pinzgauer, respectively) and, the smallest number (0, 1 and 0, respectively) on BTA27. The average number of CNVR per chromosome was equal to 10.55, 21.14 and 18.21 for Brown Swiss, Tyrol Grey and Pinzgauer, respectively. Based on SVS’s CNV calls, the largest number of CNVR (given in parentheses) were identified on BTA8 (17), 12 (12) and 2 (13), in Brown Swiss, Tyrol Grey and Pinzgauer, respectively, and the smallest number of CNVR were on BTA25 (0), 26 and 14 (0 each) and 16, 22 and 26 (1 each), with the mean number of CNVR per chromosome equal to 6.45, 4.27 and 6.14, respectively.

**Table 2:** CNVR numbers, lengths and coverage for each CNV detection method used

Software	Copy state	N^a^	CNVR length (bp)				Total (Mb)	Coverage (%)^b^
			Mean	Median	Min	Max		
*Brown Swiss*								
PennCNV	Loss	210	51,633.0	24,514.5	1358	483,799	10.843	0.43
	Gain	66	84,062.2	24,387.5	2809	1,879,682	5.548	0.22
	Both	30	241,581.7	104,390.5	7538	1,347,298	7.247	0.29
	Overall	306					23.638	0.94
SVS	Loss	141	41,221.0	6858.0	1086	1,217,387	5.812	0.23
	Both	46	37,702.4	10,232.5	1774	353,135	1.734	0.07
	Overall	187					7.546	0.30
Consensus	Loss	9	42,871.3	47,761.0	4693	90,545	0.386	0.02
	Both	21	197,095.7	53,252.0	1404	945,913	4.139	0.16
	Overall	30					4.525	0.18
*Tyrol Grey*								
PennCNV	Loss	502	95,870.1	49,568.0	1358	2,611,715	48.127	1.65
	Gain	90	47,216.2	24,954.0	2455	279,361	4.249	0.15
	Both	14	518,196.6	256,469.0	5035	1,646,040	7.255	0.25
	Overall	606					59.631	2.04
SVS	Loss	115	167,013.8	11,531.0	1369	4,210,187	19.207	0.66
	Both	38	44,509.8	11,567.0	1774	652,218	1.691	0.06
	Overall	153					20.898	0.72
Consensus	Loss	49	142,561.0	75,065.0	3620	1,432,454	6.985	0.24
	Both	22	205,619.5	58,194.5	2270	790,623	4.524	0.16
	Overall	71					11.509	0.39
*Pinzgauer*								
PennCNV	Loss	390	63,906.2	26,759.5	1300	951,876	24.923	0.99
	Gain	100	35,733.7	20,985.5	1950	279,361	3.573	0.14
	Both	38	373,093.4	164,810.5	4038	2,050,695	14.17755	0.56
	Overall	528					42.67433	1.70
SVS	Loss	119	66,791.9	8018.0	1169	1,311,740	7.94824	0.32
	Gain	1	307,583.0	307,583.0	307,583	307,583	0.307583	0.01
	Both	58	31,875.8	10,004.5	1369	320,050	1.848796	0.07
	Overall	178					10.10462	0.40
Consensus	Loss	17	66,484.7	31,556.0	4895	459,485	1.13024	0.05
	Both	33	179,520.3	34,711.0	1774	947,366	5.924169	0.24
	Overall	50					7.054409	0.28

^a^N = Number of CNVRs

^b^The coverage percentage is based on the bovine autosomal genome size of 2511 Mb covered by the BovineHD SNP chip

CNVR coverage was highest in Tyrol Grey, followed by Pinzgauer. The genome coverages for PennCNV CNVR and (SVS CNVR) were about 0.94% (0.30), 2.04% (0.72) and 1.70% (0.40) for Brown Swiss, Tyrol Grey and Pinzgauer, respectively. Jiang et al. [30] found that CNVR cover about 1.29% of the genome of the autosomes in Holsteins. Similarly, Wu et al. [31] reported a genome-wide coverage of 1.41% (about 35.48 Mb) in Simmental cattle. However, using sequence data, Keel et al. [32] found that CNV cover about 6.7% of the bovine genome.

All the consensus CNVR in Brown Swiss and most of those in Tyrol Grey and Pinzgauer have been reported in another Brown Swiss population by Prinsen et al. [20], while most of the consensus CNVR in Tyrol Grey and Pinzgauer have recently been reported by Bickhart et al. [33] and Sasaki et al. [34] (Table 3).

**Table 3:** Overlaps between the consensus CNVR identified in this study and CNVR reported by other studies

Study	Autosomal CNVR	Coverage (Mb)	Brown Swiss		Tyrol Grey		Pinzgauer	
			Overlaps	%	Overlaps	%	Overlaps	%
Bae et al. [35]	368	51.596	4	13	9	12	5	10
Bagnato et al. [36]	150	48.252	1	3	4	5	1	3
Bickhart et al. [33]	1726	51.396	22	73	33	44	39	78
Hou et al. [37]	3346	51.798	10	33	23	31	19	38
Liu et al. [38]	163	43.631	2	7	5	7	5	10
Prinsen et al. [20]	563	50.444	30	100	65	87	43	86
Sasaki et al. [34]	861	50.251	24	80	49	65	39	78
Wu et al. [31]	247	46.839	10	33	16	21	13	26
Xu et al. [39]	257	41.564	13	43	20	27	24	48
Zhang et al. [40]	425	49.037	14	47	24	32	25	50

Generally, SVS tended to detect CNV that occurred across individuals, while PennCNV tended to detect also CNV that were private to individuals. This may be due to PennCNV using additional individual-specific information such as BAF values, compared to SVS, which only uses LRR values, and because the CNAM algorithm in SVS detects fewer private CNV than its univariate counterpart.

### Gaps in SNP coverage

**Fig. 2:**
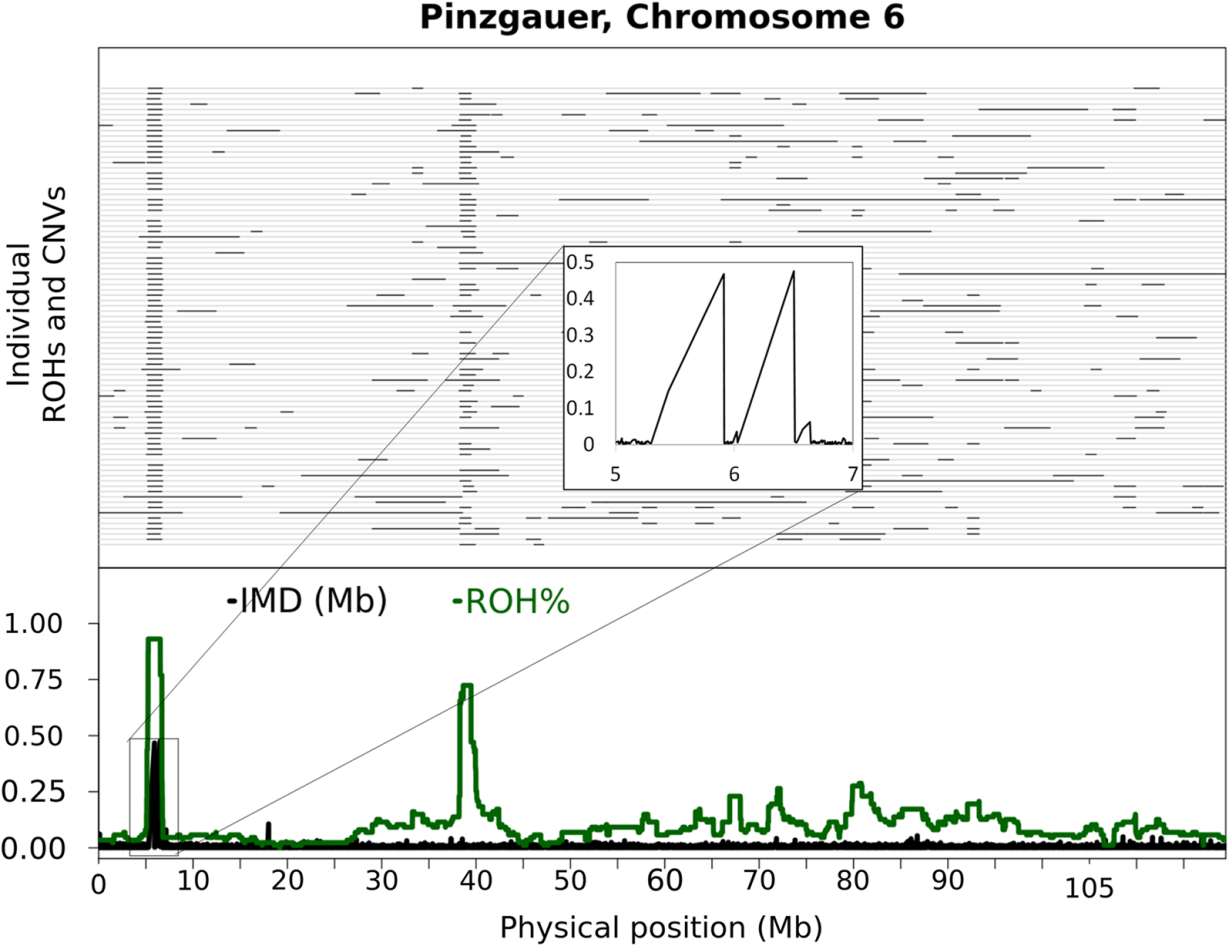
Details of the overlaps between SNP coverage gaps, ROH islands and CNVR. The upper panel shows the ROH (black) for each animal (grey gridlines). The lower panel shows the IMD (black) and the proportion of animals in ROH at each marker (green). The inset shows a short region with normal IMD, and which could be a true ROH island. However, the region is flanked by big gaps, the edges of which could also be true ROH. The whole region is detected as a ROH in most of the individuals

There were gaps in SNP coverage above the threshold of 9.2365 kb on many chromosomes, with the most significant ones being on BTA6, 7, 8, 10, 12, 13, 14, 15, 16, 17, 23, and 27. The largest gap detected was 1,080,181 bp long at position 7,798,579 on BTA7. Some of the gaps were in regions that contain ROH islands, such as the regions between approximately 5 and 7 Mb on BTA6 and between approximately 24 and 25 Mb on BTA10 in the three breeds. Such gaps in SNP coverage can lead the algorithms that are used for the detection of ROH to extend short ROH as illustrated by the first ROH island on BTA6 (Fig. 2), which is characteristic of some taurine breeds, but absent in indicine breeds [7]. This is most probably caused by the presence of very short gaps in the regions flanking the ROH, which leads the ROH algorithm to detect the whole region as one ROH. A similar pattern of gaps was observed on BTA12.

### Intersections between gaps in SNP coverage, CNVR and ROH islands

**Fig. 3:**
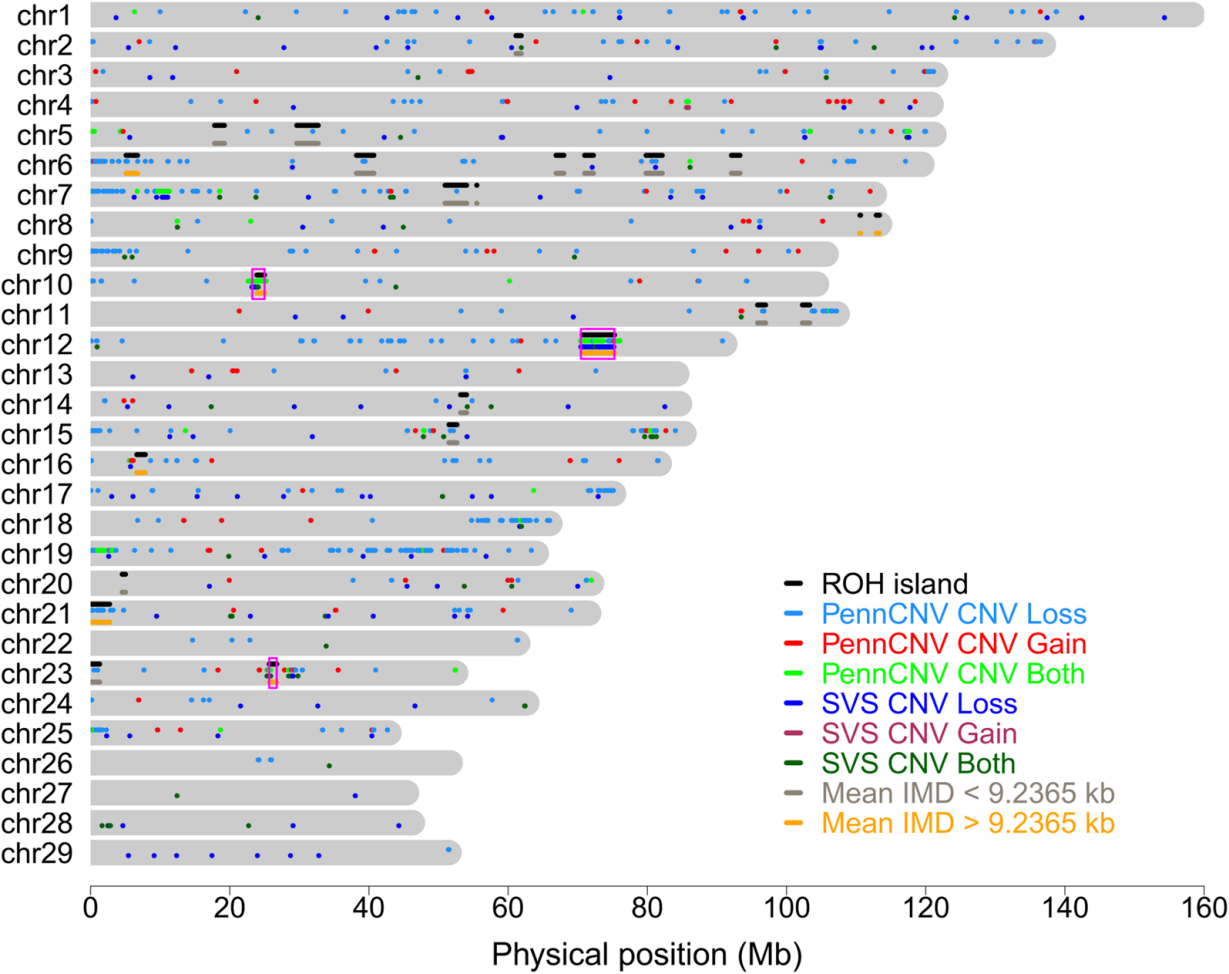
ROH islands and CNVR on each chromosome for Pinzgauer cattle. Each chromosome (dark grey bar) has four lines. Starting from top to bottom within the chromosome, the top line (black) is for ROH islands. The second line is for PennCNV CNV (light blue for copy loss, red for copy gain and light green for both copy loss and copy gain). The third line is for SVS CNV (blue for copy loss, maroon for copy gain and dark green for both copy loss and copy gain). The fourth (last) line is for intermarker distance (IMD, light grey for IMD < 9.2365 kb and orange for IMD > 9.2365 kb). The magenta rectangles show regions where consensus CNVR overlap with ROH islands

Details of the overlaps between individual ROH and individual CNV for each animal and each chromosome in the three breeds are in Additional file 3. A summary of the ROH islands and CNVR that were detected by both PennCNV and SVS in Pinzgauer cattle is in Fig. 3, which shows the overlaps between ROH islands, CNVR and IMD. Similar figures for such overlaps in the other two breeds are in Figure S3 (see Additional file 4: Figure S3).

For Tyrol Grey, an overlap was detected between a ROH island and a consensus CNVR (copy loss) on BTA17 (with the intersection at 73,748,297–75,132,928 bp). In the three breeds, at least one consensus CNVR intersected with a ROH island and/or a gap on BTA10. For Brown Swiss, the intersection was within a gap between 23,889,533 and 24,998,515 bp. For Tyrol Grey, the intersection was within both gaps and CNV at two locations: between 23,651,168 and 24,057,642 bp and between 24,061,376 and 24,143,260 bp. For Pinzgauer, the overlap was also within both gaps and consensus CNVR at two locations: between 23,889,533 and 24,057,642 bp and between 24,061,376 and 24,095,827 bp. We note that the region between 23 and 25 Mb on BTA10 contains the *T*-*cell receptor alpha chain V* gene and it has been reported that CNVR are strongly associated with genes responsible for immune response [41].

For the three breeds, intersections between ROH islands and a combination of gaps and consensus CNVR were found on BTA12 between approximately 70.0 and 75.5 Mb. For Brown Swiss, only one CNVR was detected in this region i.e. between 72,432,362 and 72,467,225 bp with the corresponding ROH island at 72,432,362–72,467,225 bp and intersection at 72,432,362–72,467,225 bp. For Tyrol Grey and Pinzgauer, the ROH islands present in this region were longer (4.6 and 4.7 Mb, respectively), and overlapped with multiple CNVR (11 and 12, respectively). The existence of CNV on BTA12 in taurine cattle has been documented [30]. We observed one intersection between ROH islands, gaps and consensus CNVR on BTA23 for Pinzgauer and also several other overlaps between gaps, ROH islands and CNVR detected by either PennCNV or SVS alone.

The results on the intersections between CNVR and ROH islands based on the test data (ROHD) and the mean intersections between the CNVR and ROH islands with randomized positions (ROHR) are in Table 4. ROHR differed considerably between the two algorithms used to detect CNV for Brown Swiss but were similar for Tyrol Grey and Pinzgauer. ROHR were generally lower than ROHD (*P* < 0.001), which implies that the intersections between the ROH islands and the CNV are not random and that there is a significant association between the ROH islands and the CNVR.

**Table 4:** Results of the permutation test that checks whether the intersections between CNVR with copy loss (copy loss or both copy loss and copy gain) and ROH islands are due to chance alone

Breed	Software	ROHD^a^	Estimate (ROHR^b^)	Confidence interval		*P* value
				Lower	Upper	
Brown Swiss	PennCNV	0.630	0.072	0.069	0.074	0
	SVS	0.176	0.023	0.021	0.025	0
	Consensus	0.000	0.008	0.007	0.009	7.00E−38
Tyrol Grey	PennCNV	2.931	0.460	0.451	0.470	0
	SVS	2.453	0.150	0.143	0.157	0
	Consensus	2.135	0.084	0.079	0.088	0
Pinzgauer	PennCNV	4.824	0.420	0.410	0.430	0
	SVS	3.774	0.059	0.056	0.063	0
	Consensus	2.729	0.033	0.030	0.036	0

The number of iterations used for randomizing the locations of the ROH islands was 10,000

^a^Intersections between CNVRs and ROH islands from the data

^b^Intersections between CNVRs and randomized ROH islands

### Falsely identified ROH islands

Details of the position of individual ROH islands, proportions of individuals with CNV at each marker and the proportion of inbred individuals and percentage of individuals with copy gain or loss within each ROH island for the CNVR detected with PennCNV and SVS and for consensus CNVR are in Table S4 (see Additional file 1: Table S4). Seven, 14 and 16 ROH islands were identified as possibly false for Brown Swiss, Tyrol Grey and Pinzgauer, respectively. Table S5 shows the details of the 37 ROH islands considered as false based on the overlaps between CNVR and ROH islands and the mean IMD within the ROH islands, and Table 5 shows a summary of the number and sizes of the ROH islands. For Brown Swiss cattle, 48% (6.624 Mb) of the 13.928 Mb affected by CNV in ROH islands were located on BTA16 between 22,077,094 and 28,701,552 bp. Similarly, stretches of ROH islands of about 4.612 and 4.652 Mb for Tyrol Grey and Pinzgauer, respectively, which were affected by CNV and gaps, were on BTA12 between 70 and 75 Mb and for Pinzgauer, most of the ROH islands were affected. For Brown Swiss, about 37% of the ROH islands were affected by copy loss. Similarly, the proportion of ROH island coverage affected by copy loss and coverage gaps was equal to 44 and 52%, for Tyrol Grey and Pinzgauer, respectively.

**Table 5:** Numbers and lengths ROH islands that were affected by CNV and gaps

Breed	ROH islands affected by	Number of affected ROH islands	Coverage (Mb)	As the percentage of total ROH island coverage
Brown Swiss (ROH island coverage = 26.637)	Gain + loss	3	4.073	15.3
	Gap	1	1.459	5.5
	Gap + gain + loss	1	1.109	4.2
	Loss	2	9.855	37.0
	Overall	7	16.496	61.9
Tyrol Grey (ROH island coverage = 35.847)	Gain	1	1.417	4.0
	Gap	3	4.225	11.8
	Gap + gain + loss	2	5.789	16.1
	Gap + loss	2	2.931	8.2
	Loss	6	12.939	36.1
	Overall	14	27.301	76.2
Pinzgauer (ROH island coverage = 38.978)	Gap	3	1.811	4.6
	Gap + gain + loss	3	6.834	17.5
	Gap + loss	2	4.382	11.2
	Loss	8	15.879	40.7
	Overall	16	28.905	74.2

### Evidence from BAF and LRR plots

**Fig. 4:**
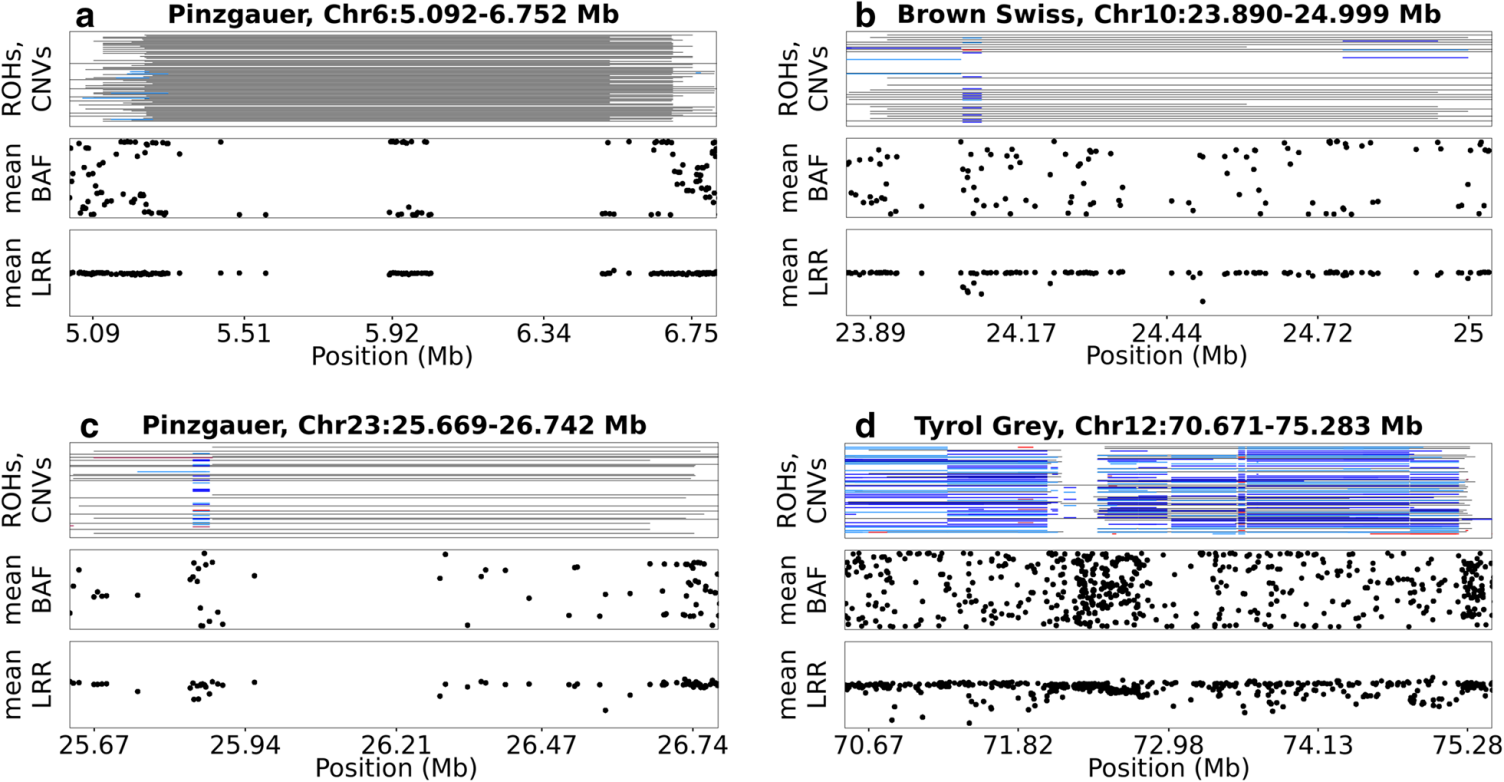
BAF and LRR plots of selected ROH islands. In each of the four sub-plots, the top panel is for individual ROH (black) and individual CNV (blue and dark red for copy loss and copy gain, respectively, for SVS; and light blue and red for copy loss and copy gain, respectively, for PennCNV). The middle panel shows the mean BAF values at each marker while the third panel is for mean LRR at each marker. Panel **a** illustrates the possibility of a gap being detected as a ROH. Panel **b** shows how a CNVR can extend ROH leading to a ROH island. Panel **c** is a typical example of a CNVR and gaps within a ROH island. Panel **d** is an extreme example of ROH islands being detected between regions with a high frequency of CNV

The BAF and LRR plots for all 42 ROH islands are in Additional file 5. Figure 4 shows the genetic mechanisms that may be responsible for the false ROH islands based on the distributions of BAF values and LRR values in selected ROH islands. The plots show that some of the ROH islands identified in this study could indeed be artefacts due to coverage gaps and mistyping of genotypes because of the presence of CNV.

### Haplotype diversity within ROH islands and intersections of ROH islands and CNVR with copy losses

**Fig. 5:**
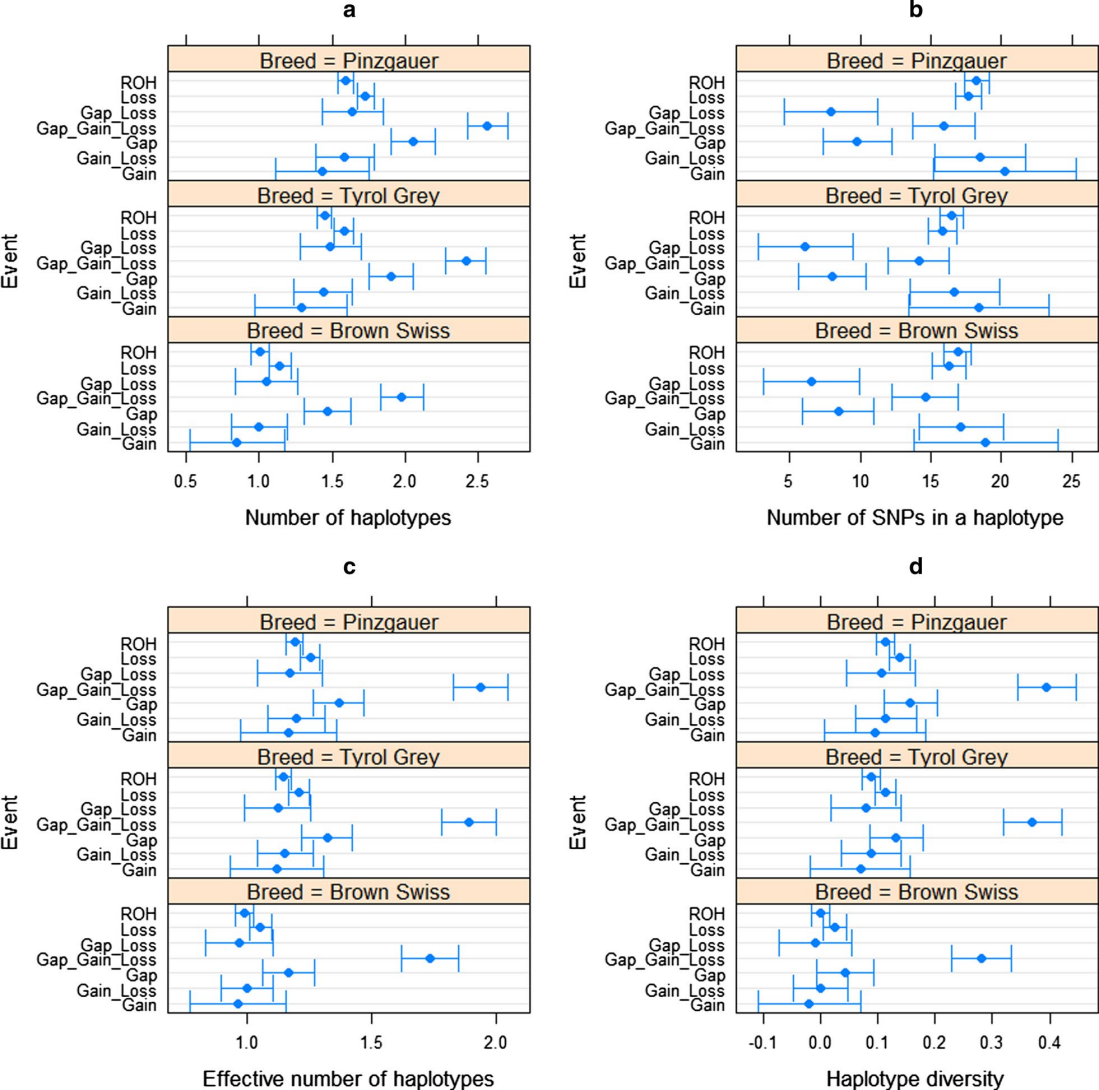
Haplotype diversity statistics in all ROH islands. **a** Number of haplotypes. **b** Number of SNPs per haplotype. **c** Effective number of haplotypes per haplotype block. **d** Haplotype diversity

There were significant differences in haplotype numbers between different categories of ROH islands and between breeds (*P* = 2.2e−16). The number of SNPs per haplotype differed between breeds (*P* = 0.004) and varied widely across the ROH island categories (*P* = 1.418e−15). The effective number of haplotypes was affected by both breed and ROH island category (*P* = 2.2e−16). Haplotype diversity was lowest in ROH islands with both copy loss and copy gain (such as on BTA12 between 70 and 75 Mb). Haplotype diversity was lower in ROH islands with no gaps or CNV than in ROH islands with coverage gaps, copy loss and CNV. The diversity of the rest of the ROH islands with gaps and/or copy loss and/or copy gain was similar to that of ROH islands with none of these issues. Figure 5 shows the estimates of the number of haplotypes per block, number of SNPs per haplotype, effective numbers of haplotypes and haplotype diversity in the ROH islands.

CNV contribute significantly to genetic variation [42] and have been associated with several genetic disorders. Under natural selection, individuals with undesirable SV are unlikely to pass on their genes to their offspring. However, most SV are benign. Moreover, if the SV confer some advantage to the individual, balancing selection may occur. This is very common in domestic livestock such as cattle, where breeders deliberately select breeding stock for particular traits, some of which may result from single copy genotypes, which may result in SV being maintained in a large proportion of the individuals beyond what could be expected from genetic drift alone [43]. If such variants are copy number deletions, there may be a high frequency of heterozygous wild type/null genotypes [44], which could be mistyped as homozygotes by the GenTrain algorithm [15]. Subsequently, the ROH calling algorithms would interpret such regions as ROH, which for the population would lead to erroneous ROH islands.

## Conclusions

ROH contain valuable information for estimating the levels of inbreeding, predicting and mapping inbreeding depression and for identifying signatures of selection. In this paper, we present evidence indicating that some of the ROH islands in the bovine genome may be artefacts due to copy losses as well as to coverage gaps (~ 37, 44 and 52% of the genomic regions covered by ROH islands for Brown Swiss, Tyrol Grey and Pinzgauer, respectively). Thus, CNV and coverage gaps need to be taken into proper account and considered with great care when assessing signatures of selection via ROH patterns.

## Additional files

**Additional file 1: Table S1.** Descriptive statistics of inbreeding levels based on the sum of merged ROH for each breed. **Table S2.** List of ROH islands for the three breeds. **Table S3.** List of CNVR for the three breeds. **Table S4.** Details of the positions of individual ROH islands, proportions of individuals with CNV at each marker and proportion of inbred individuals and percentage of individuals with copy gain or loss in each ROH island. **Table S5.** Description of the ROH islands that were identified as artefacts due to coverage gaps and consensus CNVR with both copy loss (copy loss or both copy loss and copy gain).

**Additional file 2: Figure S1.** Manhattan plot showing ROH islands in Brown Swiss, Tyrol Grey and Pinzgauer cattle.

**Additional file 3: Figure S2-1:87.** Details of the overlaps between individual ROH and individual CNV for each animal and each chromosome in the three breeds. (a) the grey line indicates an animal and a black line on the grey line represents ROH for that animal. Below the grey line are the CNV for the animal with the following color codes: light blue and red for copy loss and copy gain according to PennCNV, respectively, and dark red and dark blue for copy loss and copy gain according to SVS. (b) Mean intermarker distance (IMD, black) and proportions of individuals in a ROH (dark green) and in a CNV according to SVS (magenta) or PennCNV (cyan).

**Additional file 4: Figure S3.** ROH islands and CNVR for each chromosome in Pinzgauer cattle. Each chromosome (dark grey bar) has four lines. Starting from top to bottom within the chromosome, the top line (black) is for ROH islands. The second line is for PennCNV CNV (light blue for copy loss, red for copy gain and light green for both copy loss and copy gain). The third line is for SVS CNV (blue for copy loss, maroon for copy gain and dark green for both copy loss and copy gain). The fourth (last) line is for intermarker distance (IMD, light grey for IMD < 9.2365 kb and orange for IMD > 9.2365 kb). The magenta rectangles show regions where consensus CNVR overlap with ROH islands.

**Additional file 5: Figure S4.** BAF and LRR plot for each of the 57 ROH islands. In each sub-plot, the top panel is for individual ROH (black) and individual CNV (blue and dark red for copy loss and copy gain, respectively, for SVS and light blue and red for copy loss and copy gain, respectively, for PennCNV). The middle panel shows the mean BAF values at each marker while the third panel is for mean LRR at each marker.

## References

[1] Purfield DC, Berry DP, McParland S, Bradley DG (2012). Runs of homozygosity and population history in cattle. BMC Genet.

[2] Howrigan DP, Simonson MA, Keller MC (2011). Detecting autozygosity through runs of homozygosity: a comparison of three autozygosity detection algorithms. BMC Genomics..

[3] Curik I, Ferenčaković M, Sölkner J (2014). Inbreeding and runs of homozygosity: a possible solution to an old problem. Livest Sci.

[4] Sölkner J, Ferenčaković M, Gredler-Grandl B, Čurik I. Genomic metrics of individual autozygosity, applied to a cattle population. In: Proceedings of the 61st annual meeting of the European Association of Animal Production: Heraklion; 23–27 August 2010; 2010.

[5] Saura M, Fernández A, Varona L, Fernández AI, de Cara MÁR, Barragán C (2015). Detecting inbreeding depression for reproductive traits in Iberian pigs using genome-wide data. Genet Sel Evol.

[6] Zavarez LB, Utsunomiya YT, Carmo AS, Neves HHR, Carvalheiro R, Ferencakovic M (2015). Assessment of autozygosity in Nellore cows (*Bos indicus*) through high-density SNP genotypes. Front Genet.

[7] Karimi Z. Runs of homozygosity patterns in taurine and indicine cattle breeds. Master Thesis, BOKU University; 2013.

[8] Ferenčaković M, Sölkner J, Curik I (2013). Estimating autozygosity from high-throughput information: effects of SNP density and genotyping errors. Genet Sel Evol.

[9] Peripolli E, Munari DP, Silva MVGB, Lima ALF, Irgang R, Baldi F (2017). Runs of homozygosity: current knowledge and applications in livestock. Anim Genet.

[10] Szmatola T, Gurgul A, Ropka-Molik K, Jasielczuk I, Zabek T, Bugno-Poniewierska M (2016). Characteristics of runs of homozygosity in selected cattle breeds maintained in Poland. Livest Sci.

[11] Nothnagel M, Lu TT, Kayser M, Krawczak M (2010). Genomic and geographic distribution of snpdefined runs of homozygosity in Europeans. Hum Mol Genet.

[12] Ferenčaković M, Hamzić E, Gredler B, Solberg TR, Klemetsdal G, Curik I (2013). Estimates of autozygosity derived from runs of homozygosity: empirical evidence from selected cattle populations. J Anim Breed Genet.

[13] Alkan C, Coe BP, Eichler EE (2011). Genome structural variation discovery and genotyping. Nat Rev Genet.

[14] Redon R, Ishikawa S, Fitch KR, Feuk L, Perry GH, Andrews TD (2006). Global variation in copy number in the human genome. Nature.

[15] Ritchie ME, Liu R, Carvalho BS (2011). Australia and New Zealand Multiple Sclerosis Genetics Consortium (ANZgene), Irizarry R. Comparing genotyping algorithms for Illumina’s Infinium whole-genome SNP BeadChips. BMC Bioinformatics..

[16] Purcell S, Neale B, Todd-Brown K, Thomas L, Ferreira MAR, Bender D (2007). PLINK: a tool set for whole-genome association and population-based linkage analyses. Am J Hum Genet.

[17] Zhang L, Orloff MS, Reber S, Li S, Zhao Y, Eng C (2013). cgaTOH: extended approach for identifying tracts of homozygosity. PLoS One..

[18] Quinlan AR, Hall IM (2010). BEDTools: a flexible suite of utilities for comparing genomic features. Bioinformatics.

[19] Wang K, Li M, Hadley D, Liu R, Glessner J, Grant SFA (2007). PennCNV: an integrated hidden Markov model designed for high-resolution copy number variation detection in whole-genome SNP genotyping data. Genome Res.

[20] Prinsen RTMM, Strillacci MG, Schiavini F, Santus E, Rossoni A, Maurer V (2016). A genome-wide scan of copy number variants using high-density SNPs in Brown Swiss dairy cattle. Livest Sci.

[21] Zhou Y, Utsunomiya YT, Xu L, el Hay HA, Bickhart DM, Sonstegard TS (2016). Comparative analyses across cattle genders and breeds reveal the pitfalls caused by false positive and lineage-differential copy number variations. Sci Rep.

[22] Kent WJ, Zweig AS, Barber G, Hinrichs AS, Karolchik D (2010). BigWig and BigBed: enabling browsing of large distributed datasets. Bioinformatics.

[23] Cox NJ, Jones K, Wrigley N, Bennett RJ (1981). Exploratory data analysis. Quantitative geography.

[24] Ghani M, Pinto D, Lee JH, Grinberg Y, Sato C, Moreno D (2012). Genome-wide survey of large rare copy number variants in Alzheimer’s disease among Caribbean hispanics. G3 (Bethesda).

[25] Delaneau O, Coulonges C, Zagury JF (2008). Shape-IT: new rapid and accurate algorithm for haplotype inference. BMC Bioinformatics..

[26] Utsunomiya YT, Milanesi M, Utsunomiya ATH, Ajmone-Marsan P, Garcia JF (2016). GHap: an R package for genome-wide haplotyping. Bioinformatics.

[27] R Core Team (2013). R: A language and environment for statistical computing.

[28] Villa-Angulo R, Matukumalli LK, Gill CA, Choi J, Van Tassell CP, Grefenstette JJ (2009). High-resolution haplotype block structure in the cattle genome. BMC Genet.

[29] Mokry FB, Buzanskas ME, de Alvarenga Mudadu M, do Amaral Grossi D, Higa RH, Ventura RV (2014). Linkage disequilibrium and haplotype block structure in a composite beef cattle breed. BMC Genomics.

[30] Jiang L, Jiang J, Yang J, Liu X, Wang J, Wang H (2013). Genome-wide detection of copy number variations using high-density SNP genotyping platforms in Holsteins. BMC Genomics.

[31] Wu Y, Fan H, Jing S, Xia J, Chen Y, Zhang L (2015). A genome-wide scan for copy number variations using high-density single nucleotide polymorphism array in Simmental cattle. Anim Genet.

[32] Keel BN, Keele JW, Snelling WM (2016). Genome-wide copy number variation in the bovine genome detected using low coverage sequence of popular beef breeds. Anim Genet.

[33] Bickhart DM, Xu L, Hutchison JL, Cole JB, Null DJ, Schroeder SG (2016). Diversity and population-genetic properties of copy number variations and multicopy genes in cattle. DNA Res.

[34] Sasaki S, Watanabe T, Nishimura S, Sugimoto Y (2016). Genome-wide identification of copy number variation using high-density single-nucleotide polymorphism array in Japanese Black cattle. BMC Genet.

[35] Bae J, Cheong H, Kim L, NamGung S, Park T, Chun JY (2010). Identification of copy number variations and common deletion polymorphisms in cattle. BMC Genomics.

[36] Bagnato A, Strillacci MG, Pellegrino L, Schiavini F, Frigo E, Rossoni A (2015). Identification and validation of copy number variants in Italian Brown Swiss dairy cattle using Illumina Bovine SNP50 Beadchip^®^. Ital J Anim Sci.

[37] Hou Y, Bickhart DM, Hvinden ML, Li C, Song J, Boichard DA (2012). Fine mapping of copy number variations on two cattle genome assemblies using high density SNP array. BMC Genomics.

[38] Liu GE, Hou Y, Zhu B, Cardone MF, Jiang L, Cellamare A (2010). Analysis of copy number variations among diverse cattle breeds. Genome Res.

[39] Xu L, Hou Y, Bickhart DM, Zhou Y, el Hay HA, Song J (2016). Population-genetic properties of differentiated copy number variations in cattle. Sci Rep.

[40] Zhang Q, Ma Y, Wang X, Zhang Y, Zhao X (2015). Identification of copy number variations in Qinchuan cattle using BovineHD Genotyping Beadchip array. Mol Genet Genomics.

[41] Sharp AJ, Cheng Z, Eichler EE (2006). Structural variation of the human genome. Annu Rev Genom Hum Genet.

[42] Malhotra D, Sebat J (2012). CNVs: Harbingers of a rare variant revolution in psychiatric genetics. Cell.

[43] Charlesworth D (2006). Balancing selection and its effects on sequences in nearby genome regions. PLoS Genet.

[44] McQuillan R, Leutenegger AL, Abdel-Rahman R, Franklin CS, Pericic M, Barac-Lauc L (2008). Runs of homozygosity in European populations. Am J Hum Genet.

