# Supplementary figures and images for: Misidentification of runs of homozygosity islands in cattle caused by interference with copy number variation or large intermarker distances
Source: Genet Sel Evol. 2018 Aug 22;50:43. doi: 10.1186/s12711-018-0414-x (PMC6106898; doi:10.1186/s12711-018-0414-x)

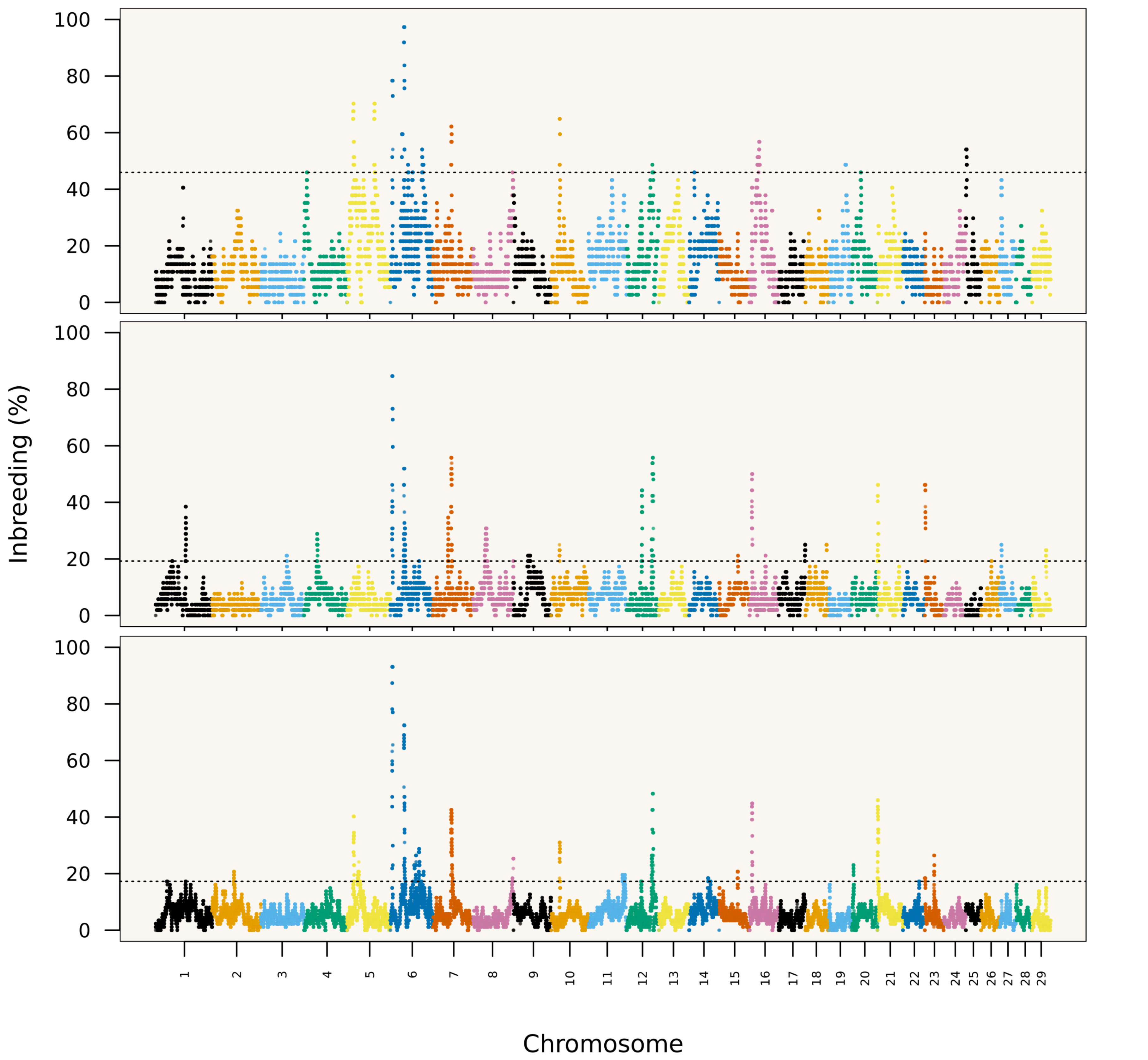

Supplement: Supplementary file 2 — Additional file 2: Figure S1. Manhattan plot showing ROH islands in Brown Swiss, Tyrol Grey and Pinzgauer cattle. [file 12711_2018_414_MOESM2_ESM.png]

Brown Swiss

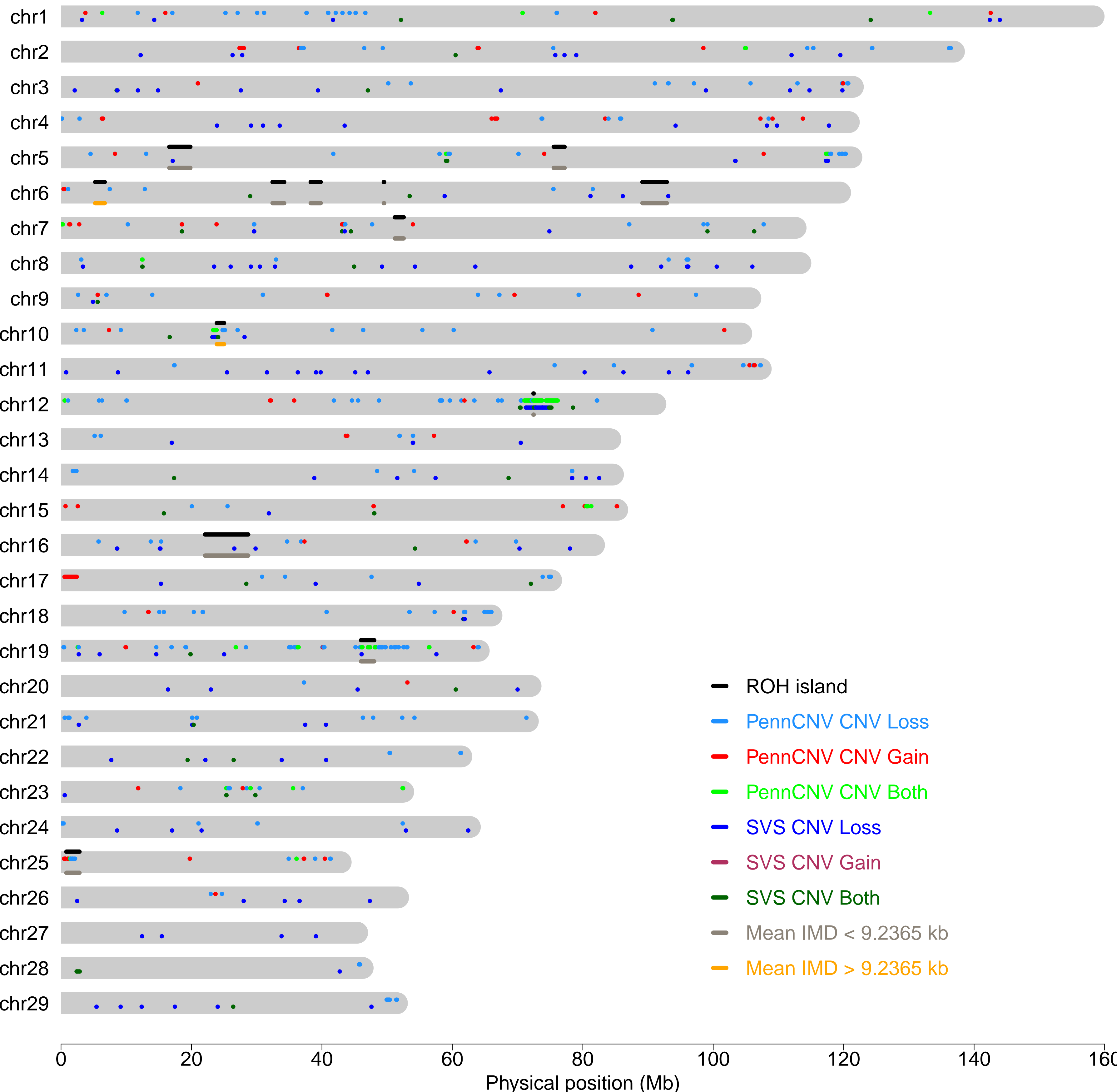

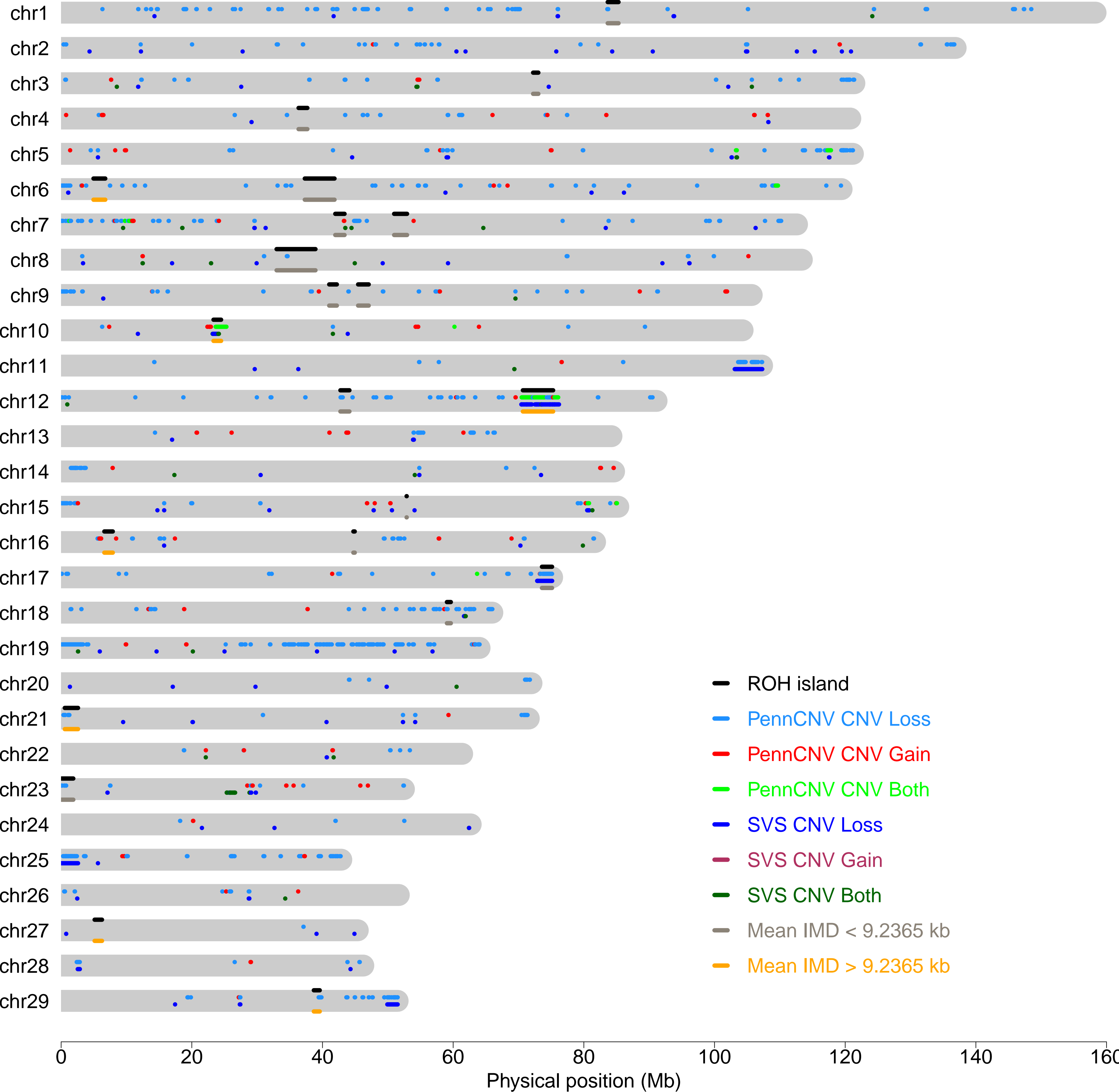

Supplement: Supplementary file 4 — Additional file 4: Figure S3. ROH islands and CNVR for each chromosome in Pinzgauer cattle. Each chromosome (dark grey bar) has four lines. Starting from top to bottom within the chromosome, the top line (black) is for ROH islands. The second line is for PennCNV CNV (light blue for copy loss, red for copy gain and light green for both copy loss and copy gain). The third line is for SVS CNV (blue for copy loss, maroon for copy gain and dark green for both copy loss and copy gain). The fourth (last) line is for intermarker distance (IMD, light grey for IMD < 9.2365 kb and orange for IMD > 9.2365 kb). The magenta rectangles show regions where consensus CNVR overlap with ROH islands. [file 12711_2018_414_MOESM4_ESM.pdf]
