# Supplementary material for: Misidentification of runs of homozygosity islands in cattle caused by interference with copy number variation or large intermarker distances
Source: Genet Sel Evol. 2018 Aug 22;50:43. doi: 10.1186/s12711-018-0414-x (PMC6106898; doi:10.1186/s12711-018-0414-x)
Supplement: Supplementary file 3 — Additional file 3: Figure S2-1:87. Details of the overlaps between individual ROH and individual CNV for each animal and each chromosome in the three breeds. (a) the grey line indicates an animal and a black line on the grey line represents ROH for that animal. Below the grey line are the CNV for the animal with the following color codes: light blue and red for copy loss and copy gain according to PennCNV, respectively, and dark red and dark blue for copy loss and copy gain according to SVS. (b) Mean intermarker distance (IMD, black) and proportions of individuals in a ROH (dark green) and in a CNV according to SVS (magenta) or PennCNV (cyan). [file 12711_2018_414_MOESM3_ESM.pdf]

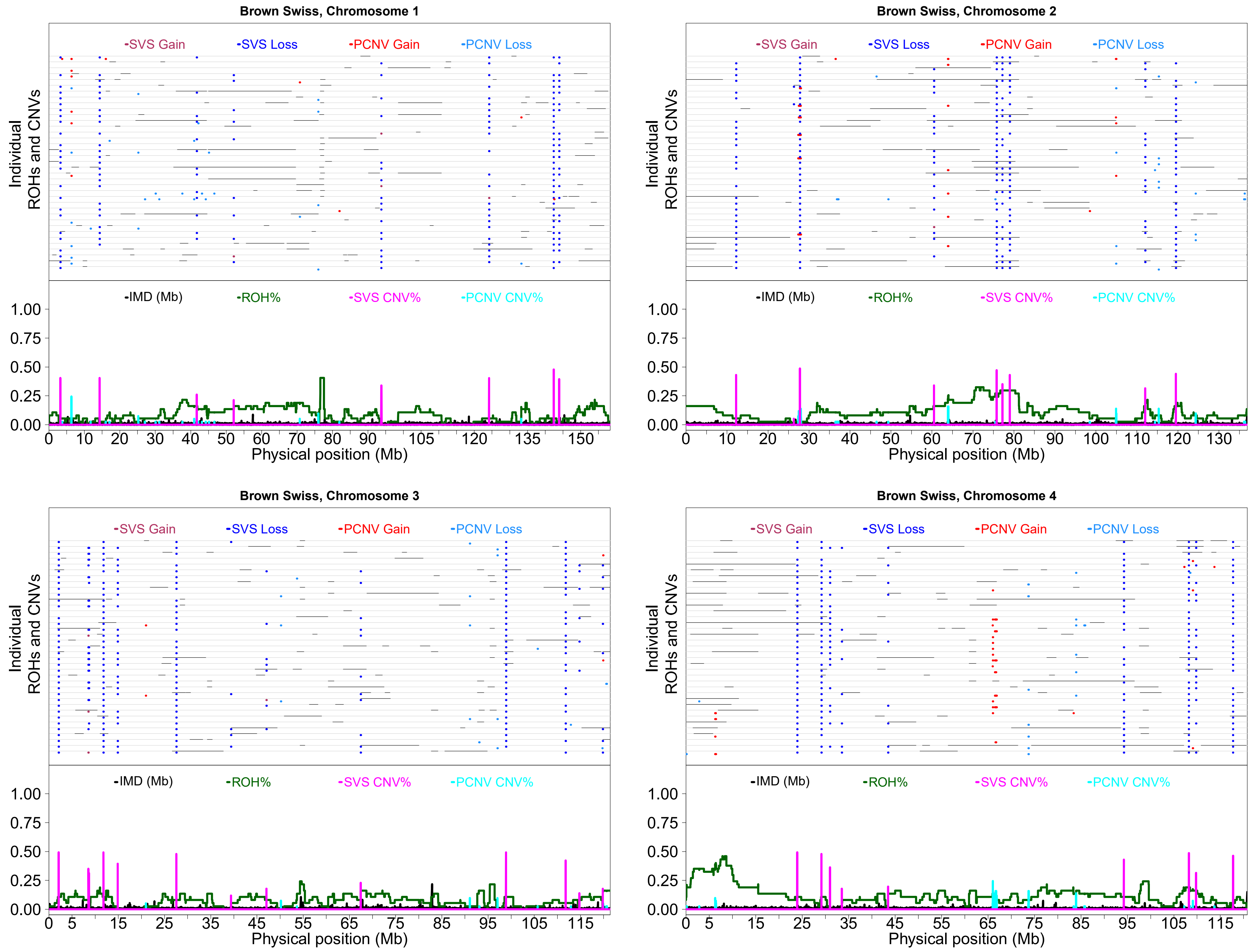
Brown Swiss, Chromosome 1
SVS Gain
SVS Loss
PCNV Gain
PCNV Loss
Individual ROHs and CNVs
IMD (Mb)
ROH%
SVS CNV%
PCNV CNV%
1.00
0.75
0.50
0.25
0.00
0 10 20 30 40 50 60 70 80 90 105 120 135 150
Physical position (Mb)
Brown Swiss, Chromosome 2
SVS Gain
SVS Loss
PCNV Gain
PCNV Loss
Individual ROHs and CNVs
IMD (Mb)
ROH%
SVS CNV%
PCNV CNV%
1.00
0.75
0.50
0.25
0.00
0 10 20 30 40 50 60 70 80 90 100 110 120 130
Physical position (Mb)
Brown Swiss, Chromosome 3
SVS Gain
SVS Loss
PCNV Gain
PCNV Loss
Individual ROHs and CNVs
IMD (Mb)
ROH%
SVS CNV%
PCNV CNV%
1.00
0.75
0.50
0.25
0.00
0 5 15 25 35 45 55 65 75 85 95 105 115
Physical position (Mb)
Brown Swiss, Chromosome 4
SVS Gain
SVS Loss
PCNV Gain
PCNV Loss
Individual ROHs and CNVs
IMD (Mb)
ROH%
SVS CNV%
PCNV CNV%
1.00
0.75
0.50
0.25
0.00
0 5 15 25 35 45 55 65 75 85 95 105 115
Physical position (Mb)

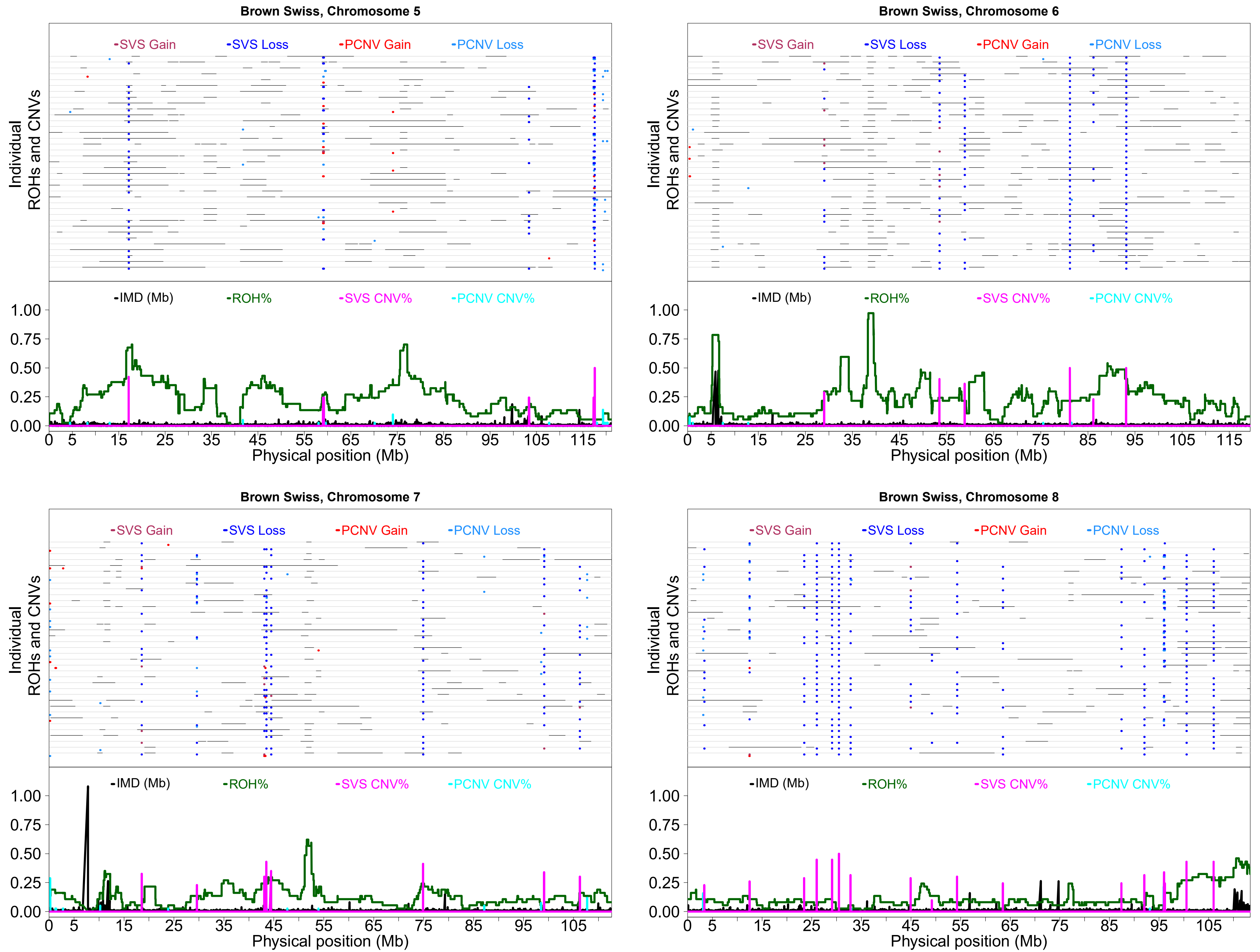

Brown Swiss, Chromosome 5
SVS Gain
SVS Loss
PCNV Gain
PCNV Loss
Individual ROHs and CNVs
IMD (Mb)
ROH%
SVS CNV%
PCNV CNV%
1.00
0.75
0.50
0.25
0.00
0 5 15 25 35 45 55 65 75 85 95 105 115
Physical position (Mb)
Brown Swiss, Chromosome 6
SVS Gain
SVS Loss
PCNV Gain
PCNV Loss
Individual ROHs and CNVs
IMD (Mb)
ROH%
SVS CNV%
PCNV CNV%
1.00
0.75
0.50
0.25
0.00
0 5 15 25 35 45 55 65 75 85 95 105 115
Physical position (Mb)
Brown Swiss, Chromosome 7
SVS Gain
SVS Loss
PCNV Gain
PCNV Loss
Individual ROHs and CNVs
IMD (Mb)
ROH%
SVS CNV%
PCNV CNV%
1.00
0.75
0.50
0.25
0.00
0 5 15 25 35 45 55 65 75 85 95 105
Physical position (Mb)
Brown Swiss, Chromosome 8
SVS Gain
SVS Loss
PCNV Gain
PCNV Loss
Individual ROHs and CNVs
IMD (Mb)
ROH%
SVS CNV%
PCNV CNV%
1.00
0.75
0.50
0.25
0.00
0 5 15 25 35 45 55 65 75 85 95 105
Physical position (Mb)

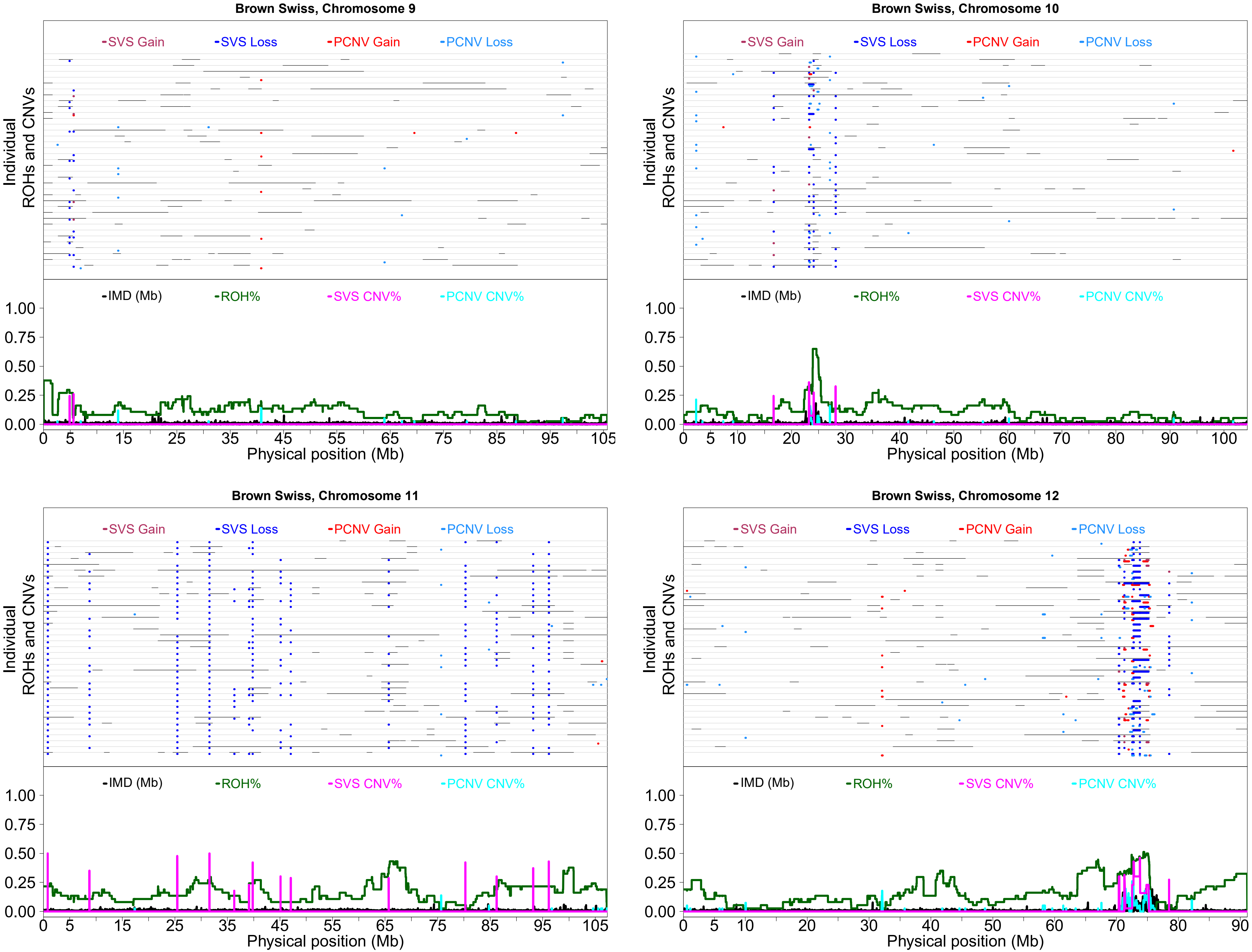
Brown Swiss, Chromosome 9
Brown Swiss, Chromosome 10
Brown Swiss, Chromosome 11
Brown Swiss, Chromosome 12
SVS Gain
SVS Loss
PCNV Gain
PCNV Loss
Individual ROHs and CNVs
IMD (Mb)
ROH%
SVS CNV%
PCNV CNV%
Physical position (Mb)

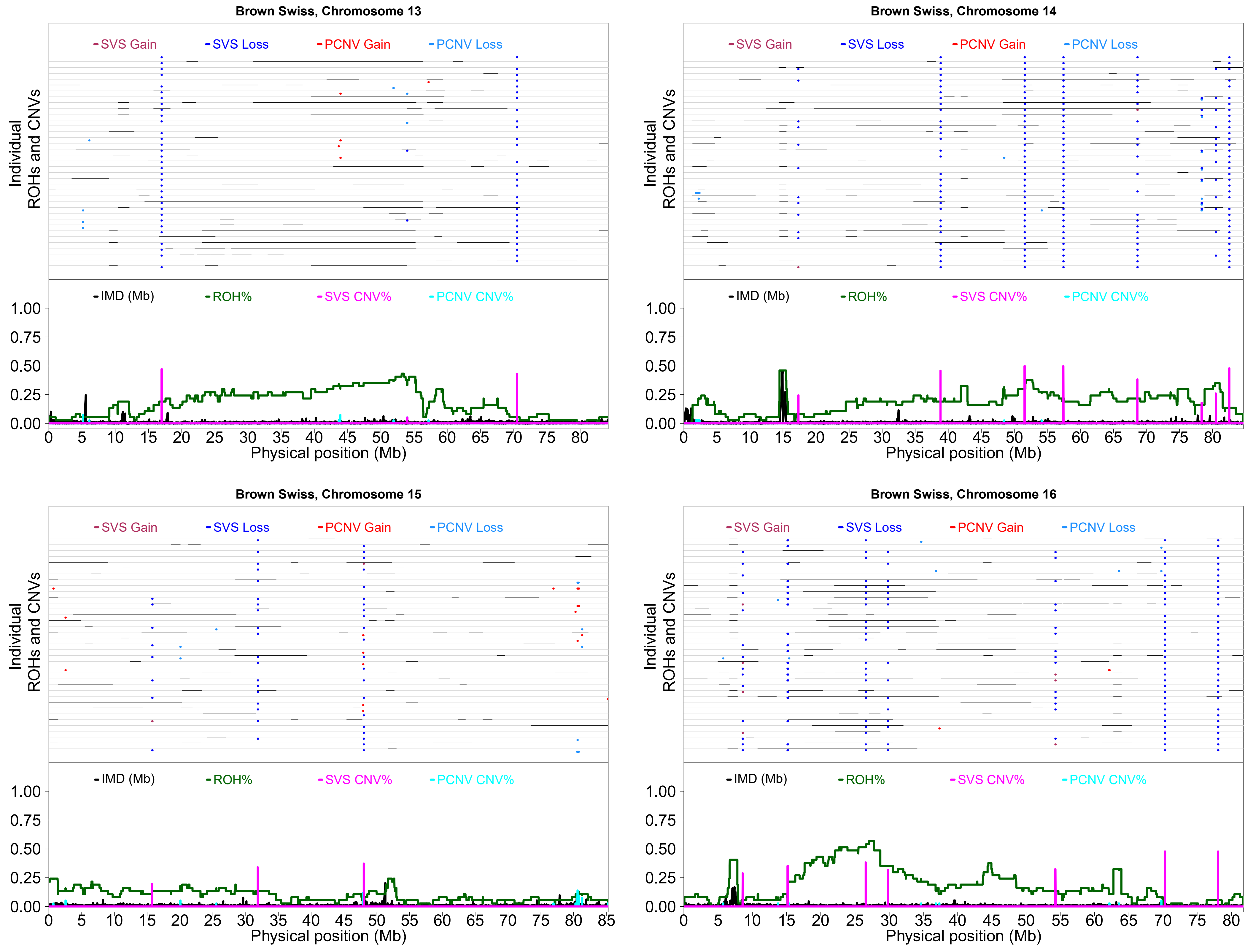

Brown Swiss, Chromosome 13
Brown Swiss, Chromosome 14
Brown Swiss, Chromosome 15
Brown Swiss, Chromosome 16
SVS Gain
SVS Loss
PCNV Gain
PCNV Loss
Individual ROHs and CNVs
IMD (Mb)
ROH%
SVS CNV%
PCNV CNV%
Physical position (Mb)

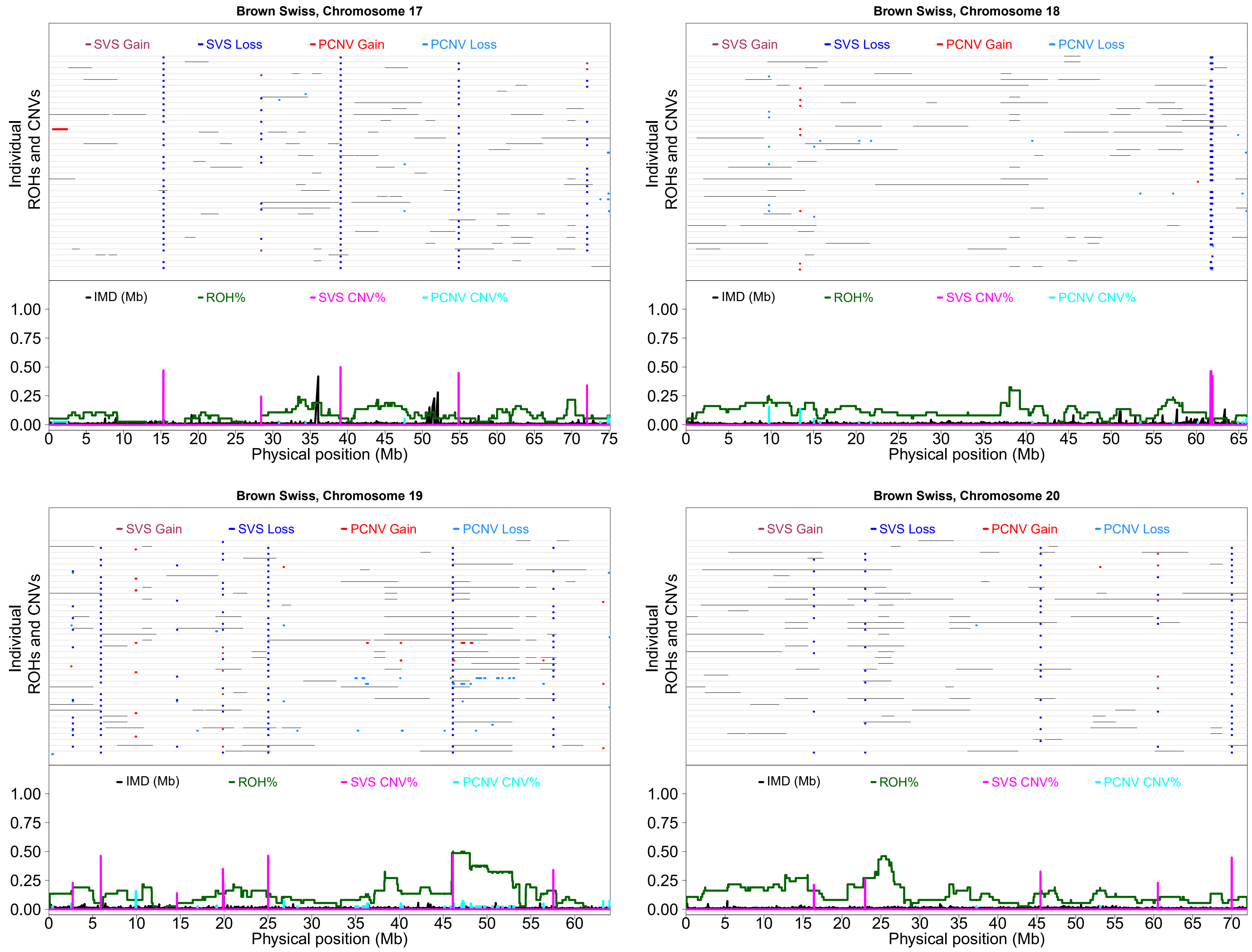

Brown Swiss, Chromosome 17
SVS Gain
SVS Loss
PCNV Gain
PCNV Loss
Individual ROHs and CNVs
IMD (Mb)
ROH%
SVS CNV%
PCNV CNV%
1.00
0.75
0.50
0.25
0.00
0 5 10 15 20 25 30 35 40 45 50 55 60 65 70 75
Physical position (Mb)
Brown Swiss, Chromosome 18
SVS Gain
SVS Loss
PCNV Gain
PCNV Loss
Individual ROHs and CNVs
IMD (Mb)
ROH%
SVS CNV%
PCNV CNV%
1.00
0.75
0.50
0.25
0.00
0 5 10 15 20 25 30 35 40 45 50 55 60 65
Physical position (Mb)
Brown Swiss, Chromosome 19
SVS Gain
SVS Loss
PCNV Gain
PCNV Loss
Individual ROHs and CNVs
IMD (Mb)
ROH%
SVS CNV%
PCNV CNV%
1.00
0.75
0.50
0.25
0.00
0 5 10 15 20 25 30 35 40 45 50 55 60
Physical position (Mb)
Brown Swiss, Chromosome 20
SVS Gain
SVS Loss
PCNV Gain
PCNV Loss
Individual ROHs and CNVs
IMD (Mb)
ROH%
SVS CNV%
PCNV CNV%
1.00
0.75
0.50
0.25
0.00
0 5 10 15 20 25 30 35 40 45 50 55 60 65 70
Physical position (Mb)

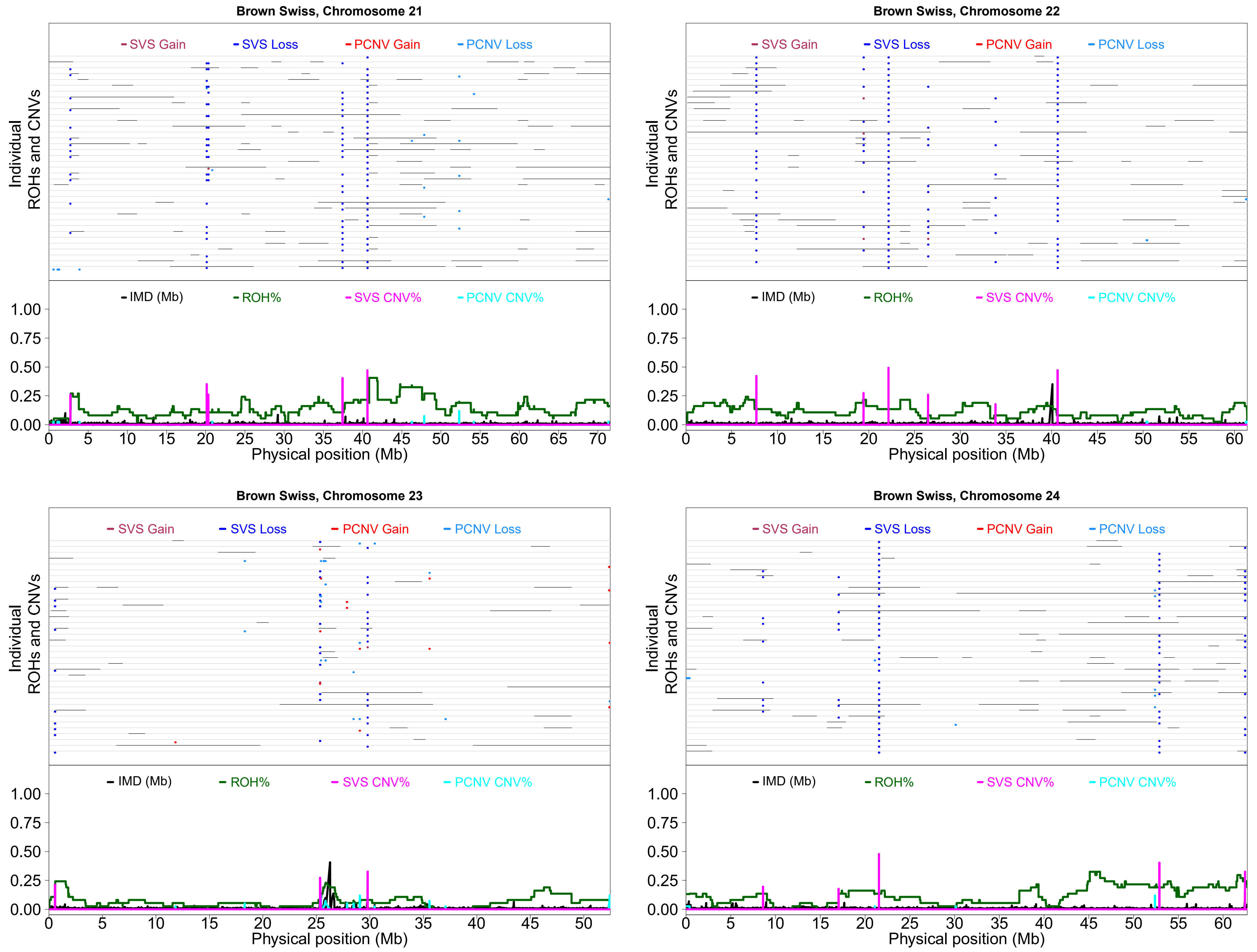

Brown Swiss, Chromosome 21
Brown Swiss, Chromosome 22
Brown Swiss, Chromosome 23
Brown Swiss, Chromosome 24
SVS Gain
SVS Loss
PCNV Gain
PCNV Loss
Individual ROHs and CNVs
IMD (Mb)
ROH%
SVS CNV%
PCNV CNV%
1.00
0.75
0.50
0.25
0.00
Physical position (Mb)

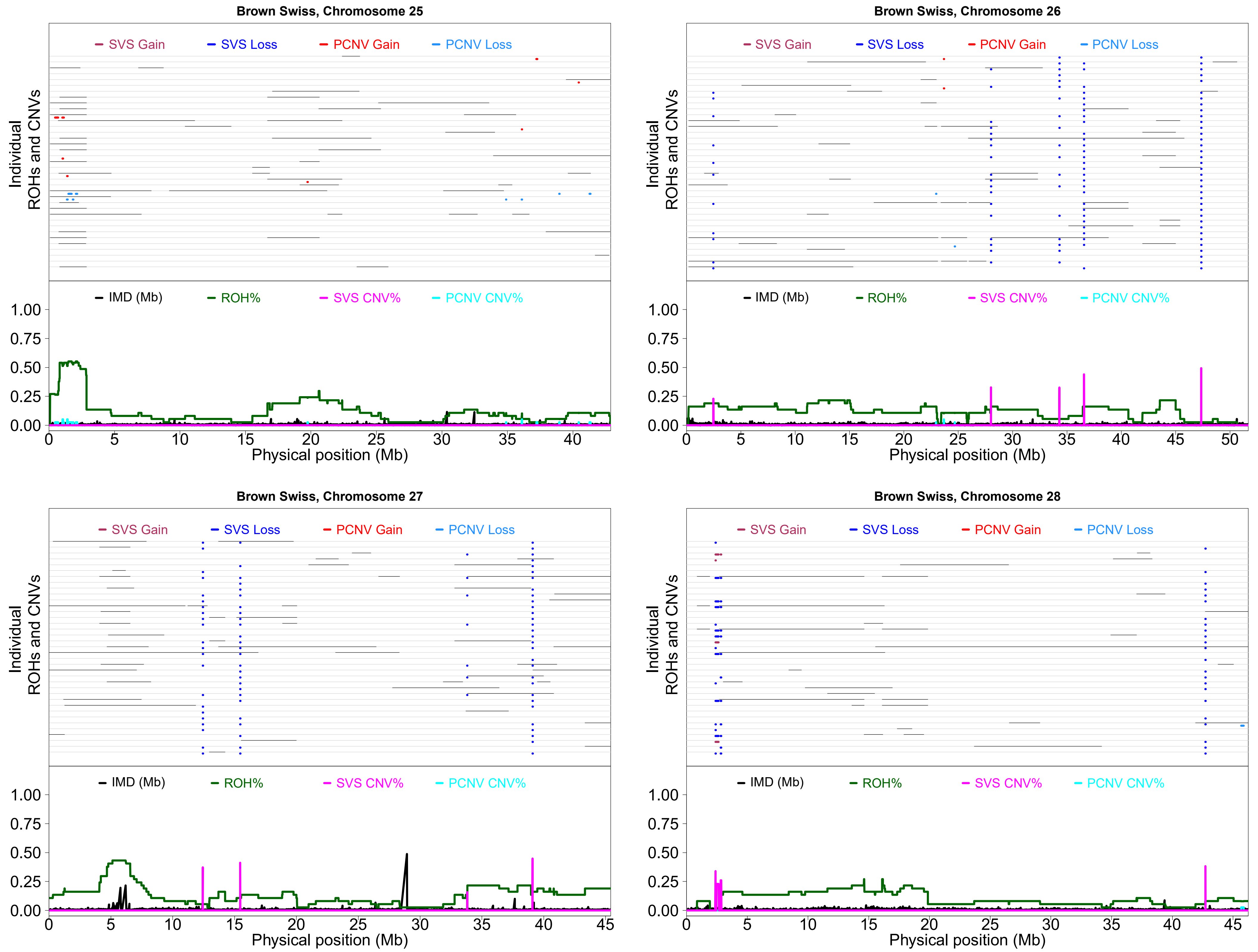
Brown Swiss, Chromosome 25
Brown Swiss, Chromosome 26
Brown Swiss, Chromosome 27
Brown Swiss, Chromosome 28
SVS Gain
SVS Loss
PCNV Gain
PCNV Loss
Individual ROHs and CNVs
IMD (Mb)
ROH%
SVS CNV%
PCNV CNV%
1.00
0.75
0.50
0.25
0.00
Physical position (Mb)

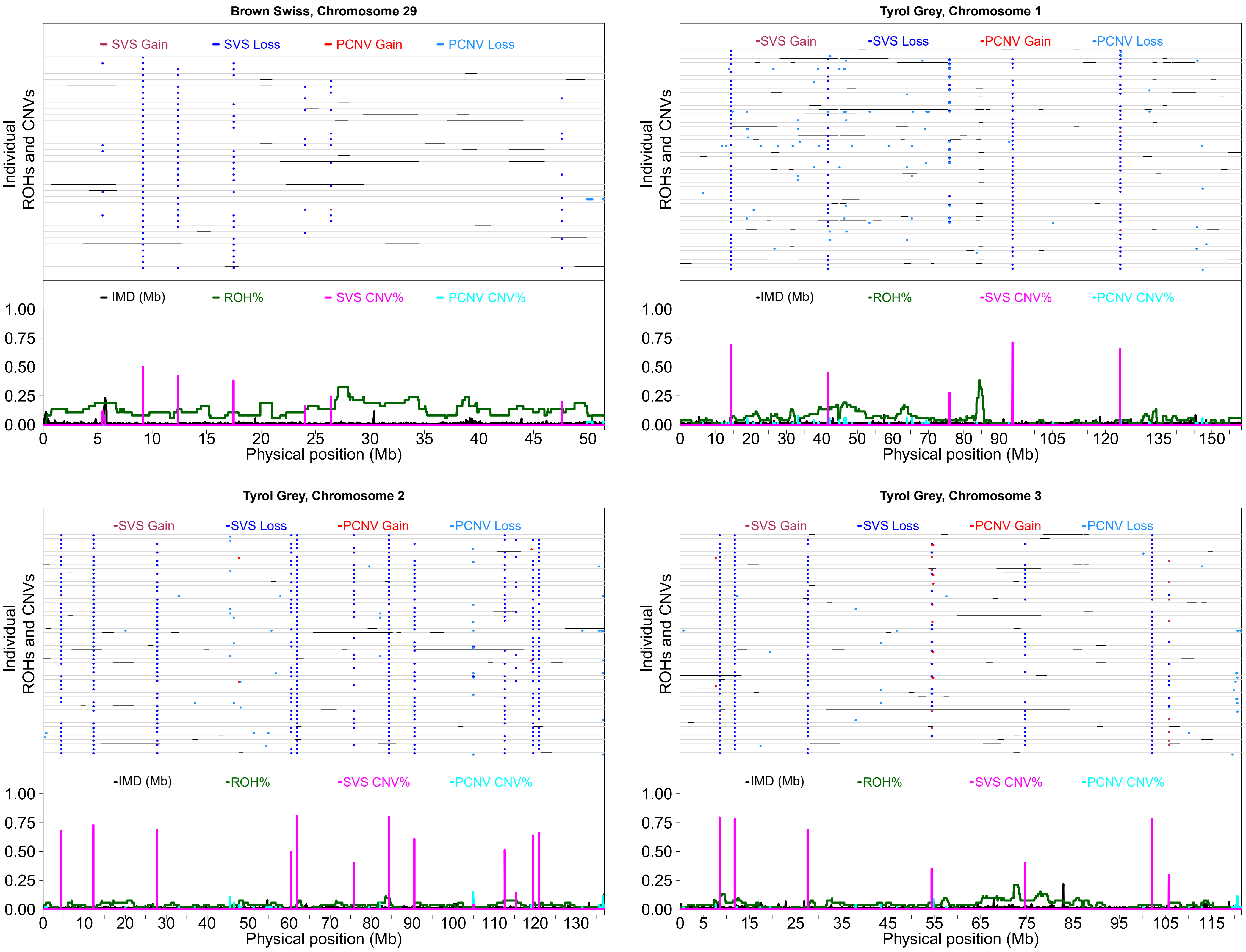

Brown Swiss, Chromosome 29
Tyrol Grey, Chromosome 1
Tyrol Grey, Chromosome 2
Tyrol Grey, Chromosome 3
SVS Gain
SVS Loss
PCNV Gain
PCNV Loss
Individual ROHs and CNVs
IMD (Mb)
ROH%
SVS CNV%
PCNV CNV%
Physical position (Mb)

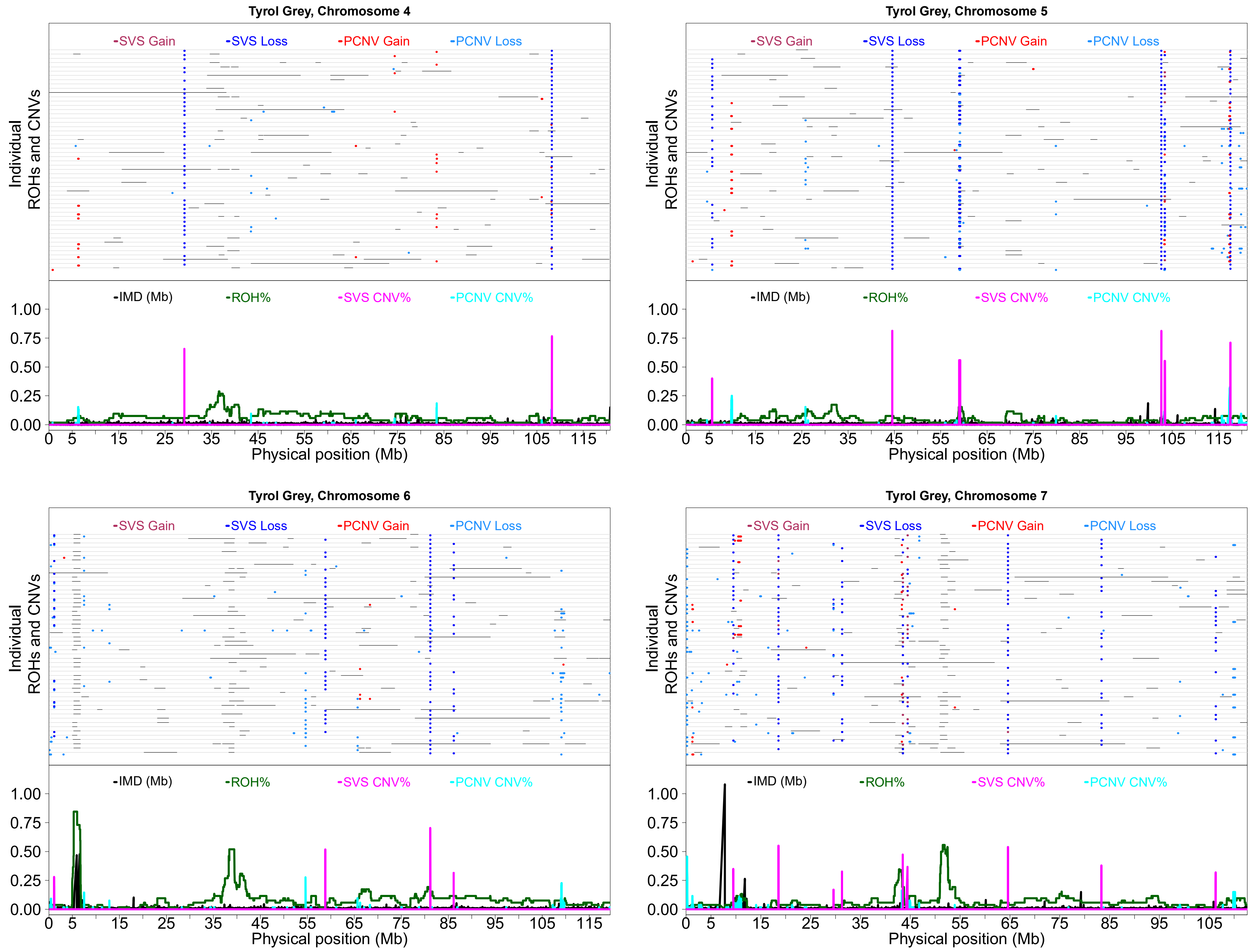
Tyrol Grey, Chromosome 4
Tyrol Grey, Chromosome 5
Tyrol Grey, Chromosome 6
Tyrol Grey, Chromosome 7
SVS Gain
SVS Loss
PCNV Gain
PCNV Loss
Individual ROHs and CNVs
IMD (Mb)
ROH%
SVS CNV%
PCNV CNV%
1.00
0.75
0.50
0.25
0.00
0
5
15
25
35
45
55
65
75
85
95
105
115
Physical position (Mb)

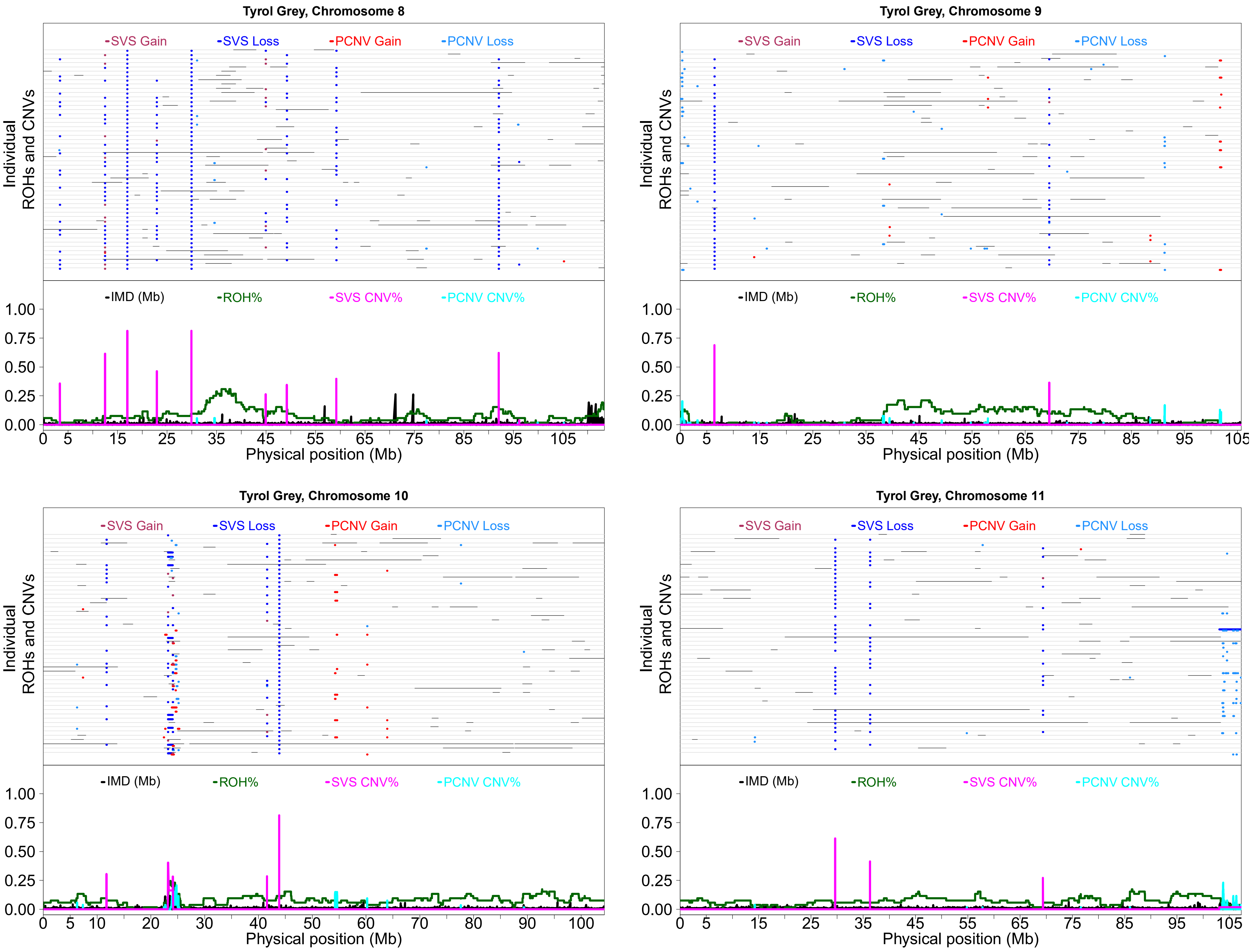

Tyrol Grey, Chromosome 8
Tyrol Grey, Chromosome 9
Tyrol Grey, Chromosome 10
Tyrol Grey, Chromosome 11
SVS Gain
SVS Loss
PCNV Gain
PCNV Loss
IMD (Mb)
ROH%
SVS CNV%
PCNV CNV%
Individual ROHs and CNVs
Physical position (Mb)

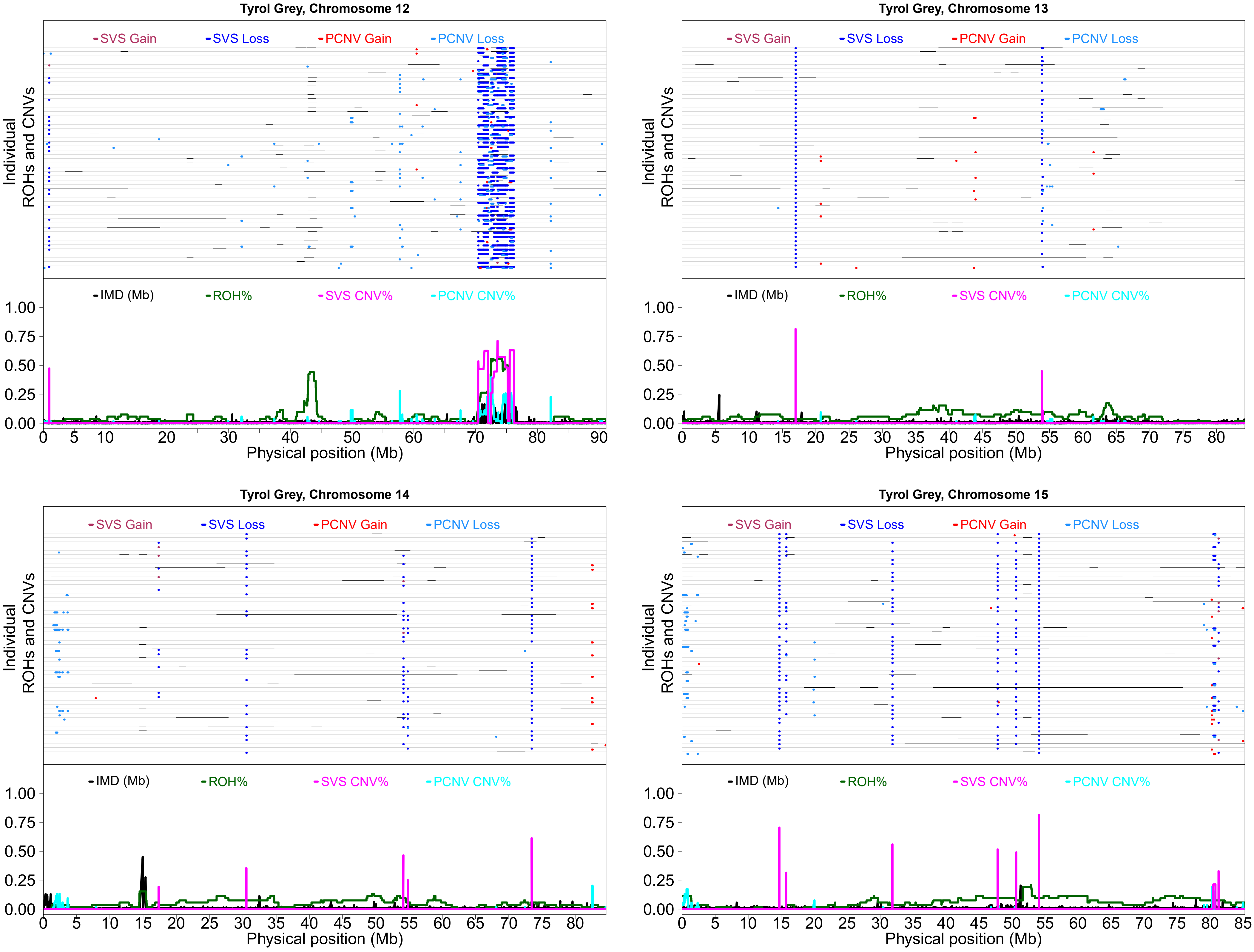

Tyrol Grey, Chromosome 12
Tyrol Grey, Chromosome 13
Tyrol Grey, Chromosome 14
Tyrol Grey, Chromosome 15
SVS Gain
SVS Loss
PCNV Gain
PCNV Loss
Individual ROHs and CNVs
IMD (Mb)
ROH%
SVS CNV%
PCNV CNV%
1.00
0.75
0.50
0.25
0.00
Physical position (Mb)

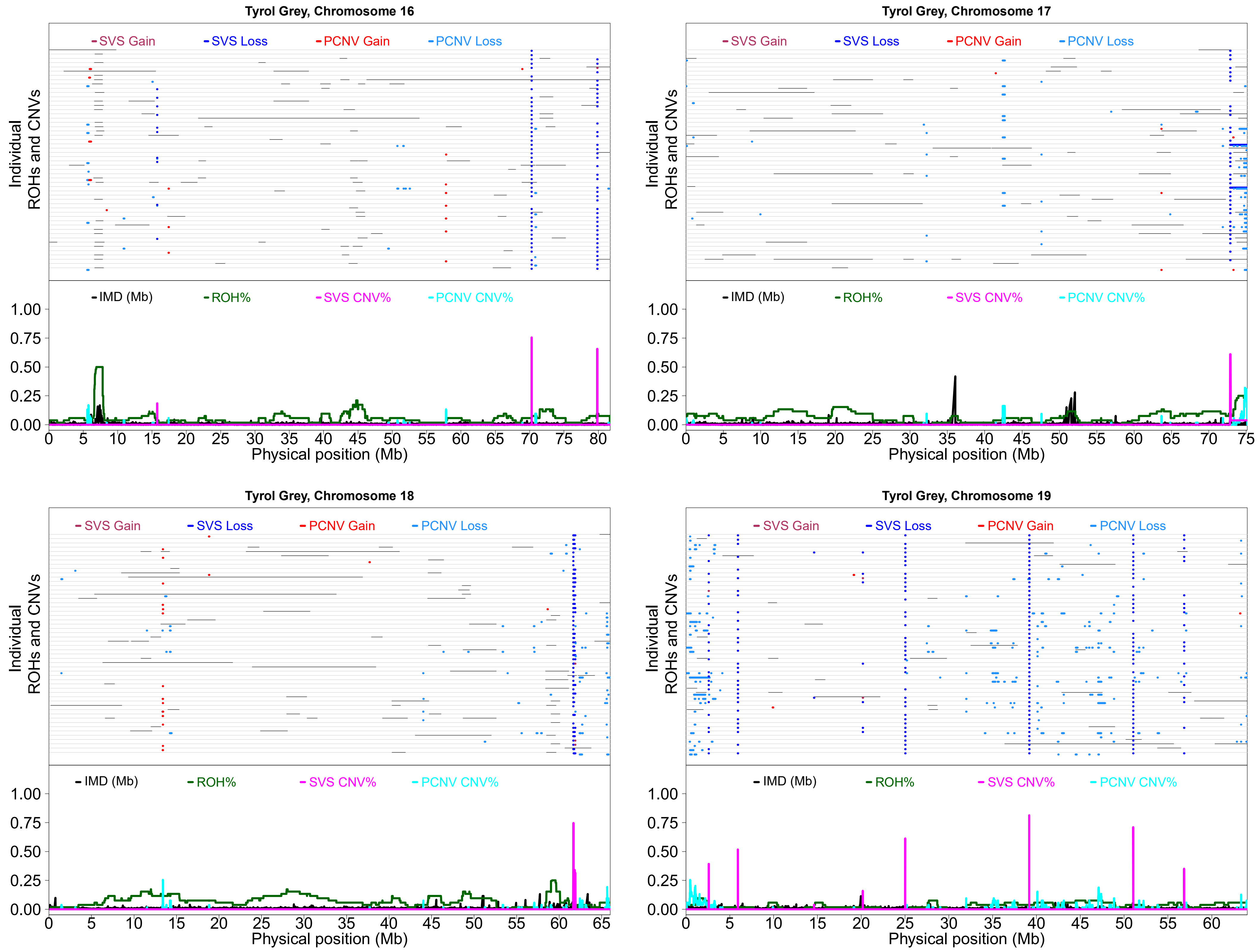
Tyrol Grey, Chromosome 16
Tyrol Grey, Chromosome 17
Tyrol Grey, Chromosome 18
Tyrol Grey, Chromosome 19
SVS Gain
SVS Loss
PCNV Gain
PCNV Loss
Individual ROHs and CNVs
IMD (Mb)
ROH%
SVS CNV%
PCNV CNV%
1.00
0.75
0.50
0.25
0.00
Physical position (Mb)

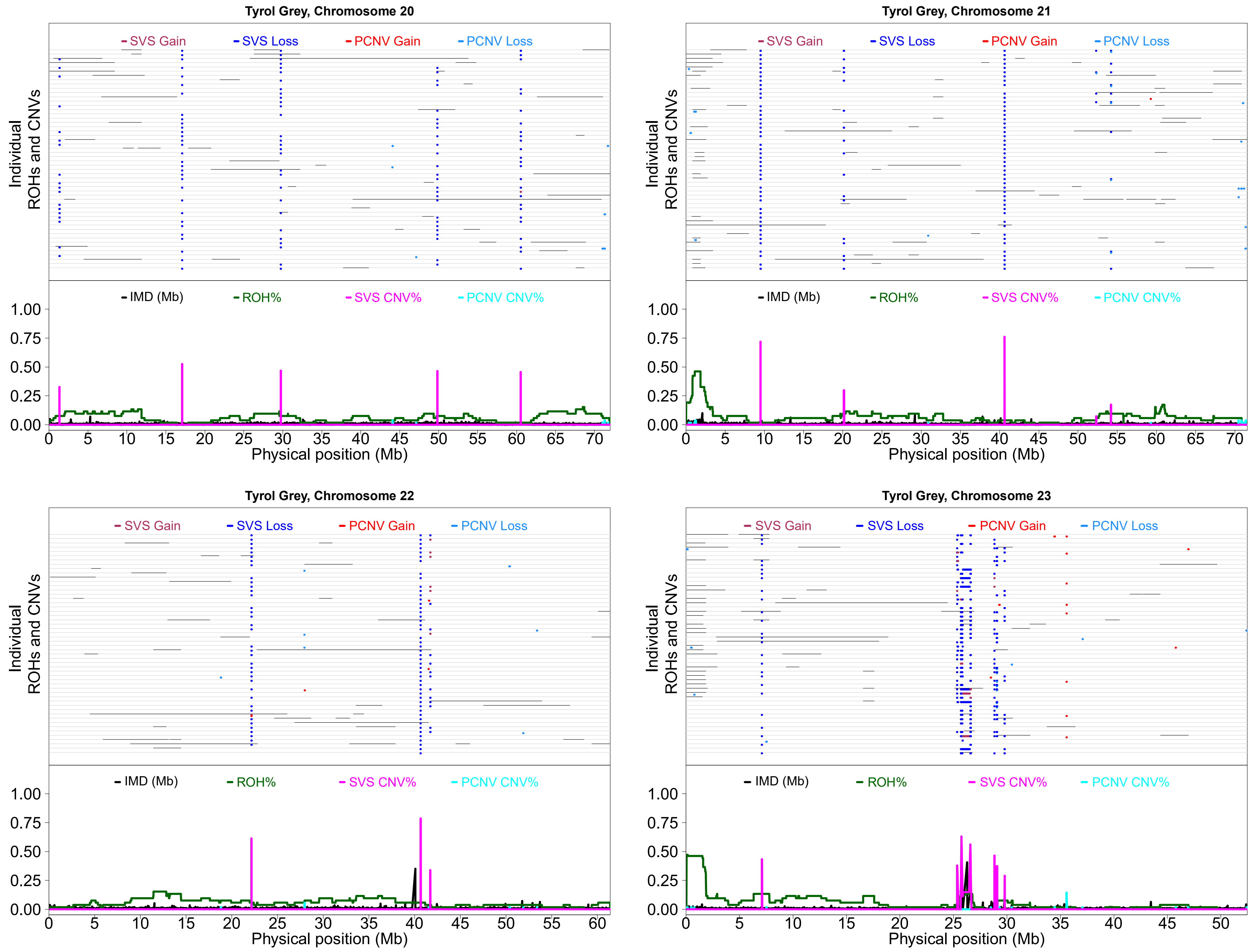
Tyrol Grey, Chromosome 20
Tyrol Grey, Chromosome 21
Tyrol Grey, Chromosome 22
Tyrol Grey, Chromosome 23
SVS Gain
SVS Loss
PCNV Gain
PCNV Loss
Individual ROHs and CNVs
IMD (Mb)
ROH%
SVS CNV%
PCNV CNV%
1.00
0.75
0.50
0.25
0.00
Physical position (Mb)

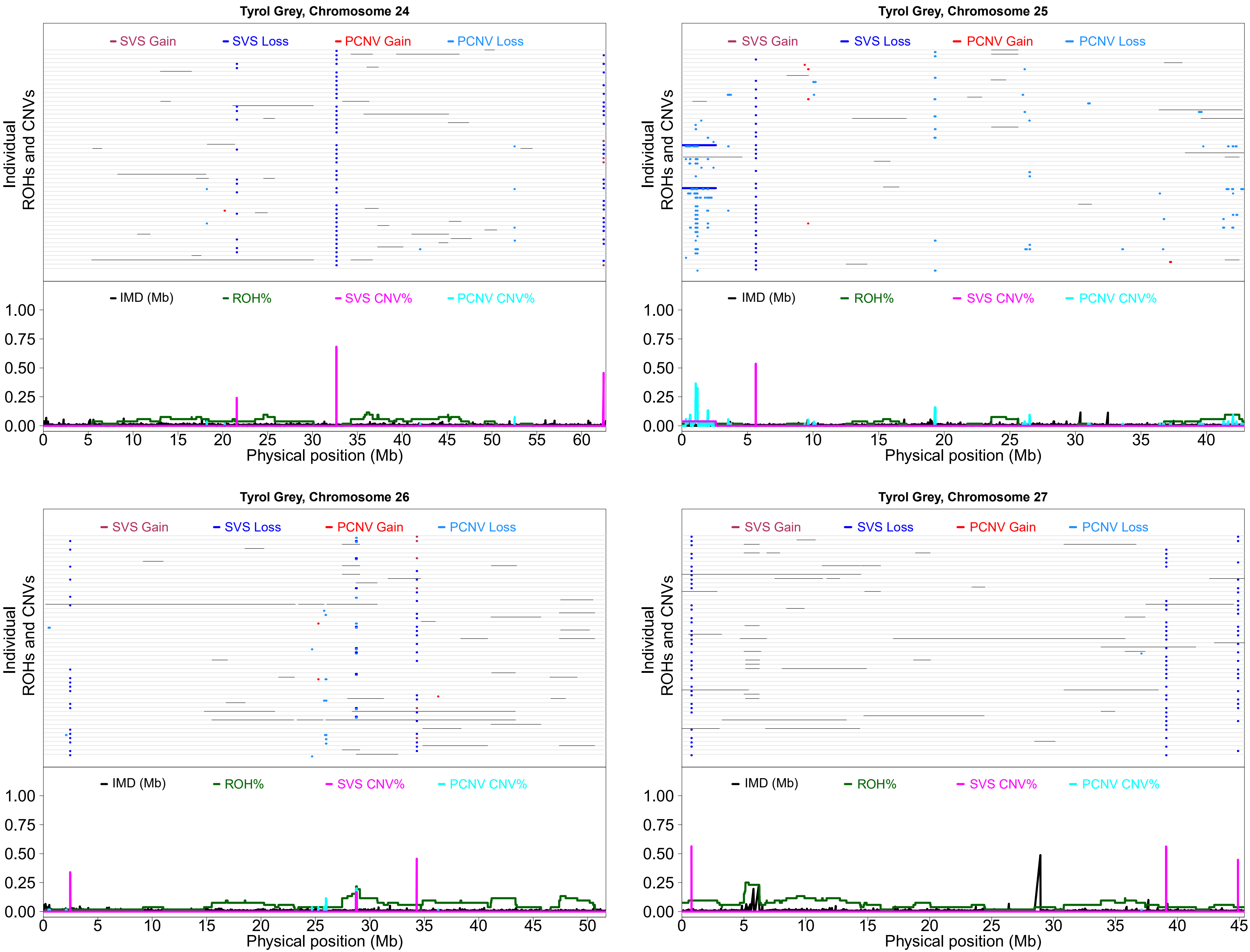

Tyrol Grey, Chromosome 24
SVS Gain
SVS Loss
PCNV Gain
PCNV Loss
Individual ROHs and CNVs
IMD (Mb)
ROH%
SVS CNV%
PCNV CNV%
1.00
0.75
0.50
0.25
0.00
0
5
10
15
20
25
30
35
40
45
50
55
60
Physical position (Mb)
Tyrol Grey, Chromosome 25
SVS Gain
SVS Loss
PCNV Gain
PCNV Loss
Individual ROHs and CNVs
IMD (Mb)
ROH%
SVS CNV%
PCNV CNV%
1.00
0.75
0.50
0.25
0.00
0
5
10
15
20
25
30
35
40
Physical position (Mb)
Tyrol Grey, Chromosome 26
SVS Gain
SVS Loss
PCNV Gain
PCNV Loss
Individual ROHs and CNVs
IMD (Mb)
ROH%
SVS CNV%
PCNV CNV%
1.00
0.75
0.50
0.25
0.00
0
5
10
15
20
25
30
35
40
45
50
Physical position (Mb)
Tyrol Grey, Chromosome 27
SVS Gain
SVS Loss
PCNV Gain
PCNV Loss
Individual ROHs and CNVs
IMD (Mb)
ROH%
SVS CNV%
PCNV CNV%
1.00
0.75
0.50
0.25
0.00
0
5
10
15
20
25
30
35
40
45
Physical position (Mb)

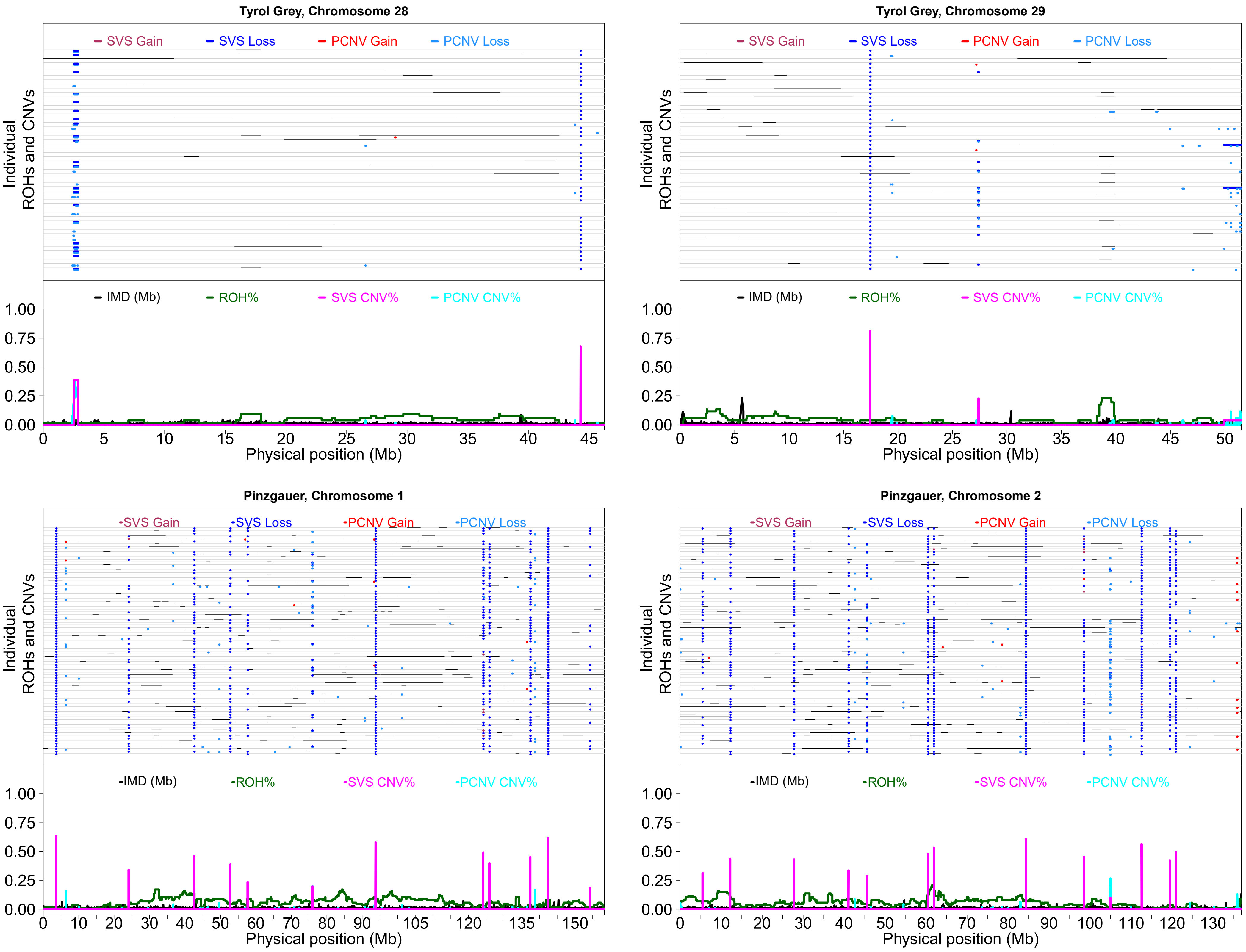

Tyrol Grey, Chromosome 28
Tyrol Grey, Chromosome 29
Pinzgauer, Chromosome 1
Pinzgauer, Chromosome 2
SVS Gain
SVS Loss
PCNV Gain
PCNV Loss
Individual ROHs and CNVs
IMD (Mb)
ROH%
SVS CNV%
PCNV CNV%
Physical position (Mb)

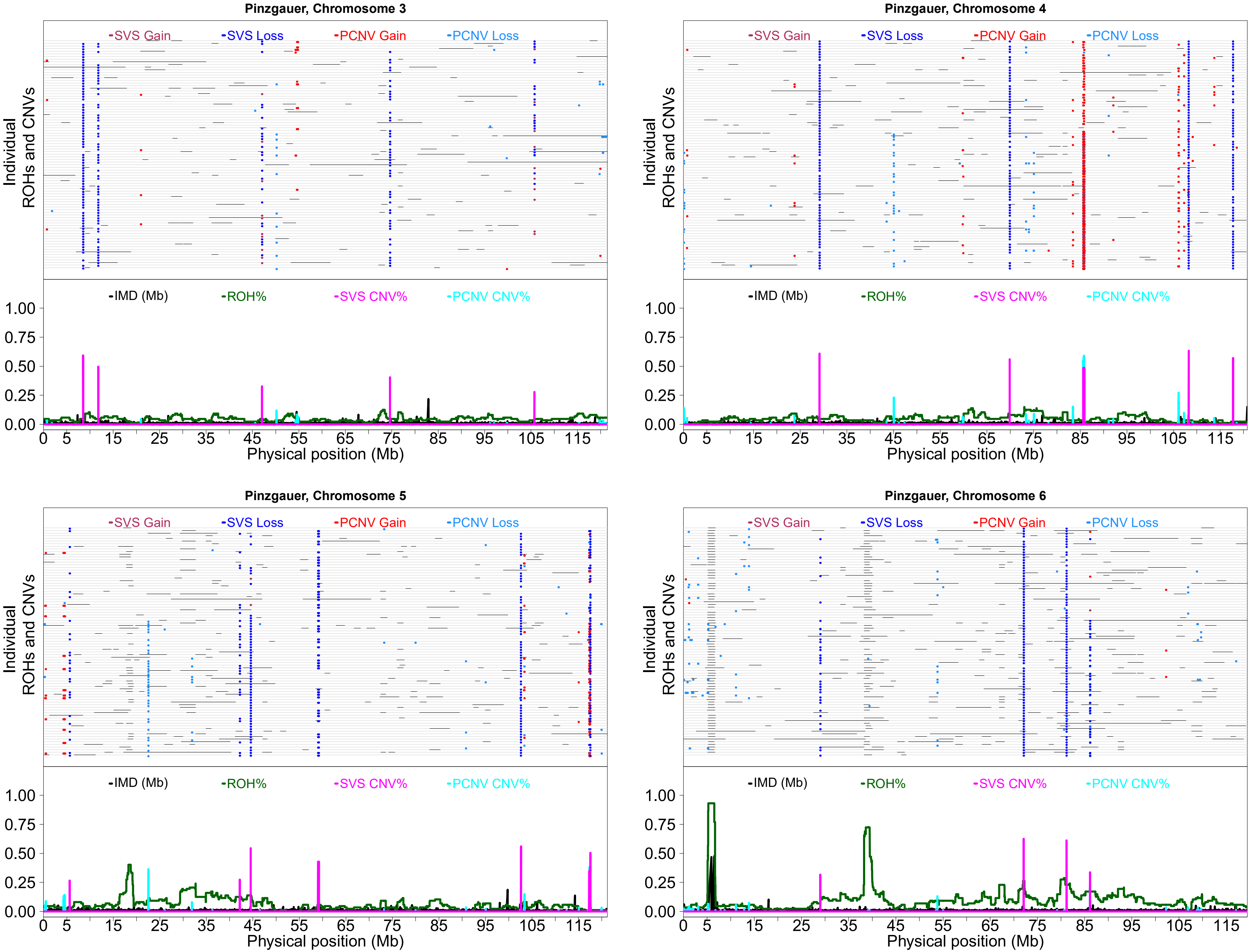

Pinzgauer, Chromosome 3
Pinzgauer, Chromosome 4
Pinzgauer, Chromosome 5
Pinzgauer, Chromosome 6
SVS Gain
SVS Loss
PCNV Gain
PCNV Loss
Individual ROHs and CNVs
IMD (Mb)
ROH%
SVS CNV%
PCNV CNV%
1.00
0.75
0.50
0.25
0.00
0 5 15 25 35 45 55 65 75 85 95 105 115
Physical position (Mb)

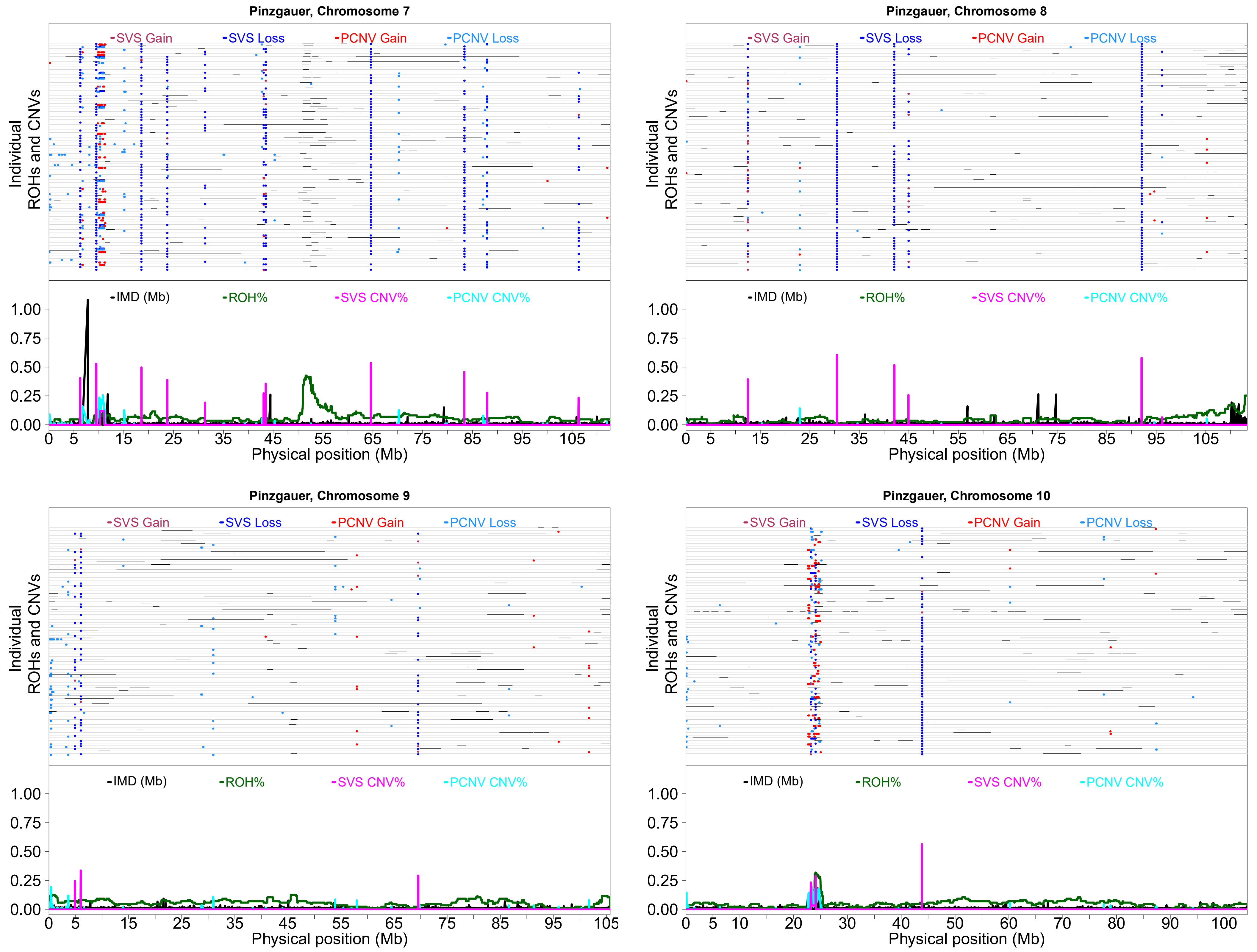

Pinzgauer, Chromosome 7
Pinzgauer, Chromosome 8
Pinzgauer, Chromosome 9
Pinzgauer, Chromosome 10
SVS Gain
SVS Loss
PCNV Gain
PCNV Loss
Individual ROHs and CNVs
IMD (Mb)
ROH%
SVS CNV%
PCNV CNV%
1.00
0.75
0.50
0.25
0.00
Physical position (Mb)

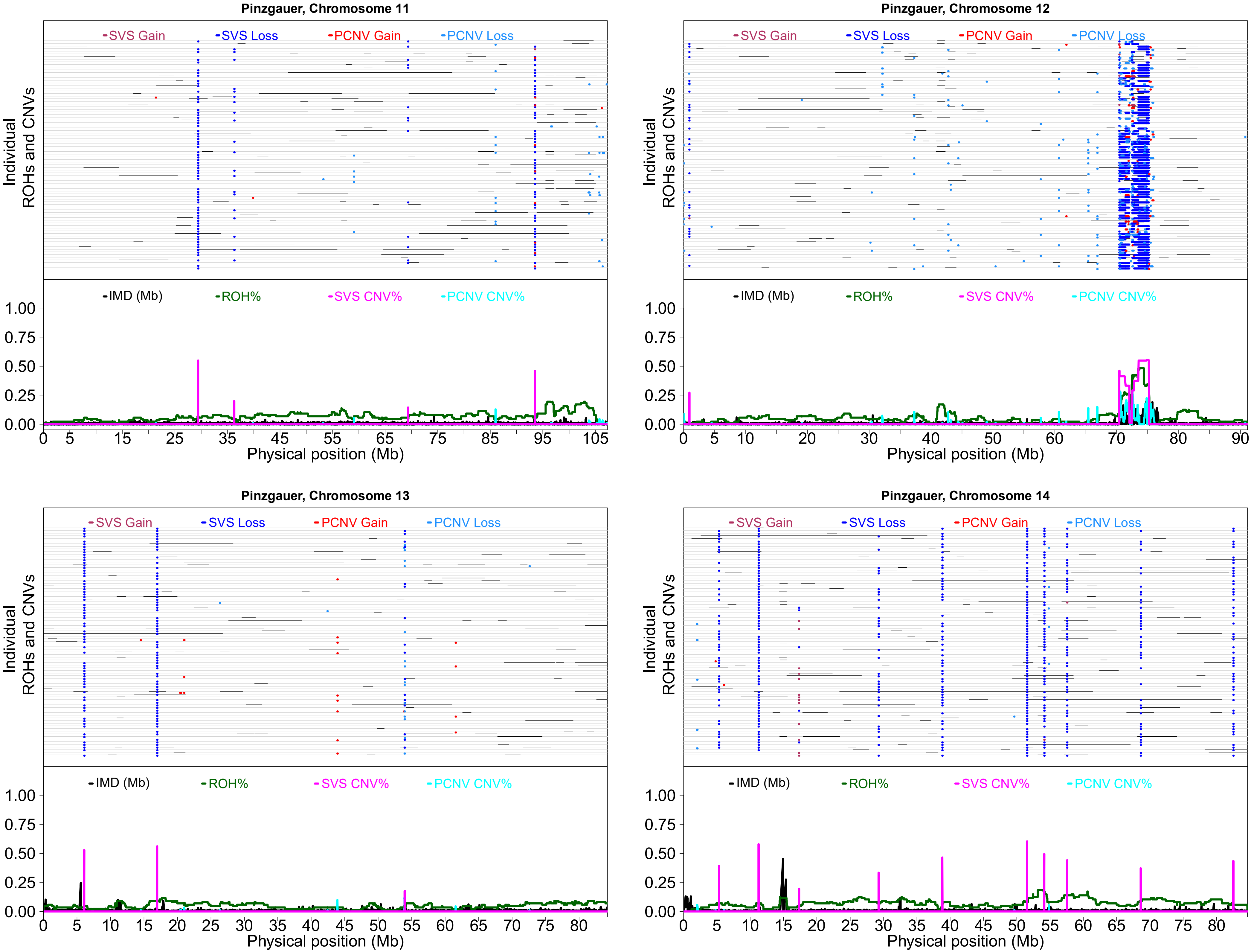

Pinzgauer, Chromosome 11
SVS Gain
SVS Loss
PCNV Gain
PCNV Loss
Individual ROHs and CNVs
IMD (Mb)
ROH%
SVS CNV%
PCNV CNV%
1.00
0.75
0.50
0.25
0.00
0 5 15 25 35 45 55 65 75 85 95 105
Physical position (Mb)
Pinzgauer, Chromosome 12
SVS Gain
SVS Loss
PCNV Gain
PCNV Loss
Individual ROHs and CNVs
IMD (Mb)
ROH%
SVS CNV%
PCNV CNV%
1.00
0.75
0.50
0.25
0.00
0 5 10 20 30 40 50 60 70 80 90
Physical position (Mb)
Pinzgauer, Chromosome 13
SVS Gain
SVS Loss
PCNV Gain
PCNV Loss
Individual ROHs and CNVs
IMD (Mb)
ROH%
SVS CNV%
PCNV CNV%
1.00
0.75
0.50
0.25
0.00
0 5 10 15 20 25 30 35 40 45 50 55 60 65 70 75 80
Physical position (Mb)
Pinzgauer, Chromosome 14
SVS Gain
SVS Loss
PCNV Gain
PCNV Loss
Individual ROHs and CNVs
IMD (Mb)
ROH%
SVS CNV%
PCNV CNV%
1.00
0.75
0.50
0.25
0.00
0 5 10 15 20 25 30 35 40 45 50 55 60 65 70 75 80
Physical position (Mb)

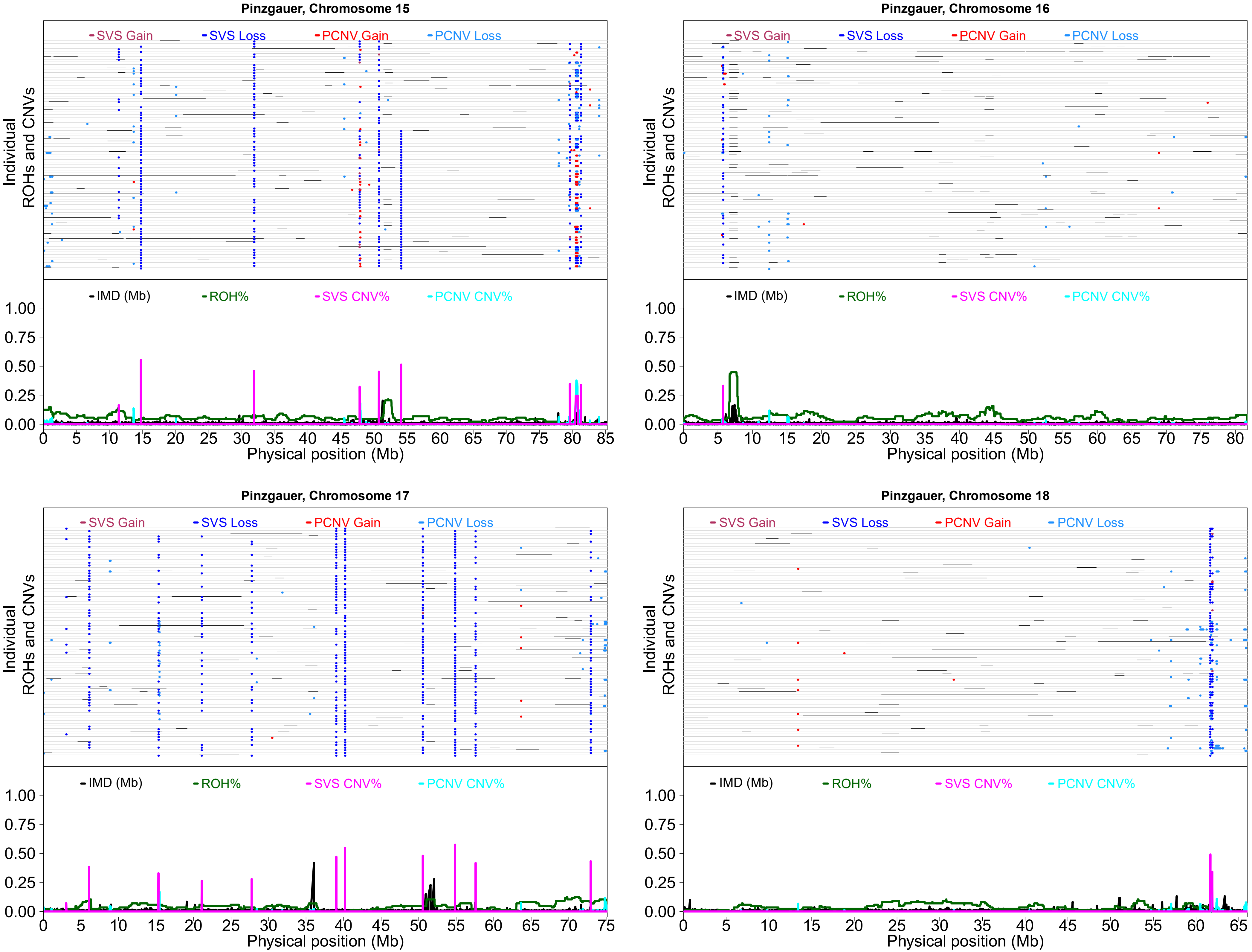
Pinzgauer, Chromosome 15
SVS Gain
SVS Loss
PCNV Gain
PCNV Loss
Individual ROHs and CNVs
IMD (Mb)
ROH%
SVS CNV%
PCNV CNV%
1.00
0.75
0.50
0.25
0.00
0 5 10 15 20 25 30 35 40 45 50 55 60 65 70 75 80 85
Physical position (Mb)
Pinzgauer, Chromosome 16
0 5 10 15 20 25 30 35 40 45 50 55 60 65 70 75 80
Pinzgauer, Chromosome 17
0 5 10 15 20 25 30 35 40 45 50 55 60 65 70 75
Pinzgauer, Chromosome 18
0 5 10 15 20 25 30 35 40 45 50 55 60 65

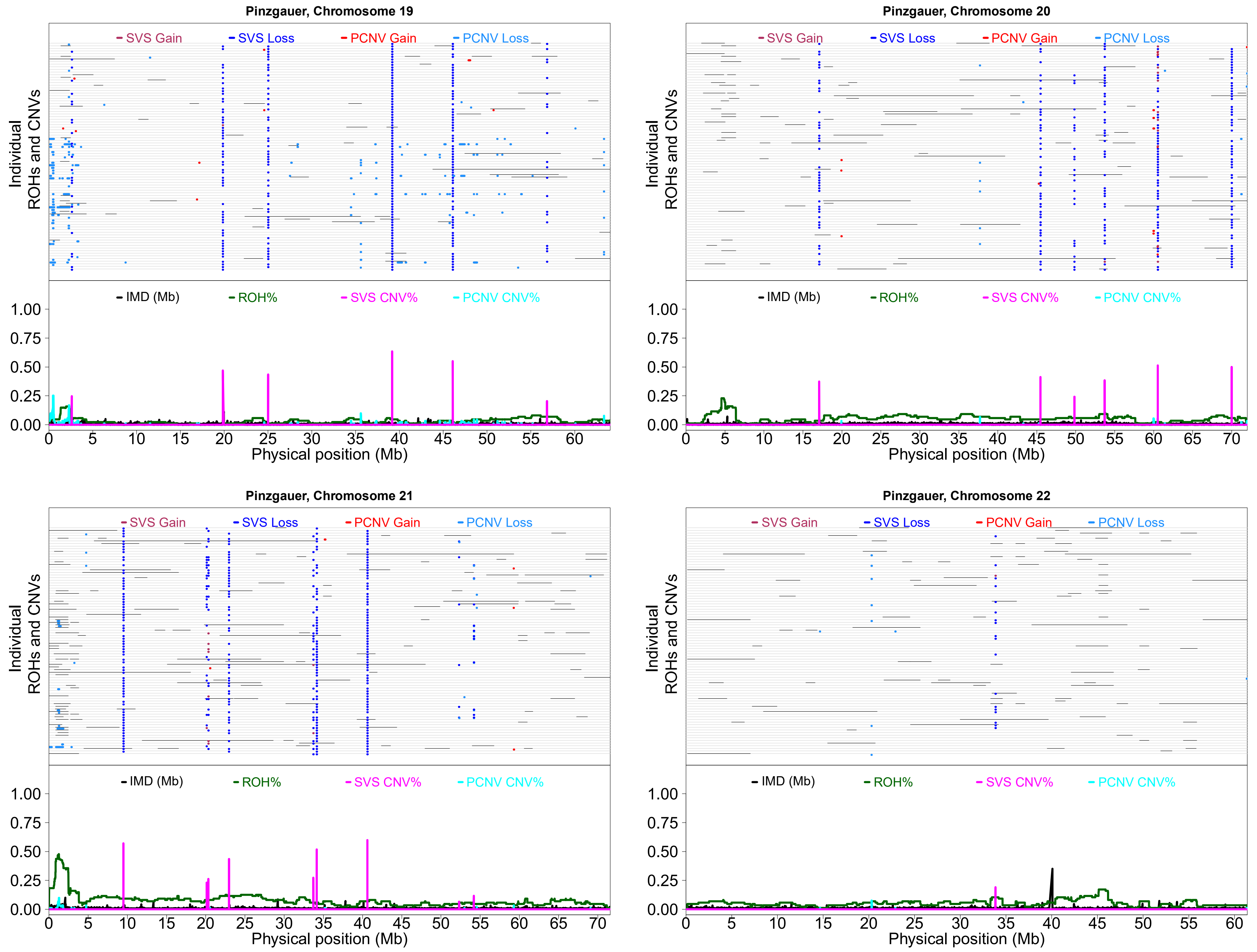

Pinzgauer, Chromosome 19
SVS Gain
SVS Loss
PCNV Gain
PCNV Loss
Individual ROHs and CNVs
IMD (Mb)
ROH%
SVS CNV%
PCNV CNV%
1.00
0.75
0.50
0.25
0.00
0 5 10 15 20 25 30 35 40 45 50 55 60
Physical position (Mb)
Pinzgauer, Chromosome 20
SVS Gain
SVS Loss
PCNV Gain
PCNV Loss
Individual ROHs and CNVs
IMD (Mb)
ROH%
SVS CNV%
PCNV CNV%
1.00
0.75
0.50
0.25
0.00
0 5 10 15 20 25 30 35 40 45 50 55 60 65 70
Physical position (Mb)
Pinzgauer, Chromosome 21
SVS Gain
SVS Loss
PCNV Gain
PCNV Loss
Individual ROHs and CNVs
IMD (Mb)
ROH%
SVS CNV%
PCNV CNV%
1.00
0.75
0.50
0.25
0.00
0 5 10 15 20 25 30 35 40 45 50 55 60 65 70
Physical position (Mb)
Pinzgauer, Chromosome 22
SVS Gain
SVS Loss
PCNV Gain
PCNV Loss
Individual ROHs and CNVs
IMD (Mb)
ROH%
SVS CNV%
PCNV CNV%
1.00
0.75
0.50
0.25
0.00
0 5 10 15 20 25 30 35 40 45 50 55 60
Physical position (Mb)

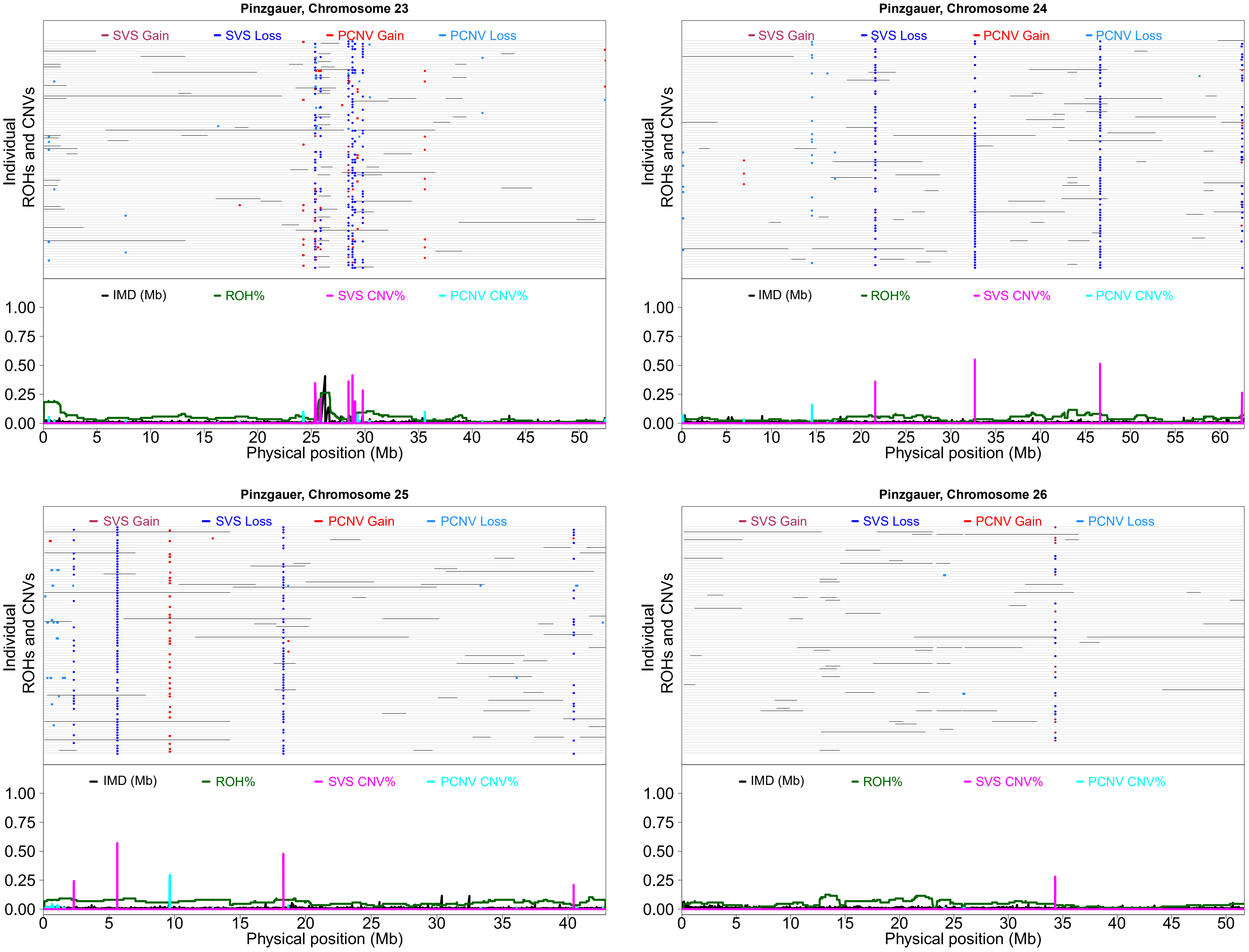
Pinzgauer, Chromosome 23
Pinzgauer, Chromosome 24
Pinzgauer, Chromosome 25
Pinzgauer, Chromosome 26
SVS Gain
SVS Loss
PCNV Gain
PCNV Loss
Individual ROHs and CNVs
IMD (Mb)
ROH%
SVS CNV%
PCNV CNV%
1.00
0.75
0.50
0.25
0.00
Physical position (Mb)

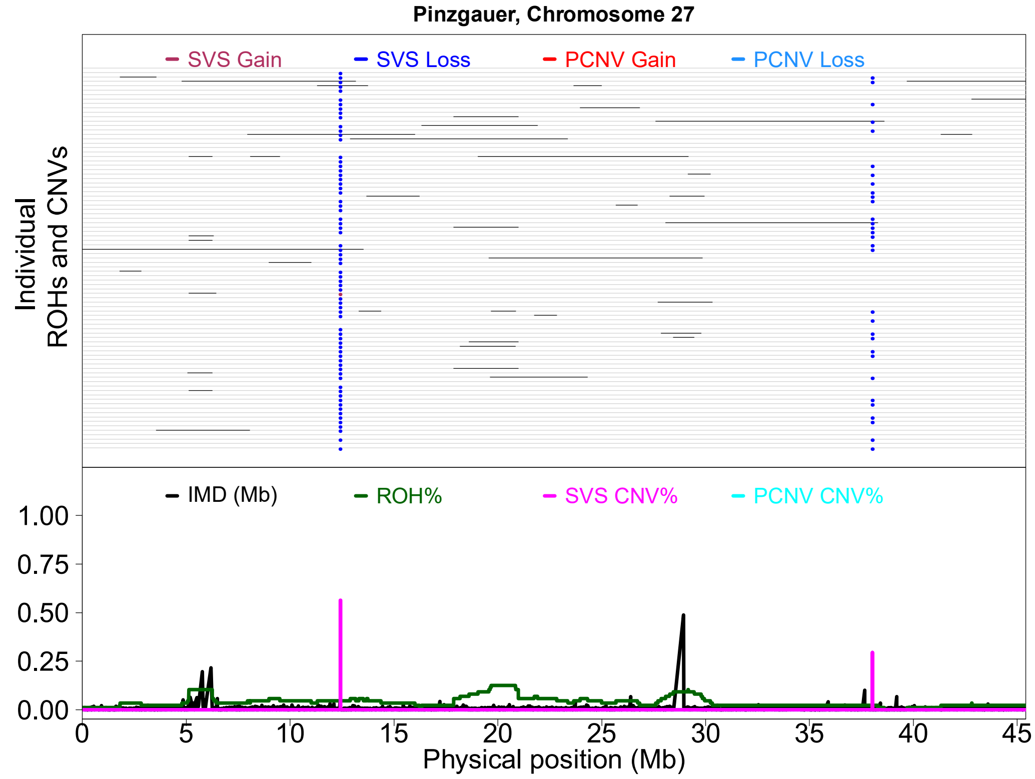
Pinzgauer, Chromosome 27
SVS Gain
SVS Loss
PCNV Gain
PCNV Loss
Individual ROHs and CNVs
IMD (Mb)
ROH%
SVS CNV%
PCNV CNV%
1.00
0.75
0.50
0.25
0.00
0
5
10
15
20
25
30
35
40
45
Physical position (Mb)

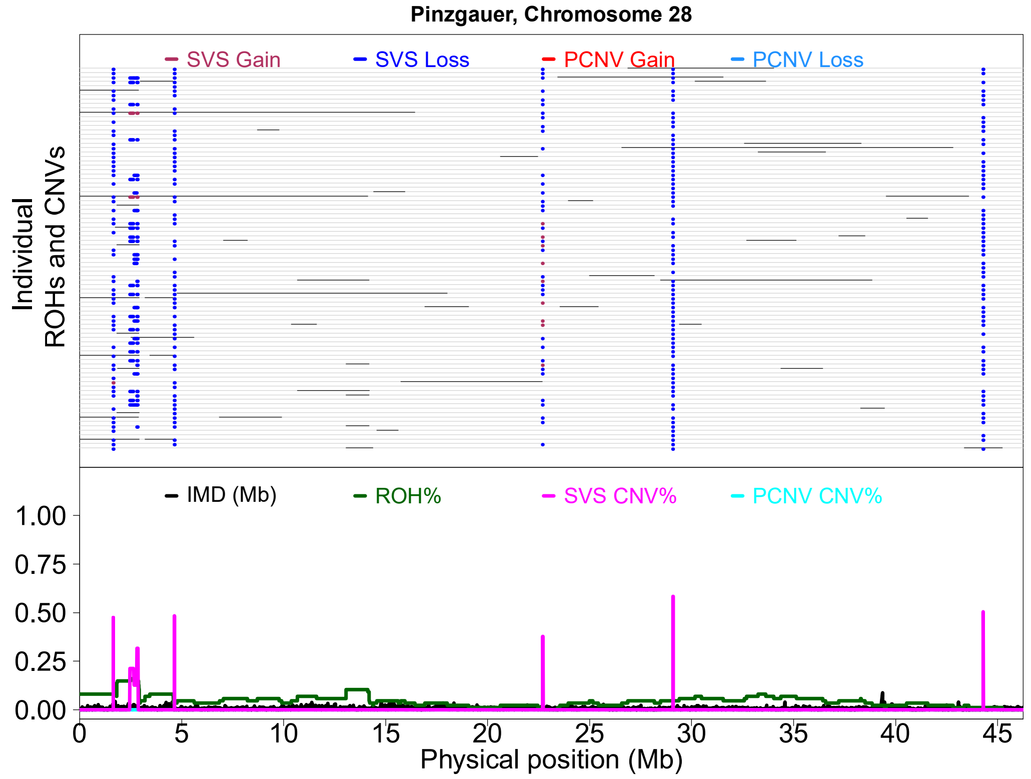
Pinzgauer, Chromosome 28
SVS Gain
SVS Loss
PCNV Gain
PCNV Loss
Individual ROHs and CNVs
IMD (Mb)
ROH%
SVS CNV%
PCNV CNV%
1.00
0.75
0.50
0.25
0.00
0
5
10
15
20
25
30
35
40
45
Physical position (Mb)

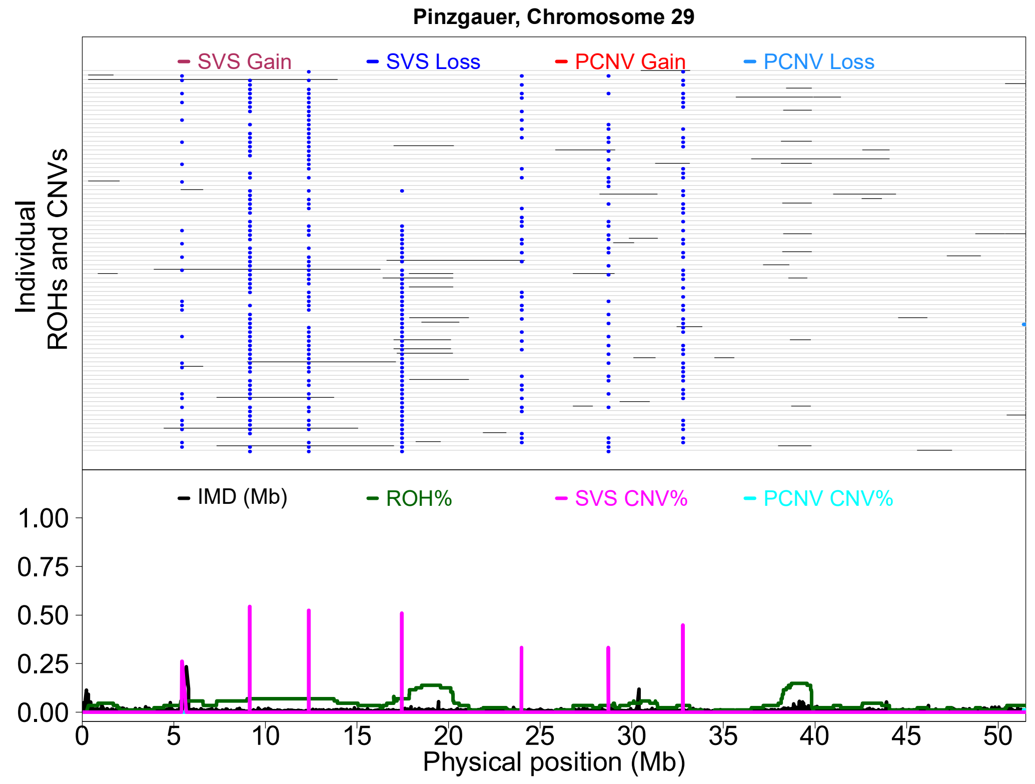
Pinzgauer, Chromosome 29
SVS Gain
SVS Loss
PCNV Gain
PCNV Loss
Individual ROHs and CNVs
IMD (Mb)
ROH%
SVS CNV%
PCNV CNV%
1.00
0.75
0.50
0.25
0.00
0
5
10
15
20
25
30
35
40
45
50
Physical position (Mb)
