# Supplementary material for: Misidentification of runs of homozygosity islands in cattle caused by interference with copy number variation or large intermarker distances
Source: Genet Sel Evol. 2018 Aug 22;50:43. doi: 10.1186/s12711-018-0414-x (PMC6106898; doi:10.1186/s12711-018-0414-x)
Supplement: Supplementary file 5 — Additional file 5: Figure S4. BAF and LRR plot for each of the 57 ROH islands. In each sub-plot, the top panel is for individual ROH (black) and individual CNV (blue and dark red for copy loss and copy gain, respectively, for SVS and light blue and red for copy loss and copy gain, respectively, for PennCNV). The middle panel shows the mean BAF values at each marker while the third panel is for mean LRR at each marker. [file 12711_2018_414_MOESM5_ESM.pdf]

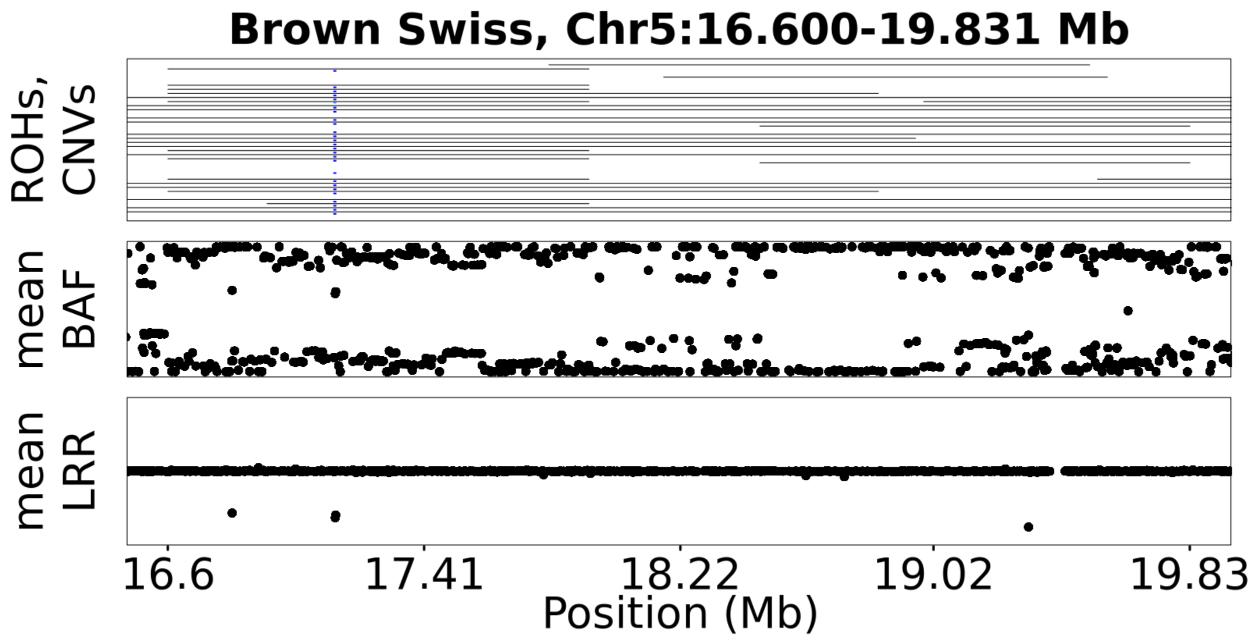
Brown Swiss, Chr5:16.600-19.831 Mb
ROHs, CNVs
mean BAF
mean LRR
16.6
17.41
18.22
19.02
19.83
Position (Mb)

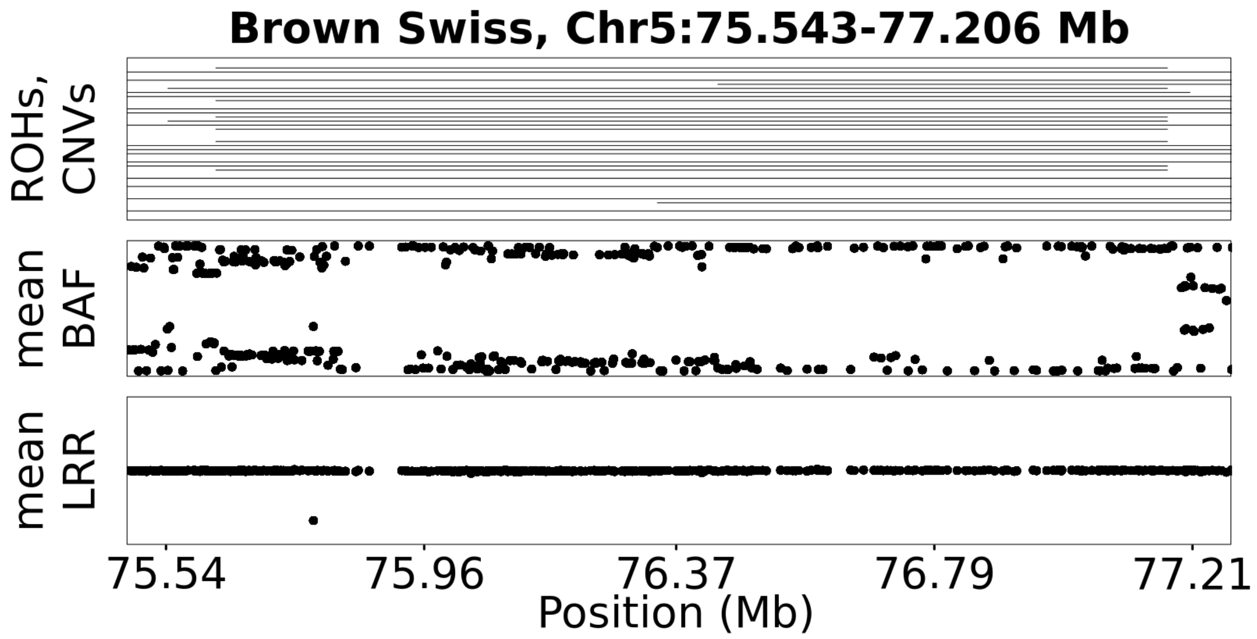
Brown Swiss, Chr5:75.543-77.206 Mb
ROHs, CNVs
mean BAF
mean LRR
75.54
75.96
76.37
76.79
77.21
Position (Mb)

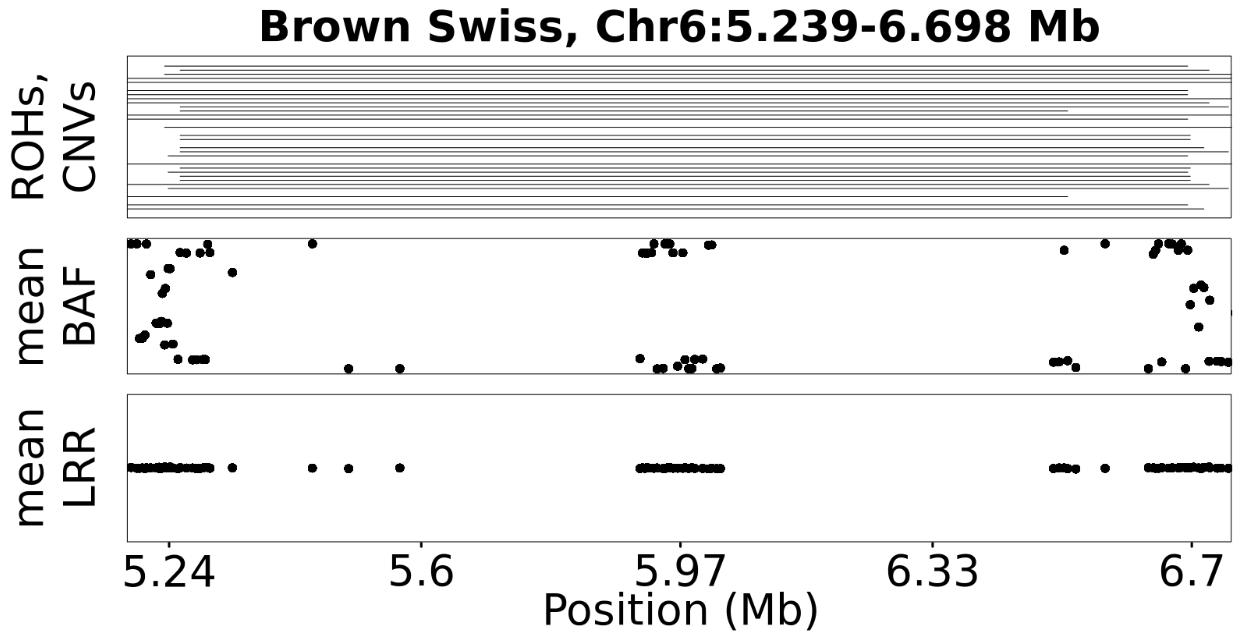
Brown Swiss, Chr6:5.239-6.698 Mb
ROHs, CNVs
mean BAF
mean LRR
5.24
5.6
5.97
6.33
6.7
Position (Mb)

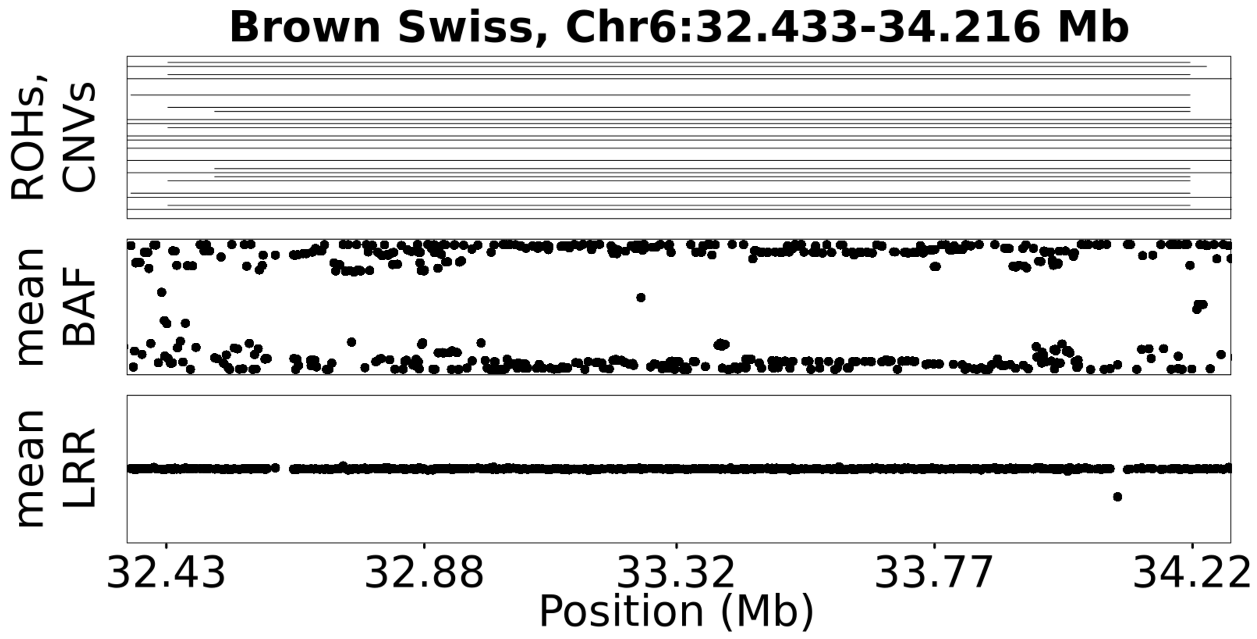
Brown Swiss, Chr6:32.433-34.216 Mb
ROHs, CNVs
mean BAF
mean LRR
32.43
32.88
33.32
33.77
34.22
Position (Mb)

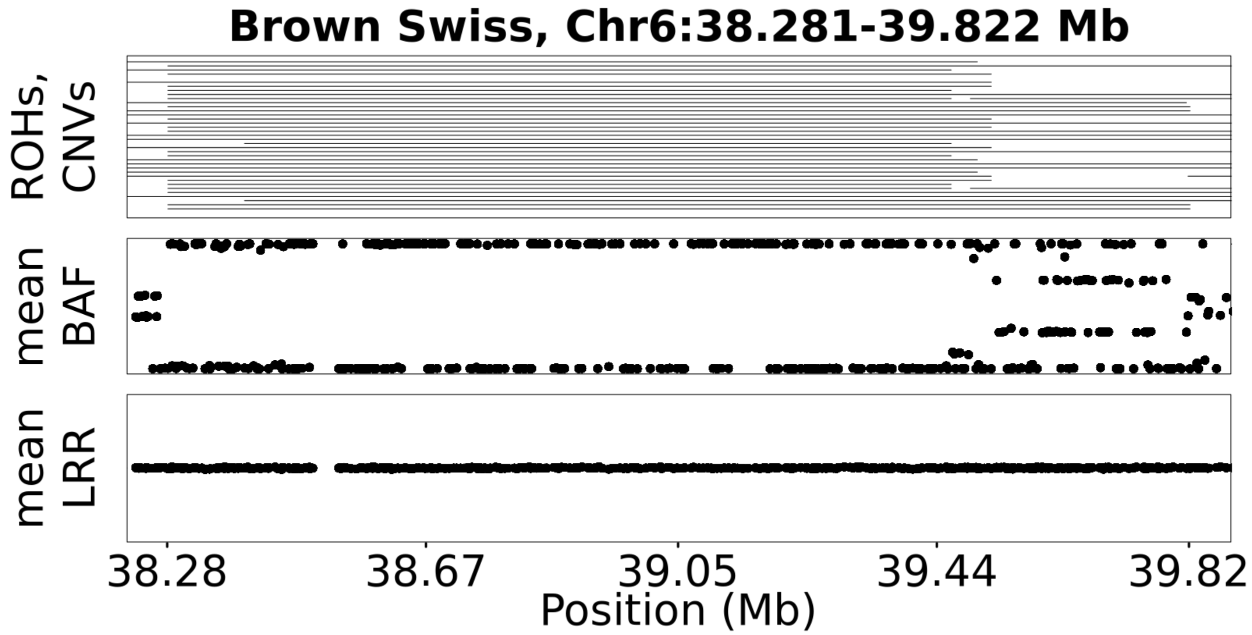
Brown Swiss, Chr6:38.281-39.822 Mb
ROHs, CNVs
mean BAF
mean LRR
38.28
38.67
39.05
39.44
39.82
Position (Mb)

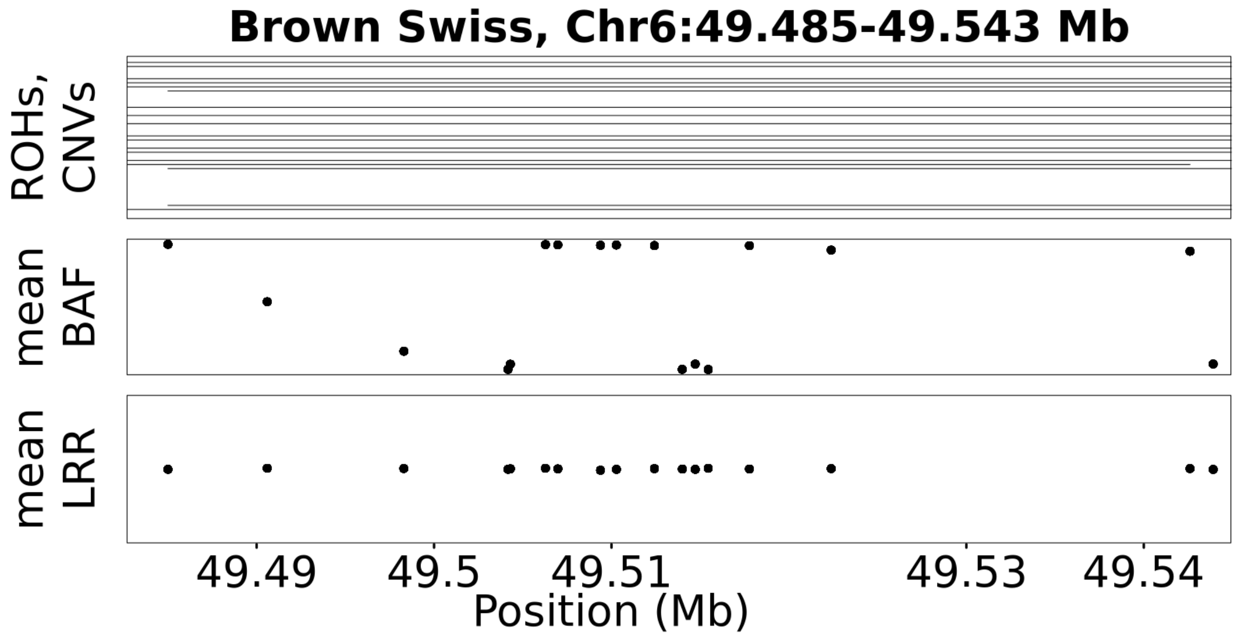
Brown Swiss, Chr6:49.485-49.543 Mb
ROHs, CNVs
mean BAF
mean LRR
49.49
49.5
49.51
49.53
49.54
Position (Mb)

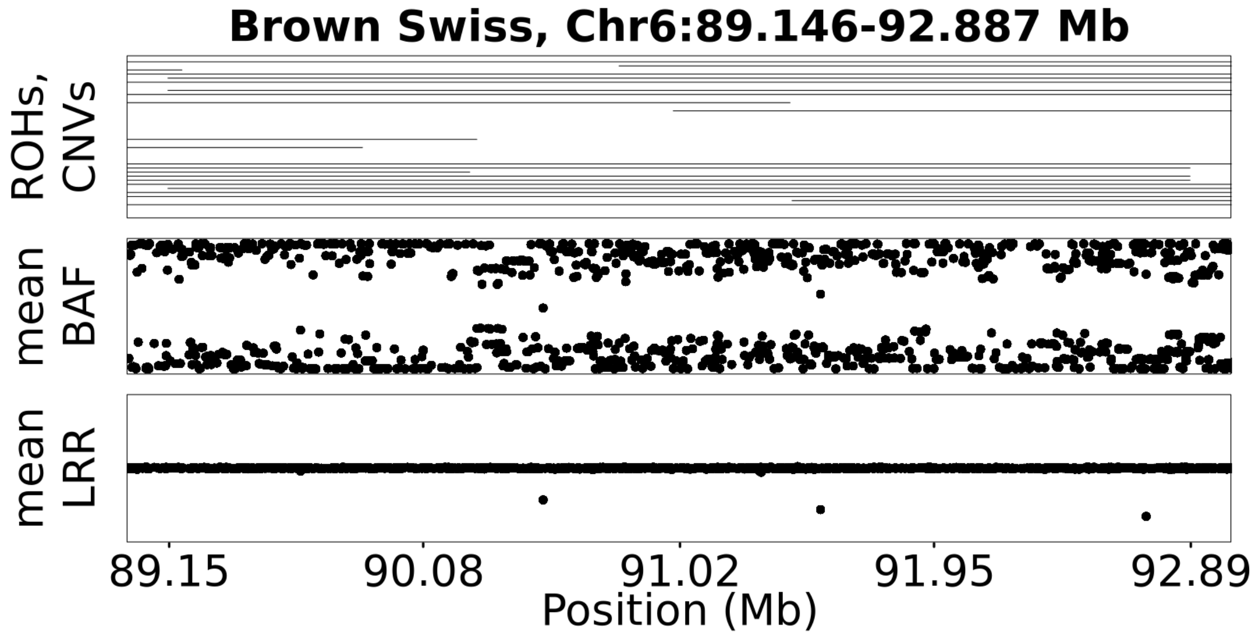
Brown Swiss, Chr6:89.146-92.887 Mb
ROHs, CNVs
mean BAF
mean LRR
89.15
90.08
91.02
91.95
92.89
Position (Mb)

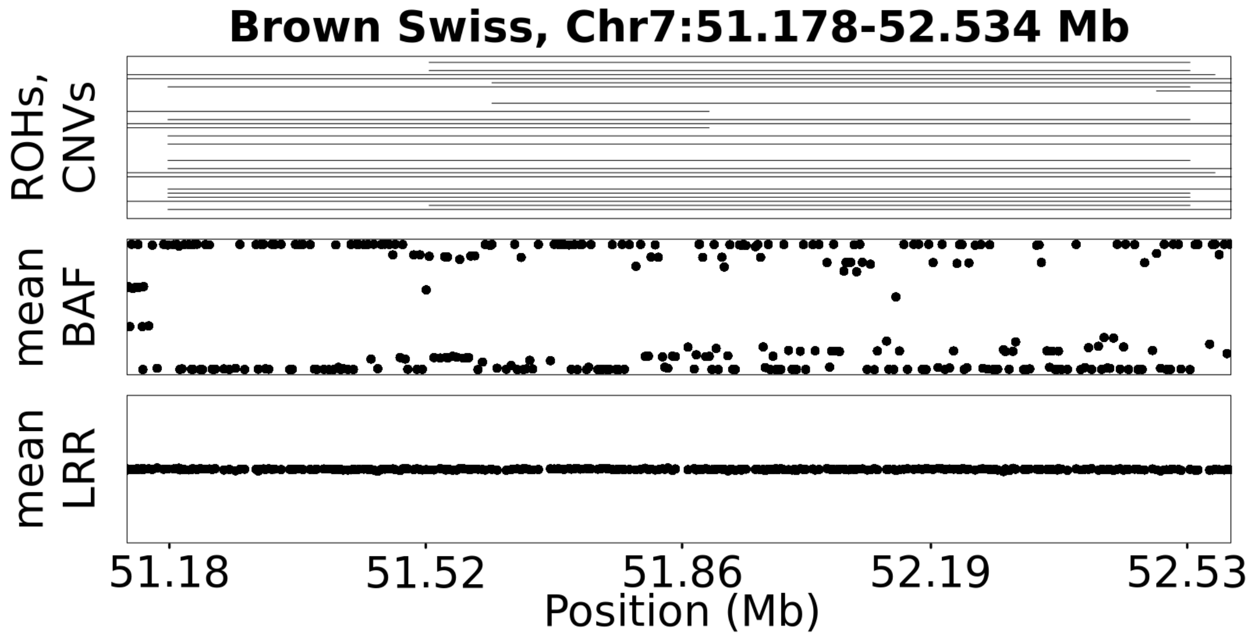
Brown Swiss, Chr7:51.178-52.534 Mb
ROHs, CNVs
mean BAF
mean LRR
51.18
51.52
51.86
52.19
52.53
Position (Mb)

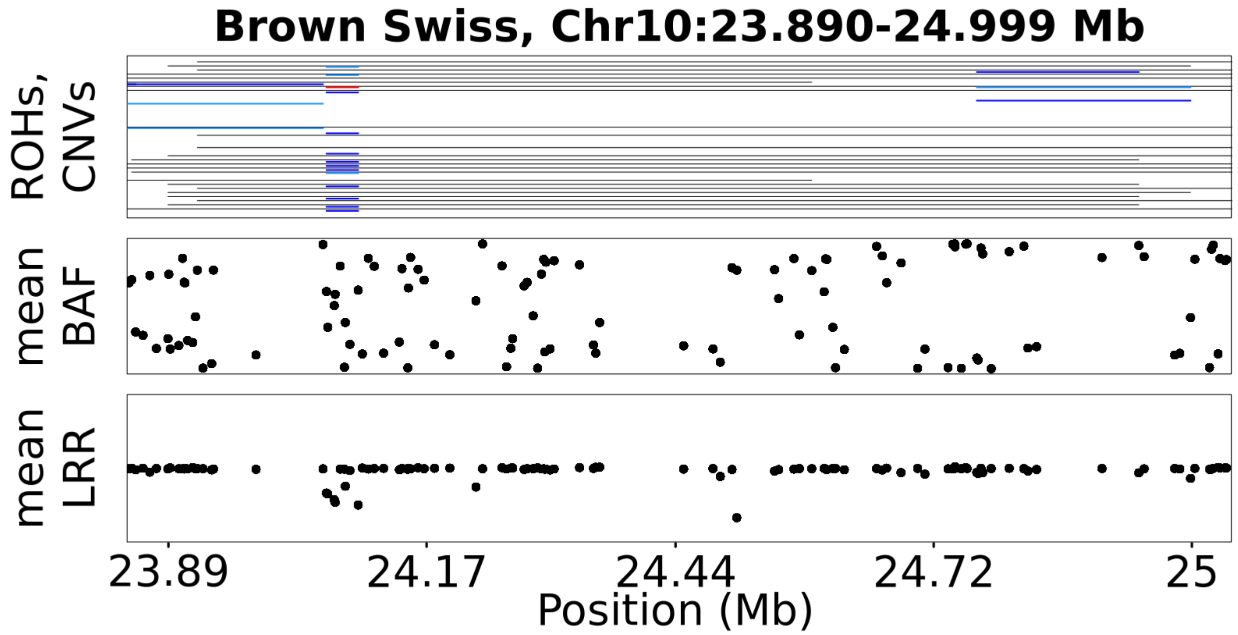
Brown Swiss, Chr10:23.890-24.999 Mb
ROHs, CNVs
mean BAF
mean LRR
23.89
24.17
24.44
24.72
25
Position (Mb)

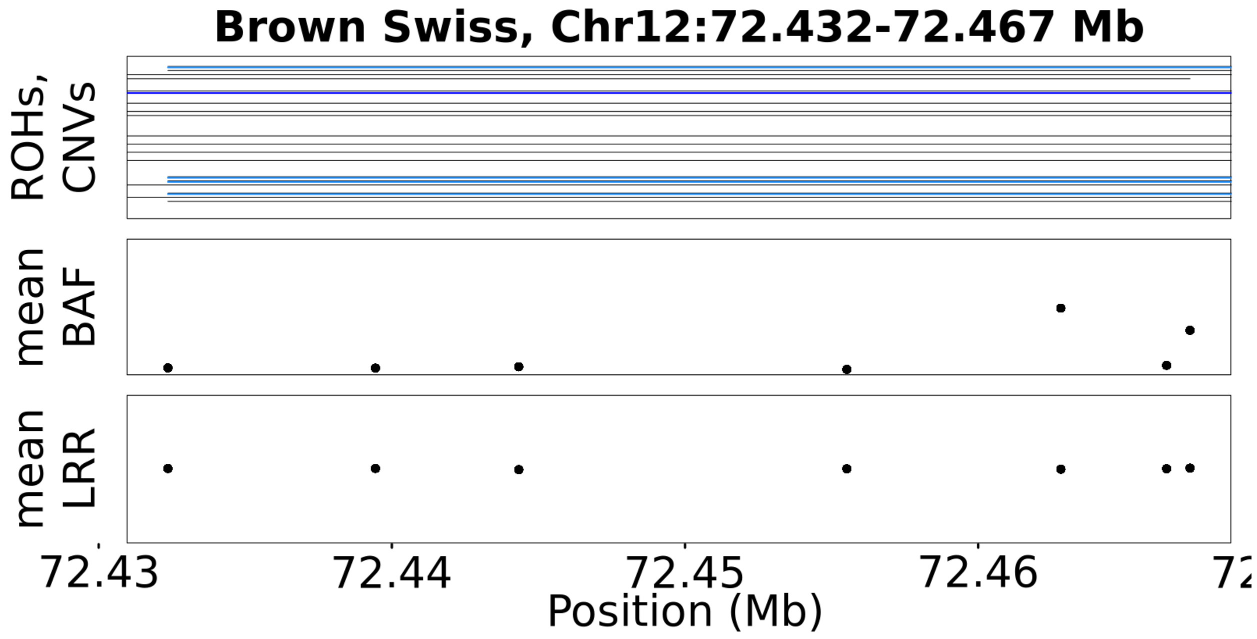
Brown Swiss, Chr12:72.432-72.467 Mb
ROHs, CNVs
mean BAF
mean LRR
72.43
72.44
72.45
72.46
Position (Mb)

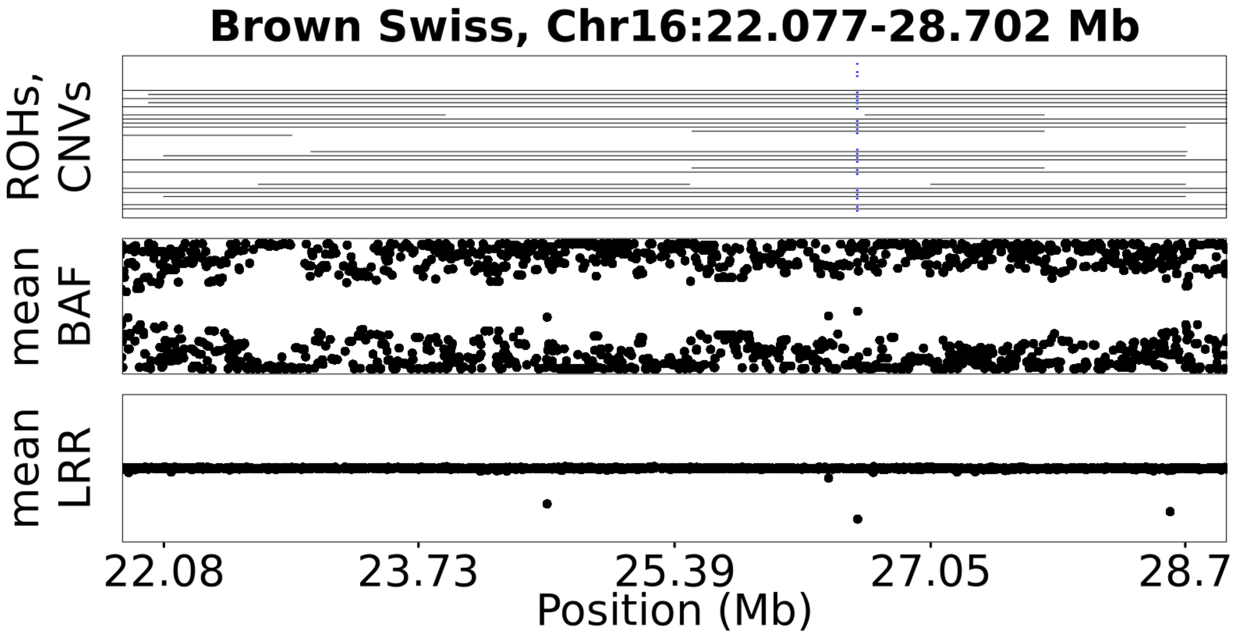
Brown Swiss, Chr16:22.077-28.702 Mb
ROHs, CNVs
mean BAF
mean LRR
22.08
23.73
25.39
27.05
28.7
Position (Mb)

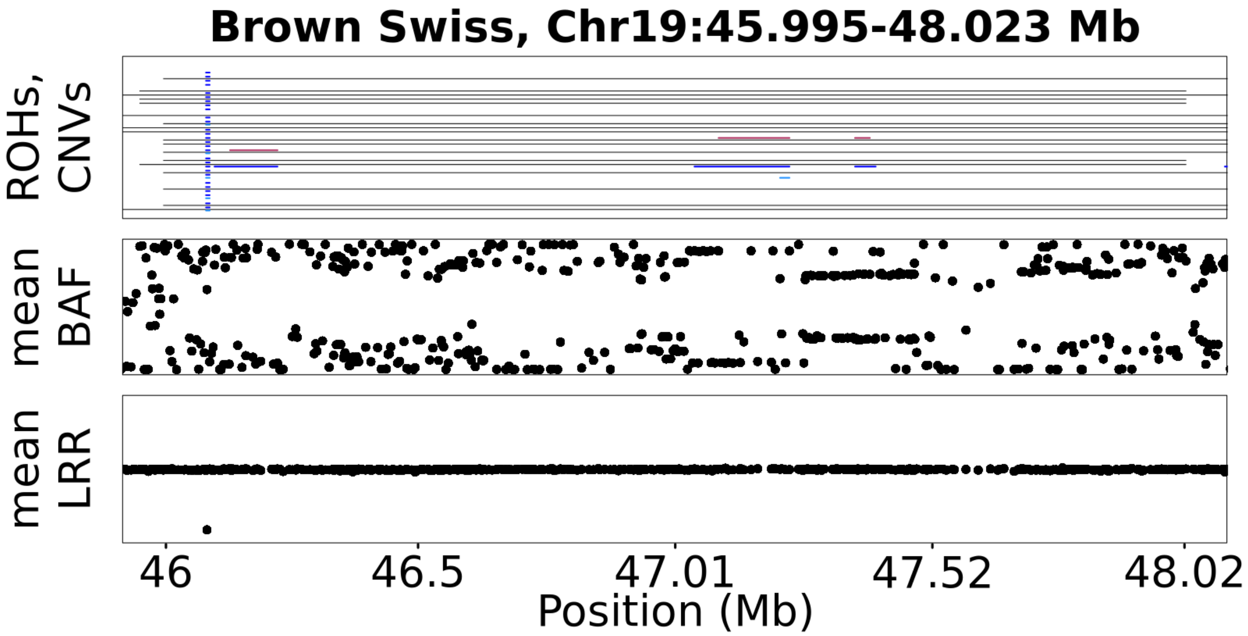
Brown Swiss, Chr19:45.995-48.023 Mb
ROHs, CNVs
mean BAF
mean LRR
46
46.5
47.01
47.52
48.02
Position (Mb)

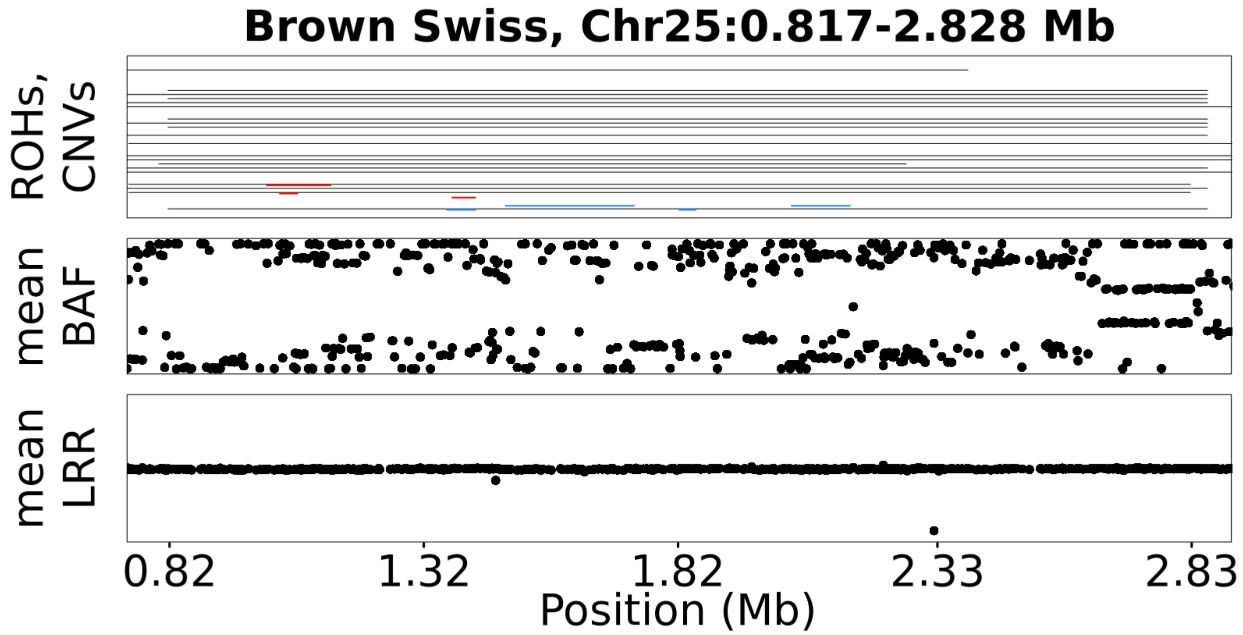
Brown Swiss, Chr25:0.817-2.828 Mb
ROHs, CNVs
mean BAF
mean LRR
0.82
1.32
1.82
2.33
2.83
Position (Mb)

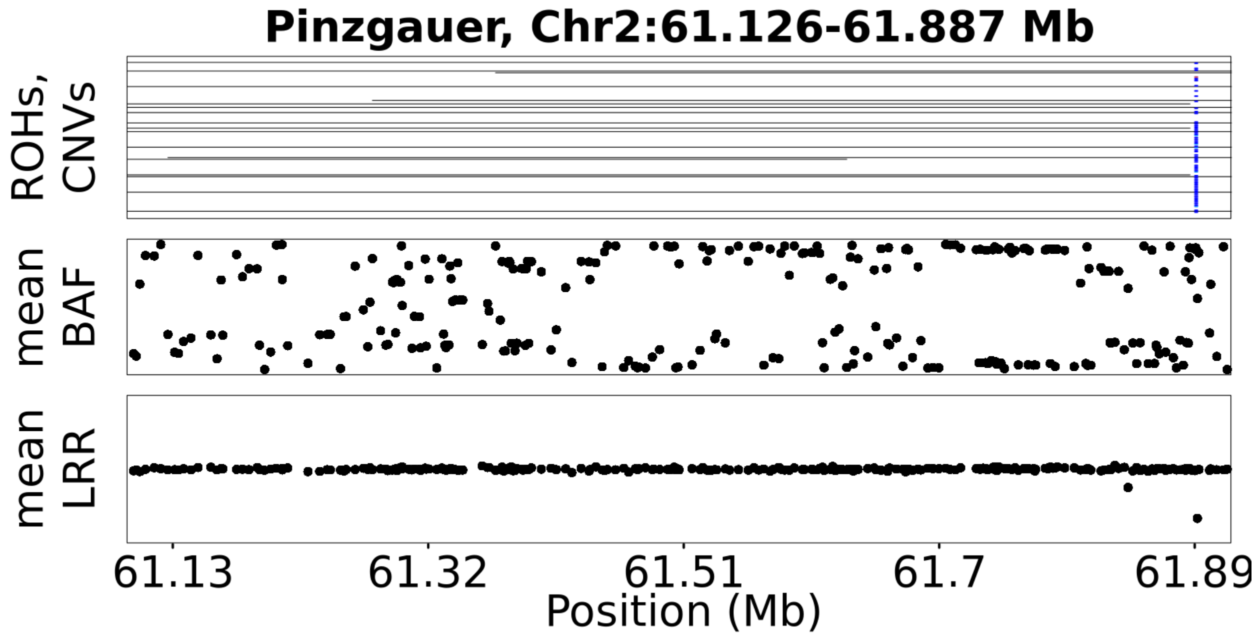
Pinzgauer, Chr2:61.126-61.887 Mb
ROHs, CNVs
mean BAF
mean LRR
61.13
61.32
61.51
61.7
61.89
Position (Mb)

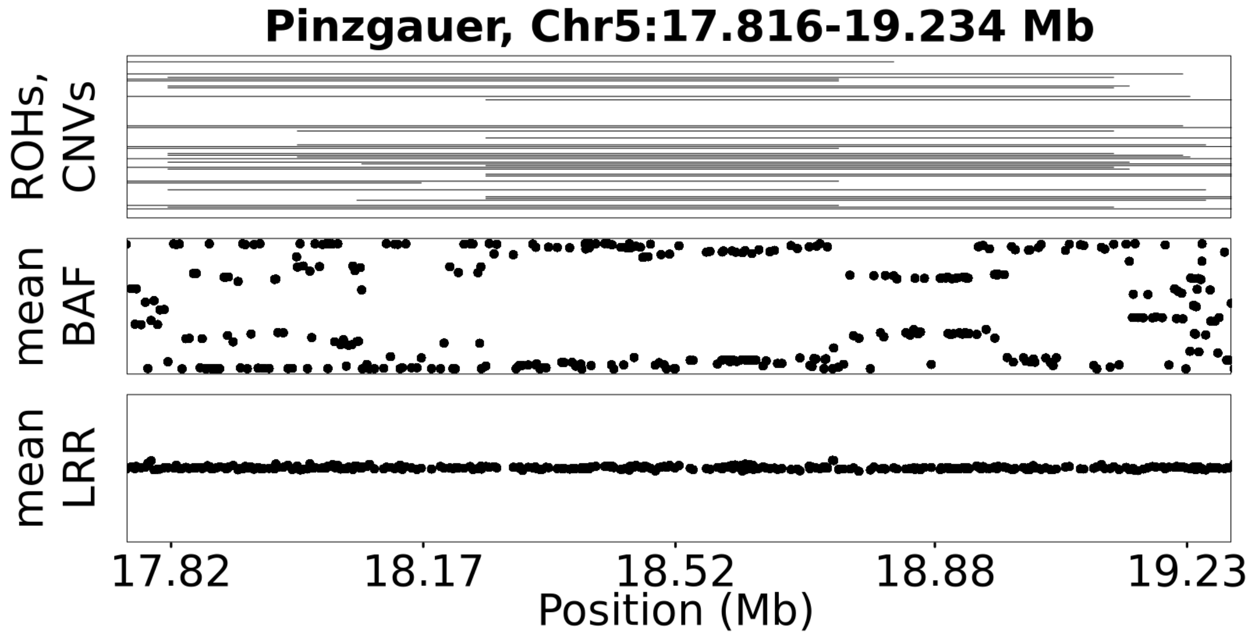
Pinzgauer, Chr5:17.816-19.234 Mb
ROHs, CNVs
mean BAF
mean LRR
17.82
18.17
18.52
18.88
19.23
Position (Mb)

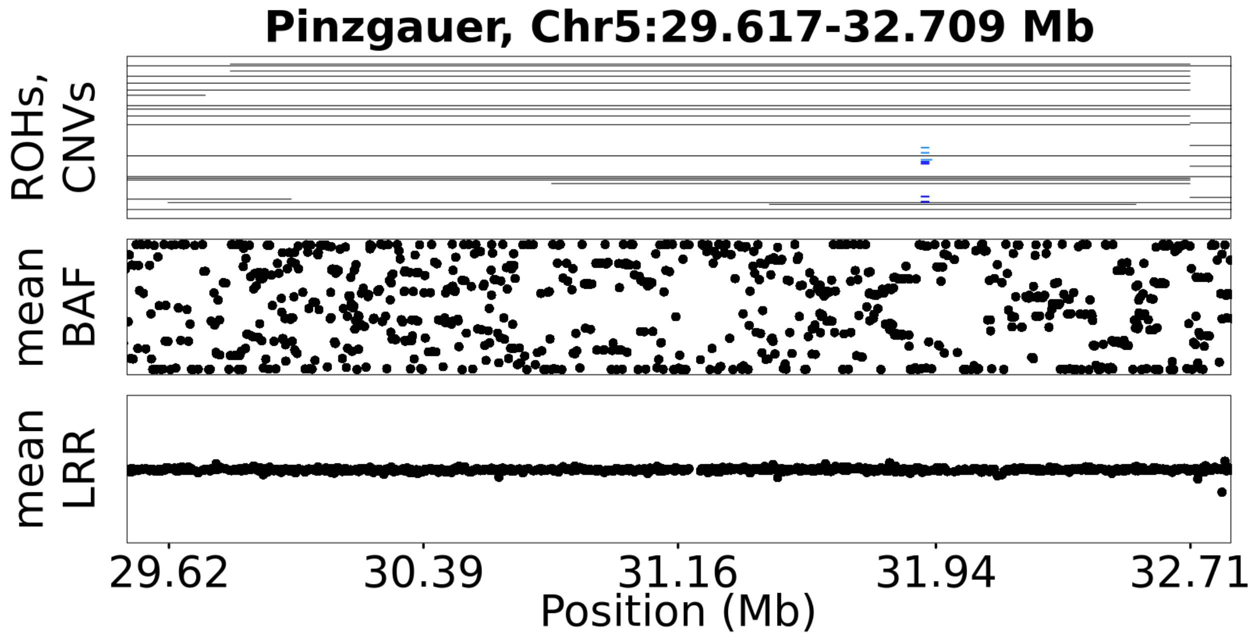
Pinzgauer, Chr5:29.617-32.709 Mb
ROHs, CNVs
mean BAF
mean LRR
29.62
30.39
31.16
31.94
32.71
Position (Mb)

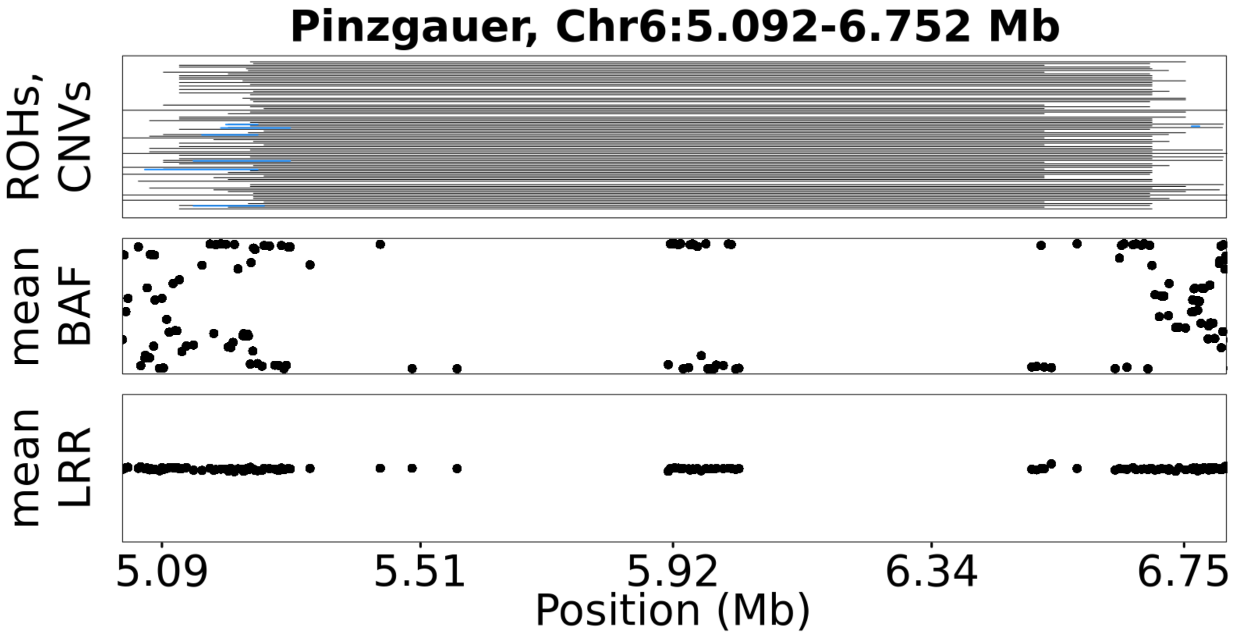
Pinzgauer, Chr6:5.092-6.752 Mb
ROHs, CNVs
mean BAF
mean LRR
5.09
5.51
5.92
6.34
6.75
Position (Mb)

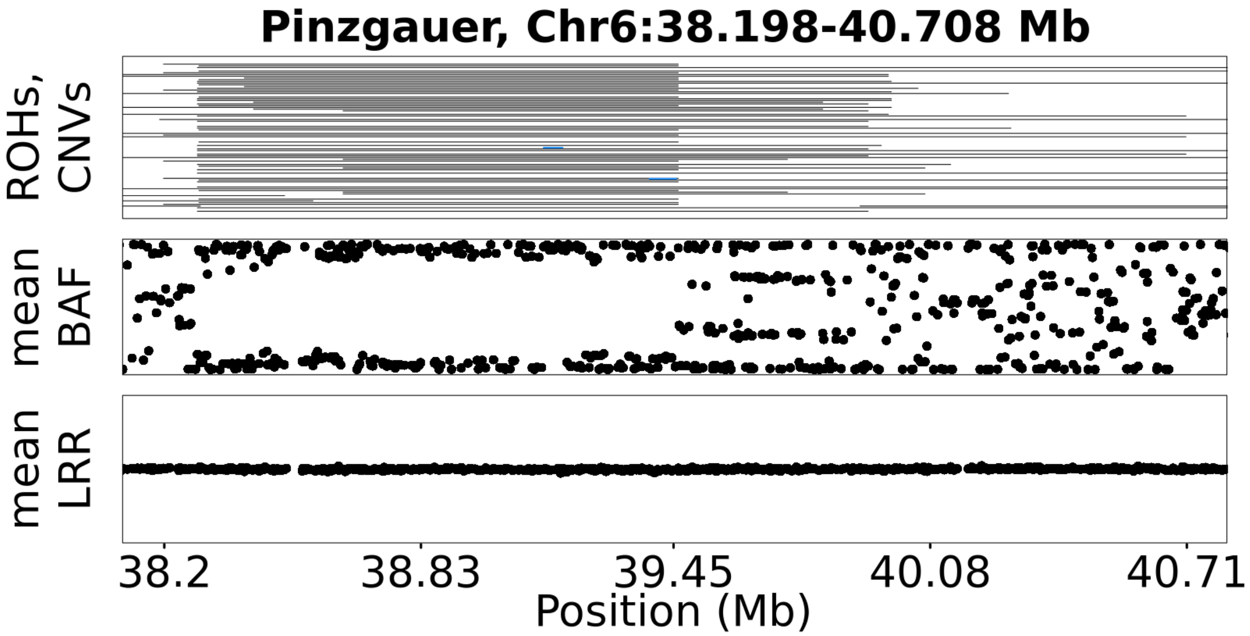
Pinzgauer, Chr6:38.198-40.708 Mb
ROHs, CNVs
mean BAF
mean LRR
38.2
38.83
39.45
40.08
40.71
Position (Mb)

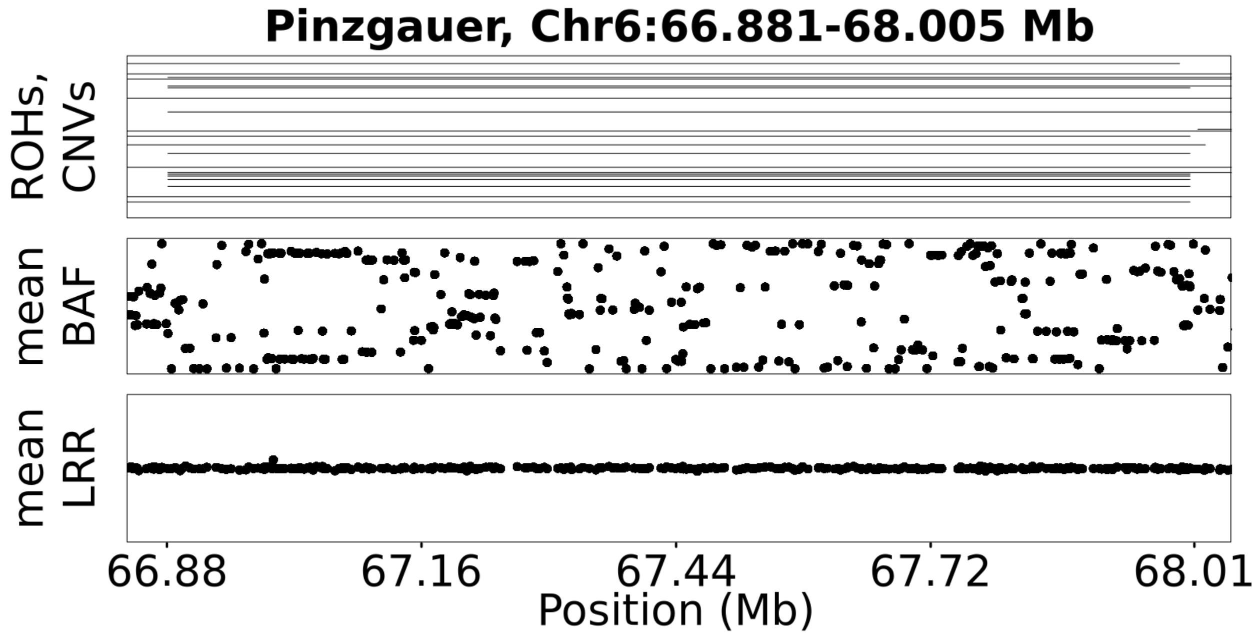
Pinzgauer, Chr6:66.881-68.005 Mb
ROHs, CNVs
mean BAF
mean LRR
66.88
67.16
67.44
67.72
68.01
Position (Mb)

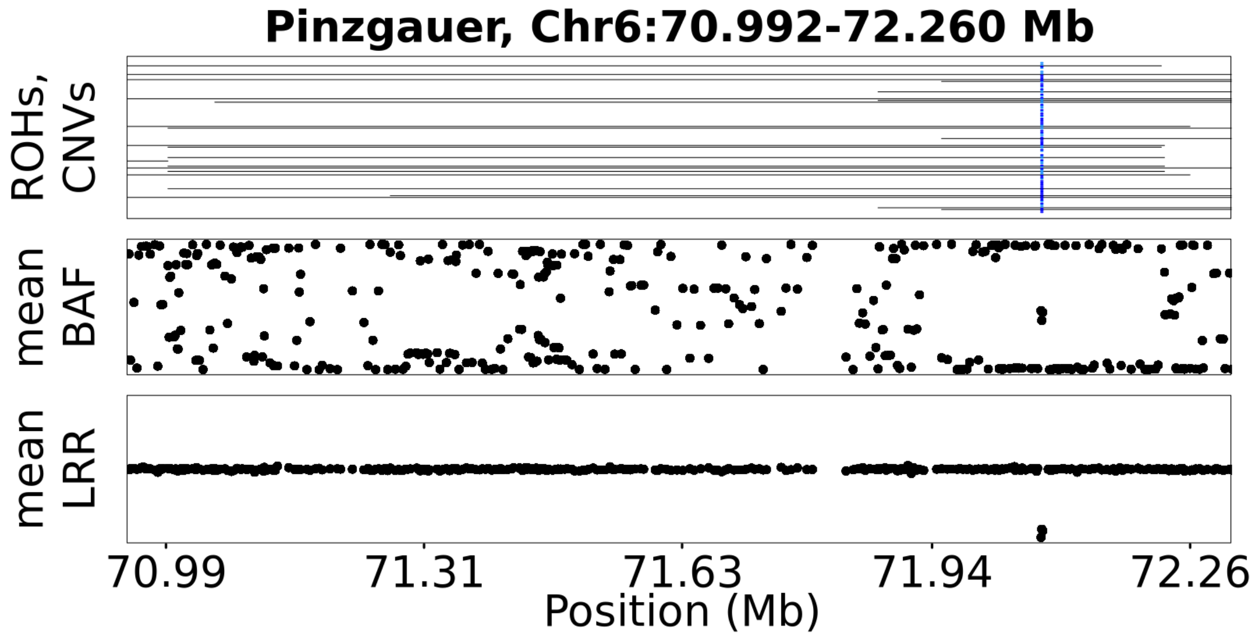
Pinzgauer, Chr6:70.992-72.260 Mb
ROHs, CNVs
mean BAF
mean LRR
70.99
71.31
71.63
71.94
72.26
Position (Mb)

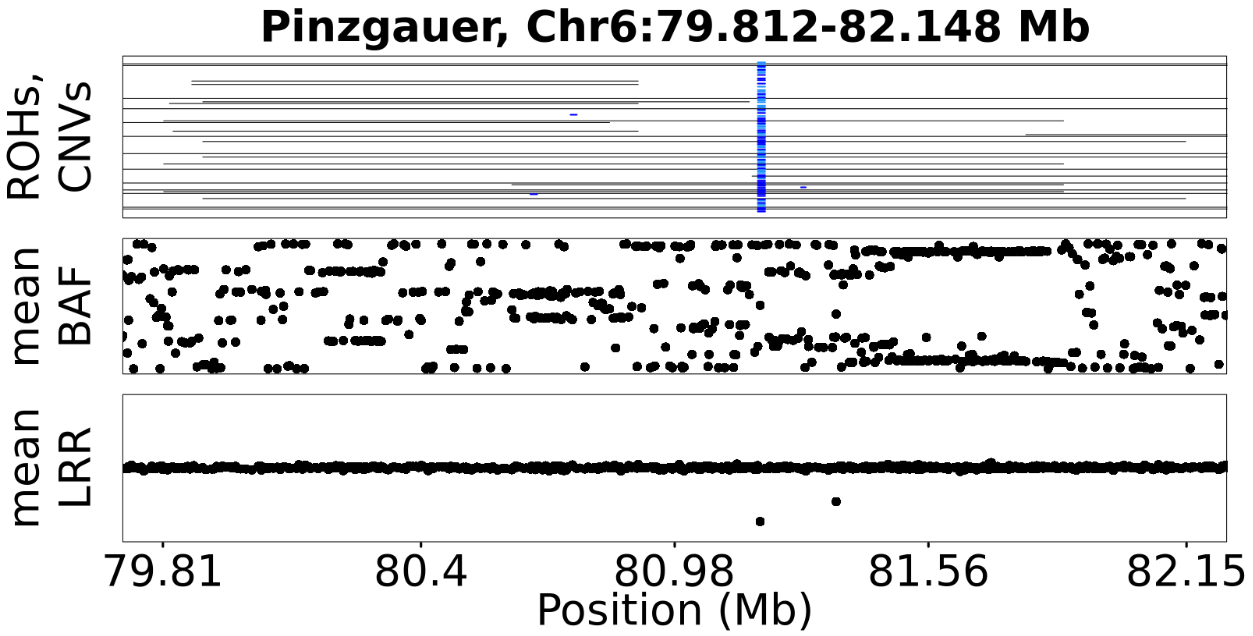

Pinzgauer, Chr6:79.812-82.148 Mb
ROHs, CNVs
mean BAF
mean LRR
79.81
80.4
80.98
81.56
82.15
Position (Mb)

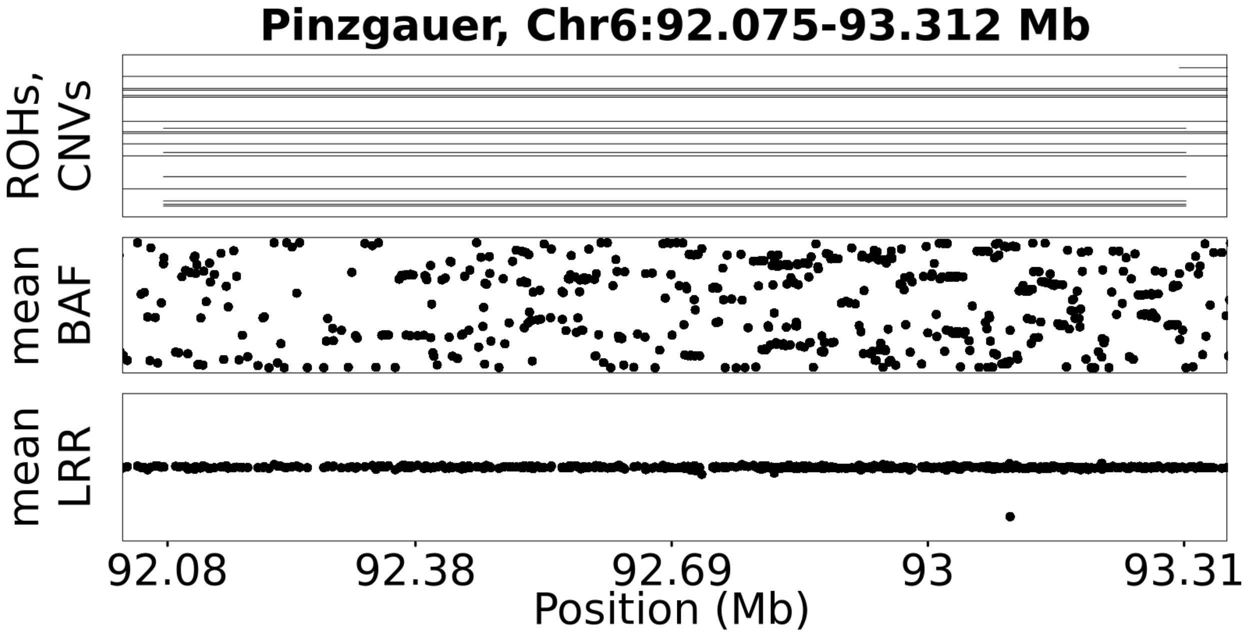

Pinzgauer, Chr6:92.075-93.312 Mb
ROHs, CNVs
mean BAF
mean LRR
92.08
92.38
92.69
93
93.31
Position (Mb)

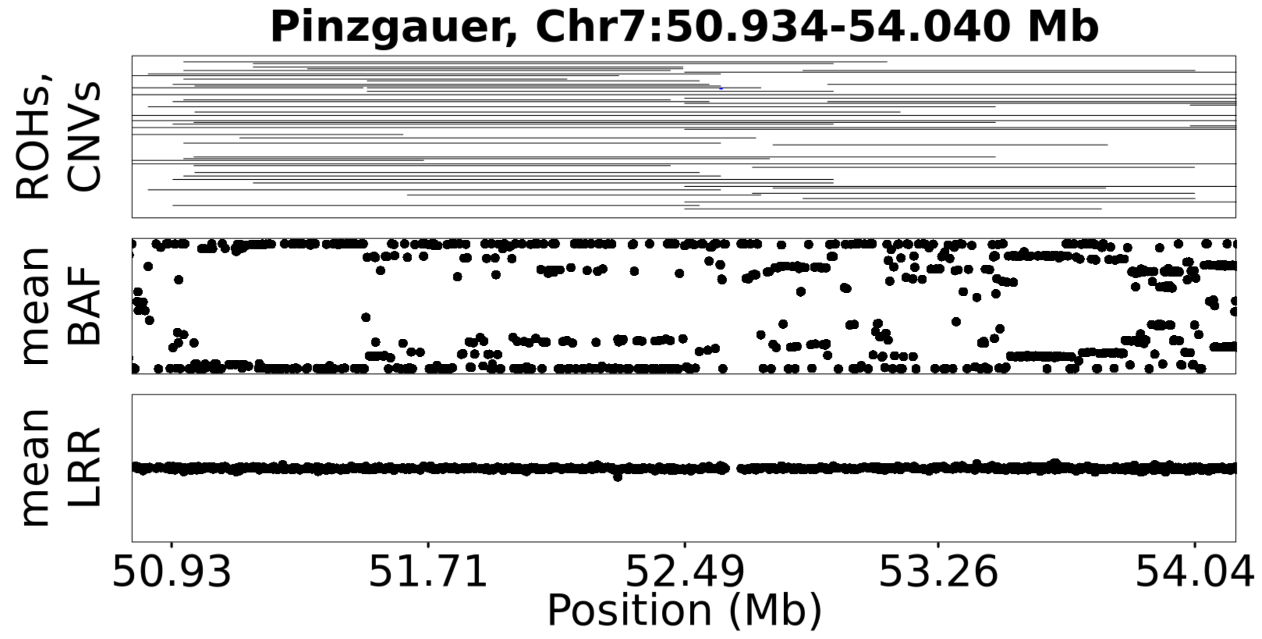
Pinzgauer, Chr7:50.934-54.040 Mb
ROHs, CNVs
mean BAF
mean LRR
50.93
51.71
52.49
53.26
54.04
Position (Mb)

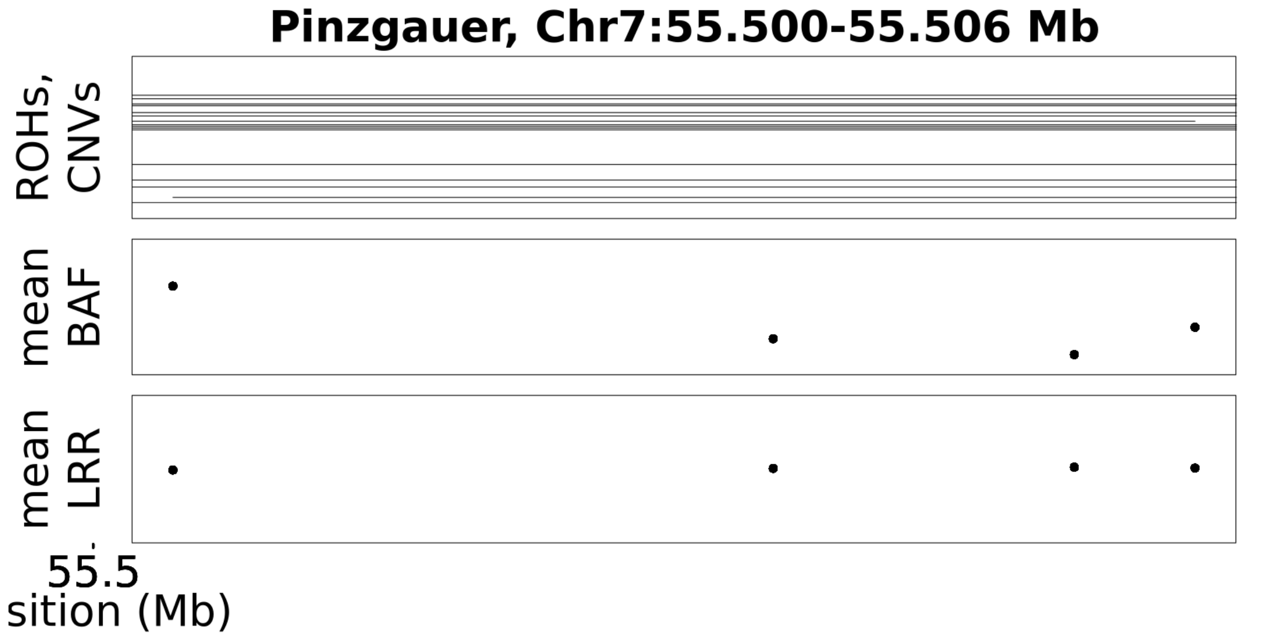
Pinzgauer, Chr7:55.500-55.506 Mb
ROHs, CNVs
mean BAF
mean LRR
55.5
sition (Mb)

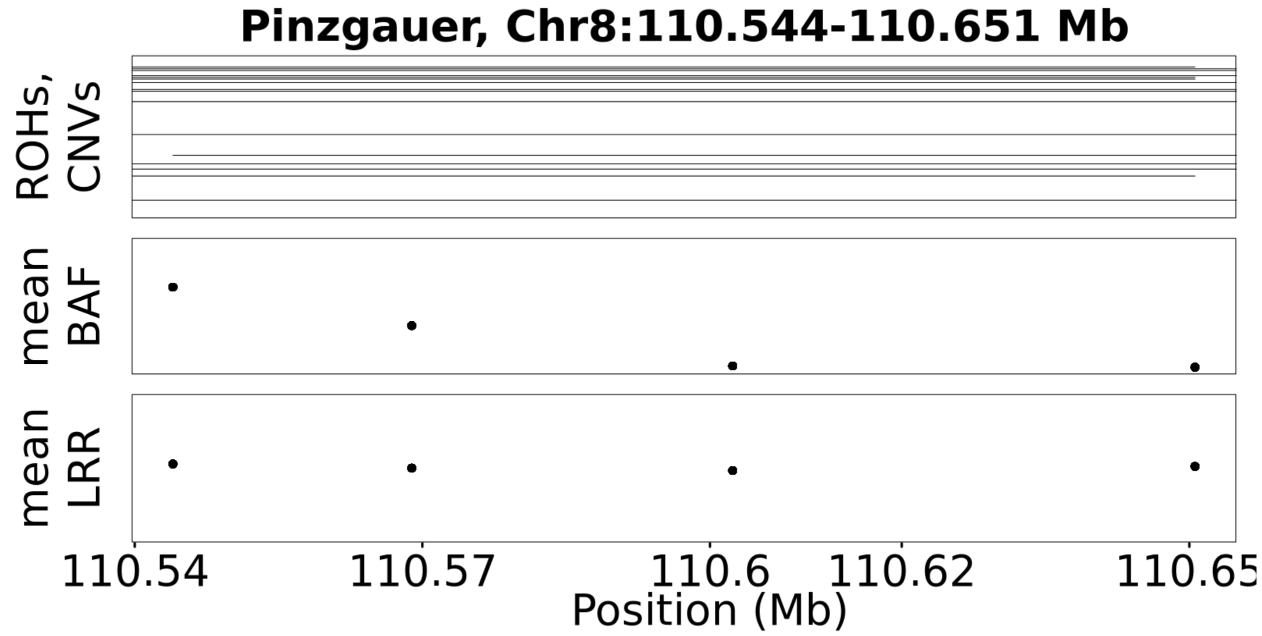
Pinzgauer, Chr8:110.544-110.651 Mb
ROHs, CNVs
mean BAF
mean LRR
110.54
110.57
110.6
110.62
110.65
Position (Mb)

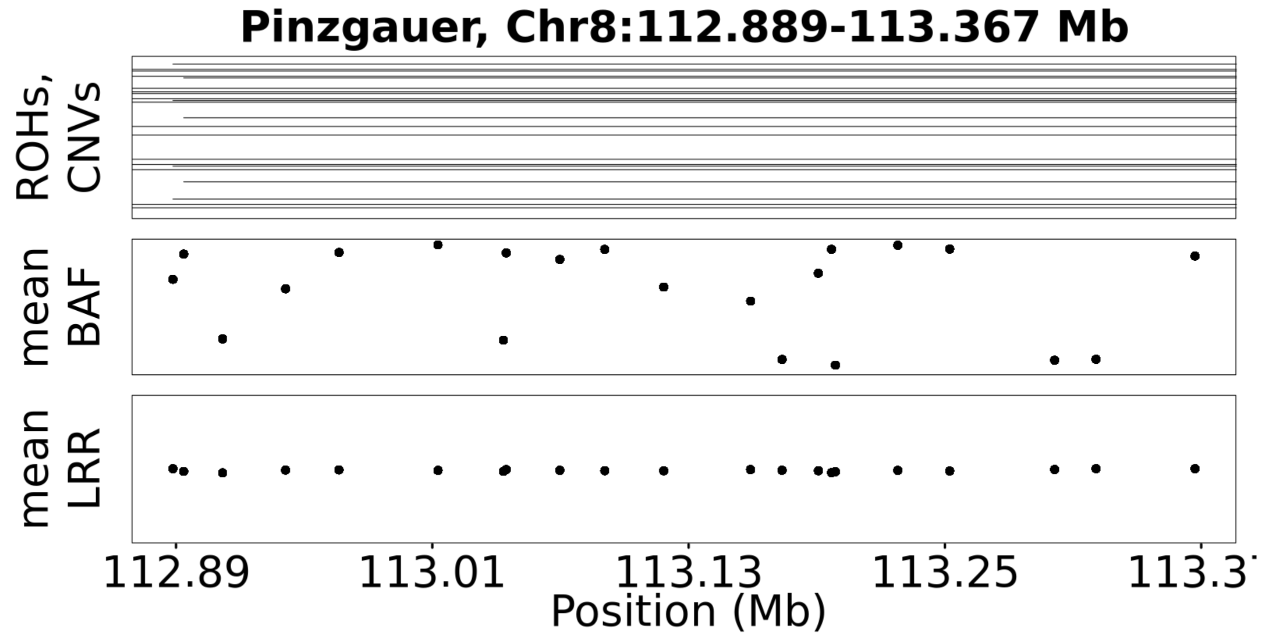
Pinzgauer, Chr8:112.889-113.367 Mb
ROHs, CNVs
mean BAF
mean LRR
112.89
113.01
113.13
113.25
Position (Mb)

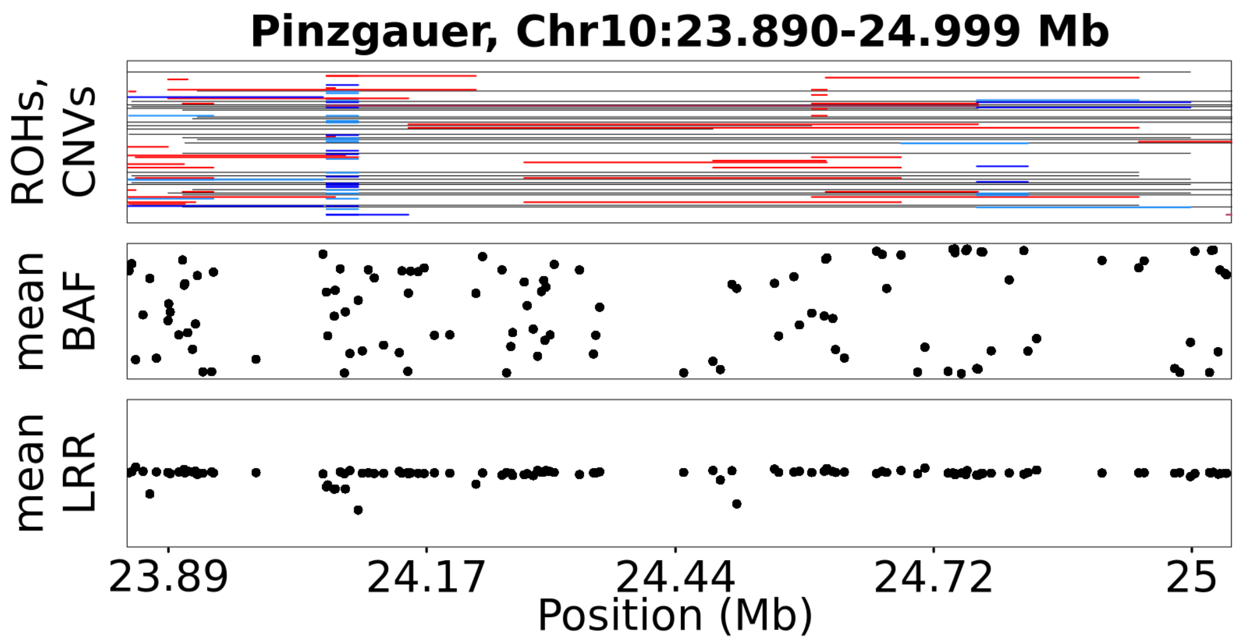
Pinzgauer, Chr10:23.890-24.999 Mb
ROHs, CNVs
mean BAF
mean LRR
23.89
24.17
24.44
24.72
25
Position (Mb)

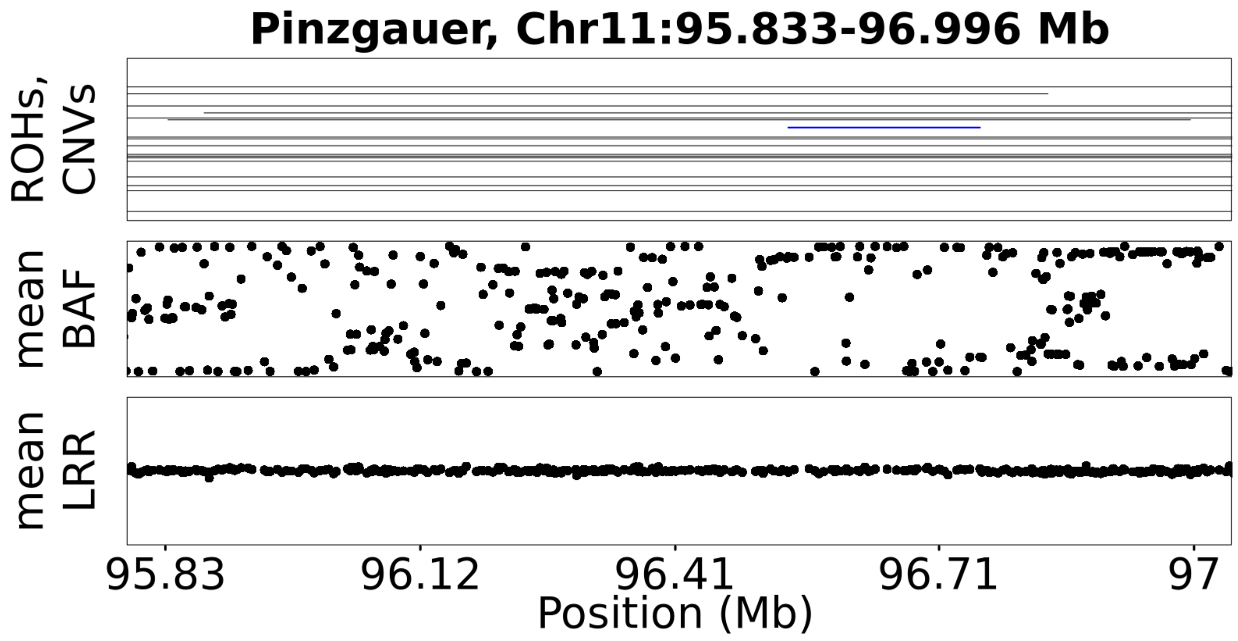
Pinzgauer, Chr11:95.833-96.996 Mb
ROHs, CNVs
mean BAF
mean LRR
95.83
96.12
96.41
96.71
97
Position (Mb)

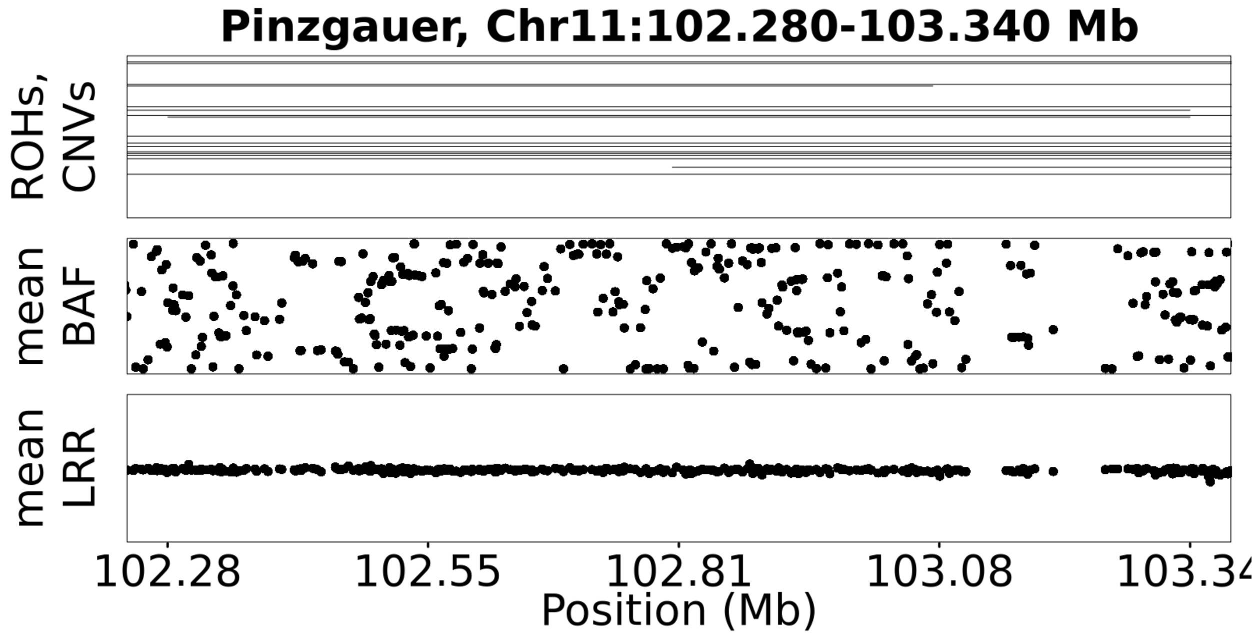
Pinzgauer, Chr11:102.280-103.340 Mb
ROHs, CNVs
mean BAF
mean LRR
102.28
102.55
102.81
103.08
Position (Mb)

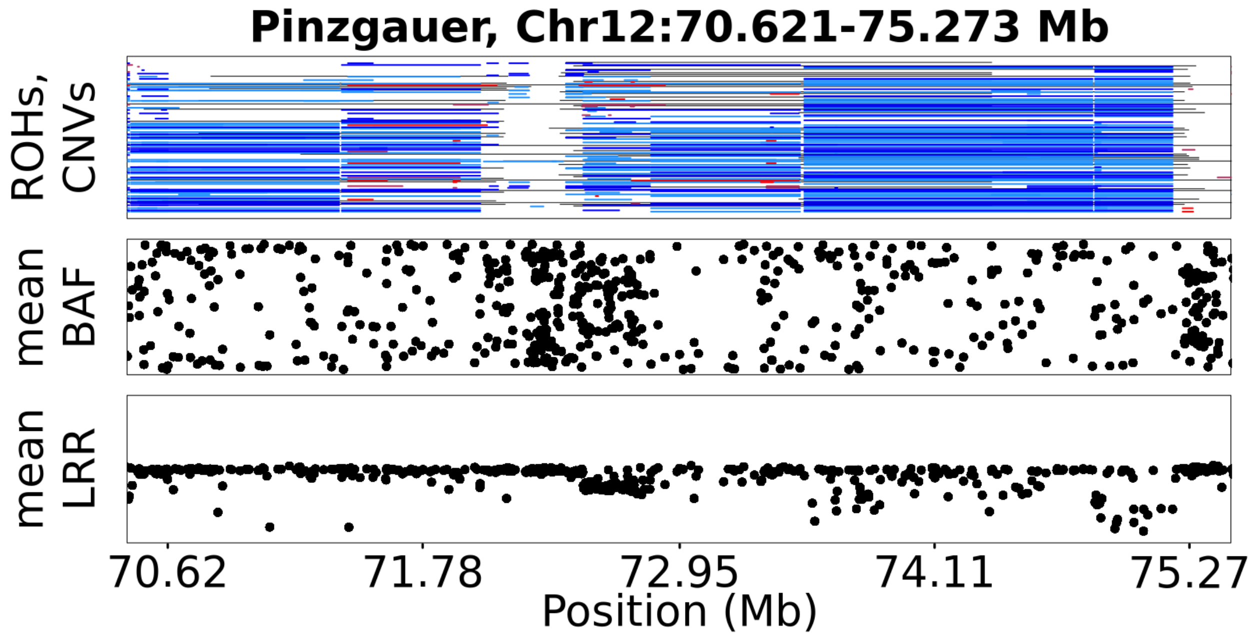
Pinzgauer, Chr12:70.621-75.273 Mb
ROHs, CNVs
mean BAF
mean LRR
70.62
71.78
72.95
74.11
75.27
Position (Mb)

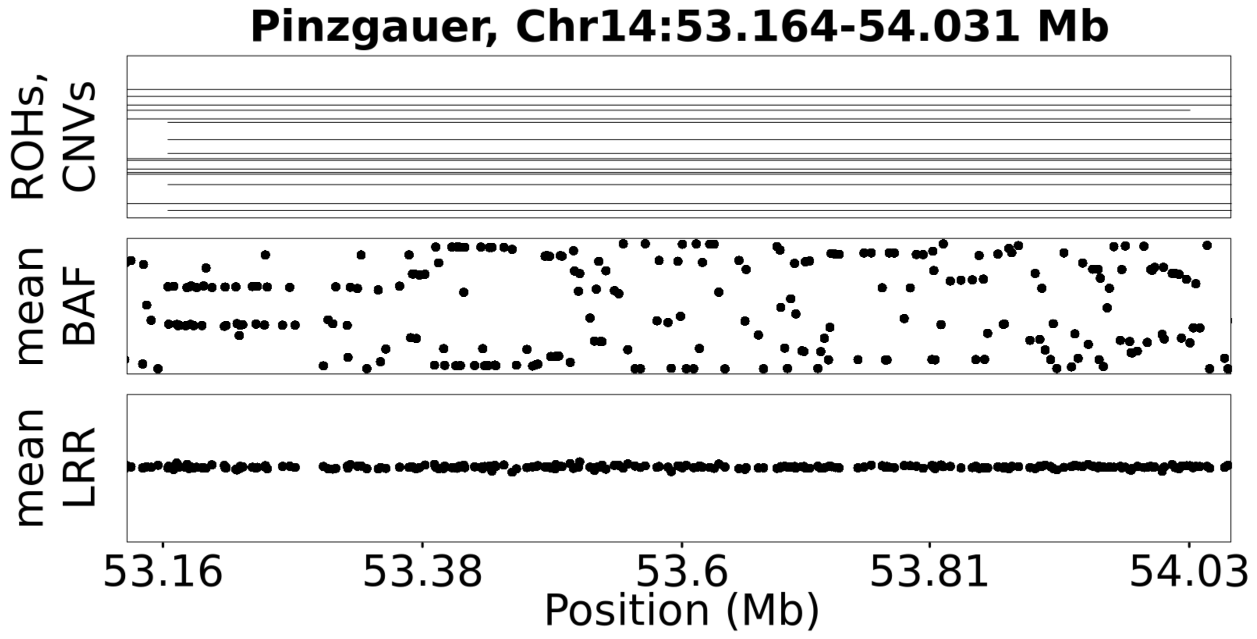
Pinzgauer, Chr14:53.164-54.031 Mb
ROHs, CNVs
mean BAF
mean LRR
53.16
53.38
53.6
53.81
54.03
Position (Mb)

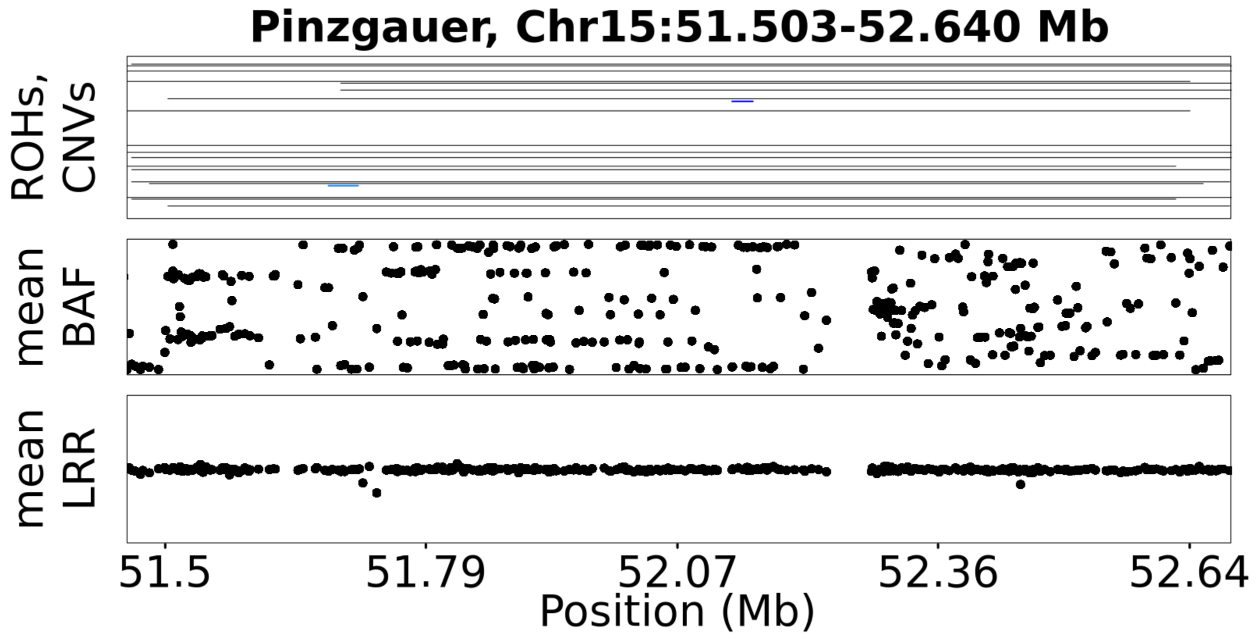
Pinzgauer, Chr15:51.503-52.640 Mb
ROHs, CNVs
mean BAF
mean LRR
51.5
51.79
52.07
52.36
52.64
Position (Mb)

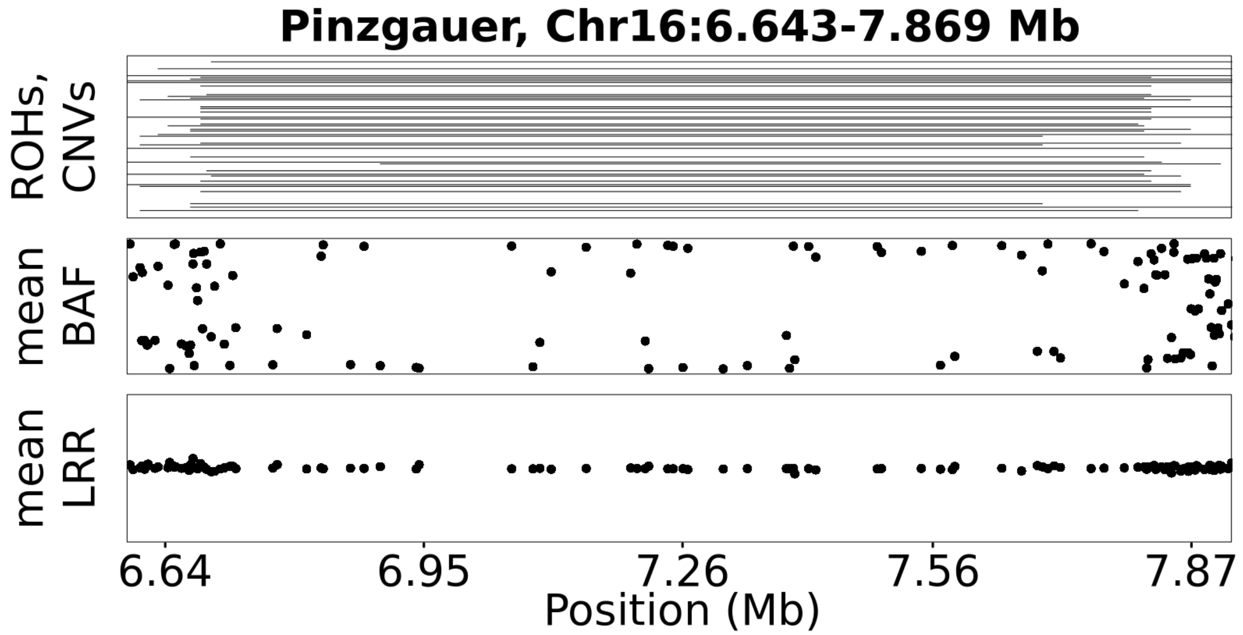
Pinzgauer, Chr16:6.643-7.869 Mb
ROHs, CNVs
mean BAF
mean LRR
6.64
6.95
7.26
7.56
7.87
Position (Mb)

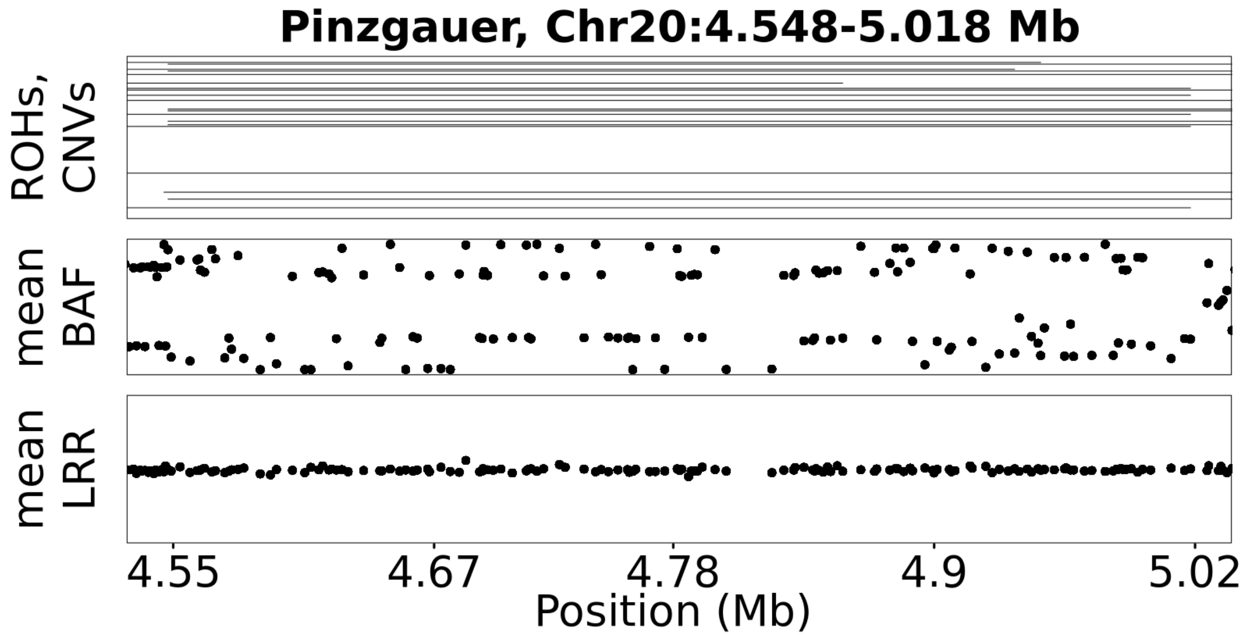
Pinzgauer, Chr20:4.548-5.018 Mb
ROHs, CNVs
mean BAF
mean LRR
4.55
4.67
4.78
4.9
5.02
Position (Mb)

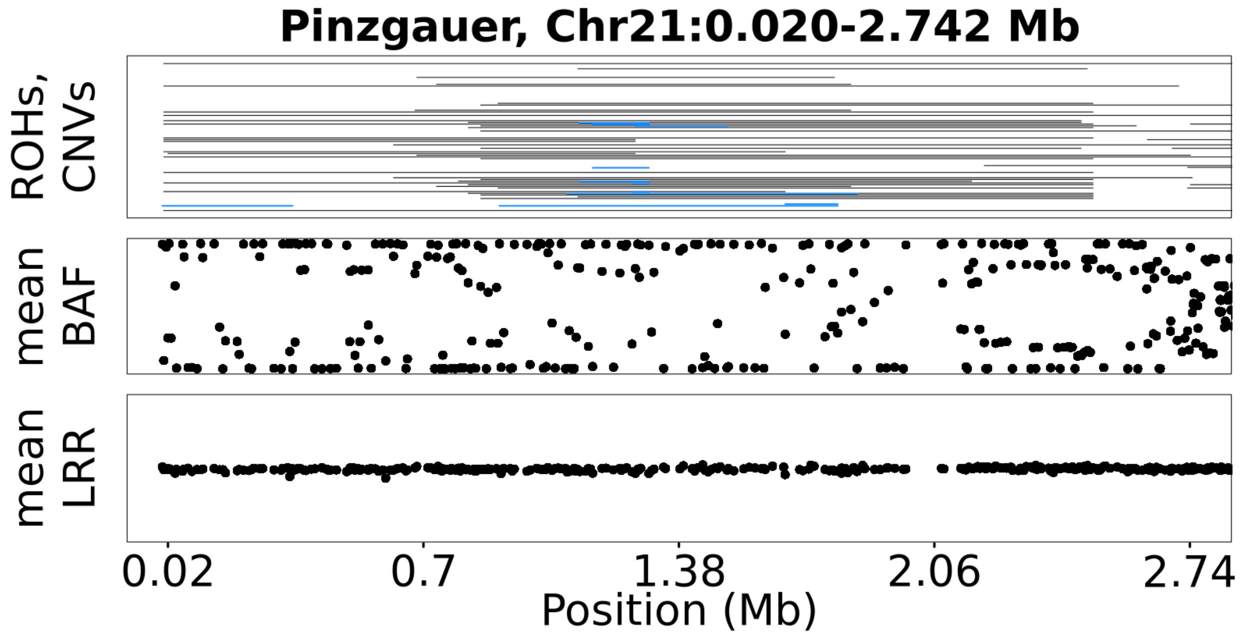
Pinzgauer, Chr21:0.020-2.742 Mb
ROHs, CNVs
mean BAF
mean LRR
0.02
0.7
1.38
2.06
2.74
Position (Mb)

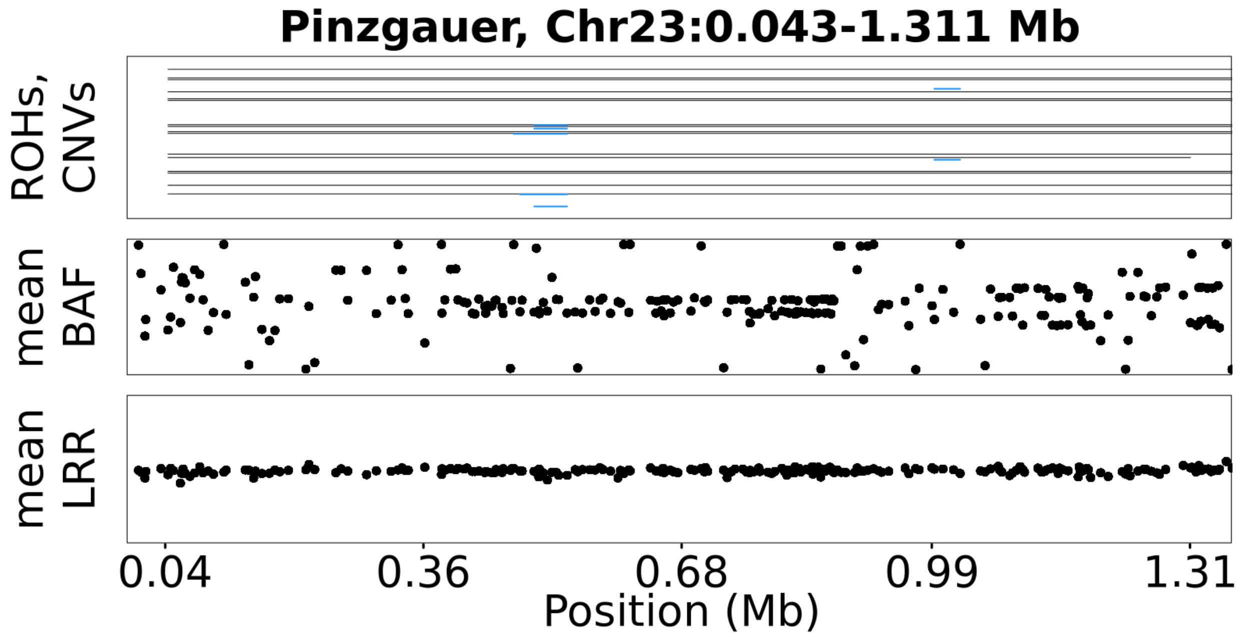
Pinzgauer, Chr23:0.043-1.311 Mb
ROHs, CNVs
mean BAF
mean LRR
0.04
0.36
0.68
0.99
1.31
Position (Mb)

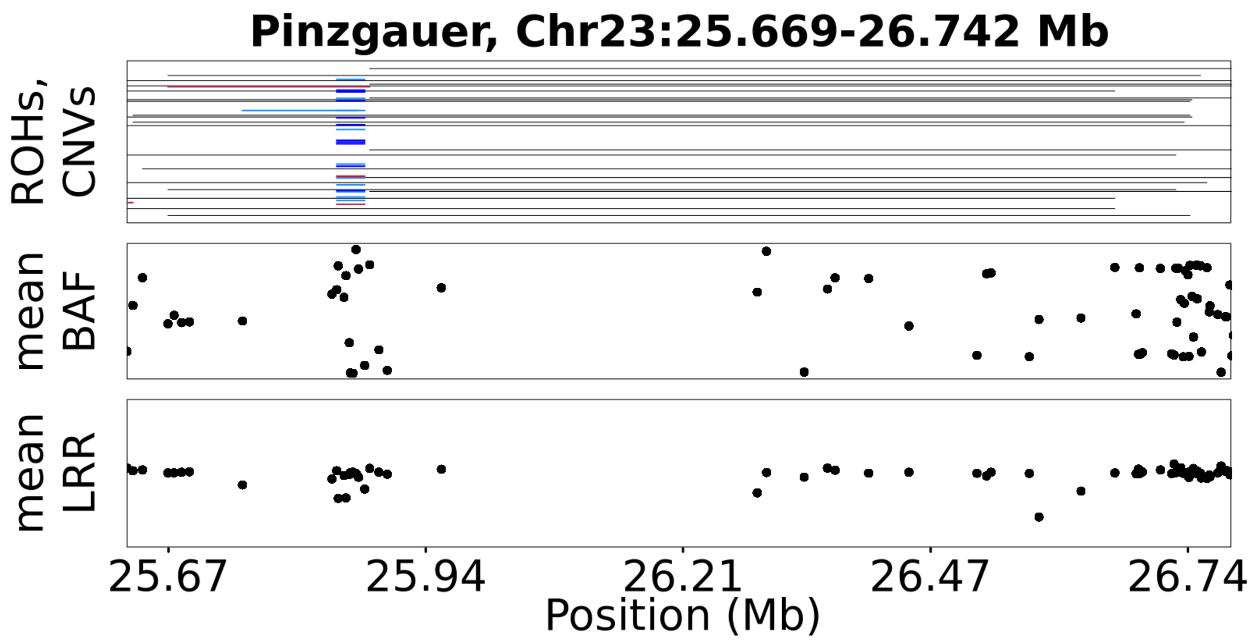
Pinzgauer, Chr23:25.669-26.742 Mb
ROHs, CNVs
mean BAF
mean LRR
25.67
25.94
26.21
26.47
26.74
Position (Mb)

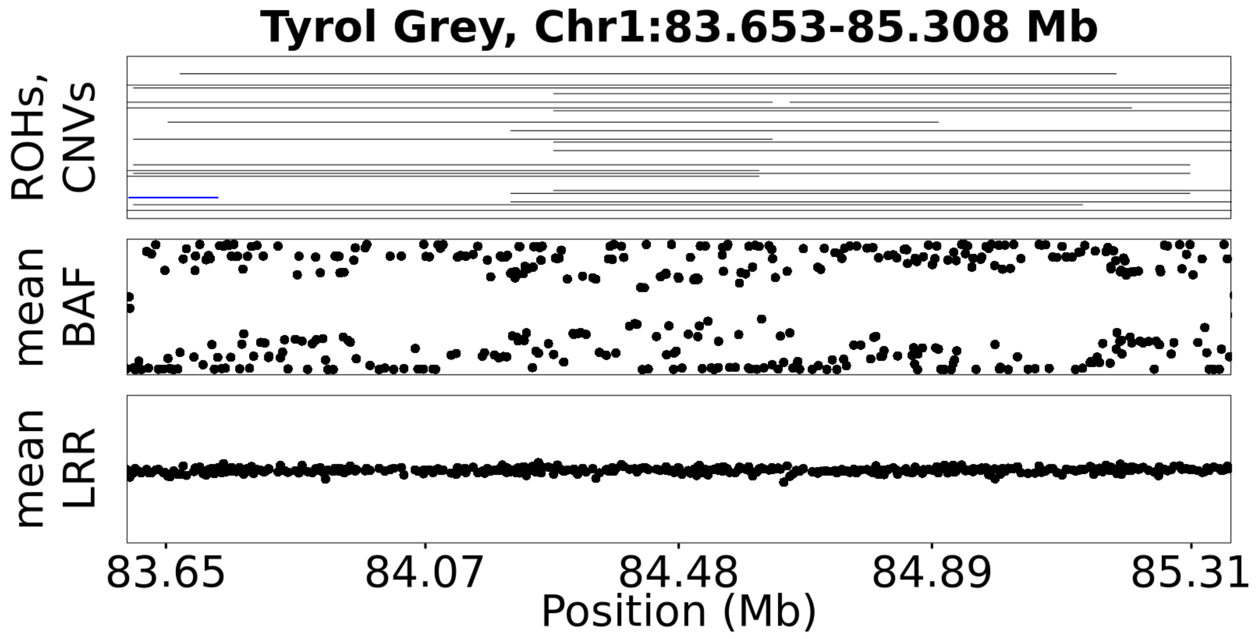
Tyrol Grey, Chr1:83.653-85.308 Mb
ROHs, CNVs
mean BAF
mean LRR
83.65
84.07
84.48
84.89
85.31
Position (Mb)

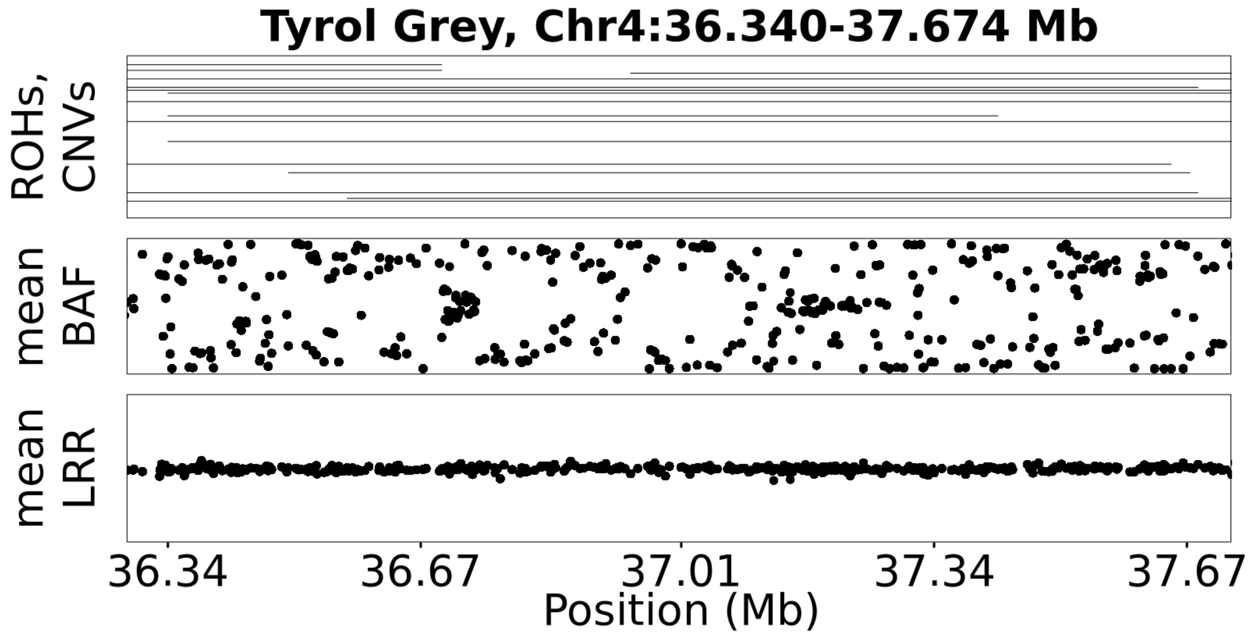
Tyrol Grey, Chr4:36.340-37.674 Mb
ROHs, CNVs
mean BAF
mean LRR
36.34
36.67
37.01
37.34
37.67
Position (Mb)

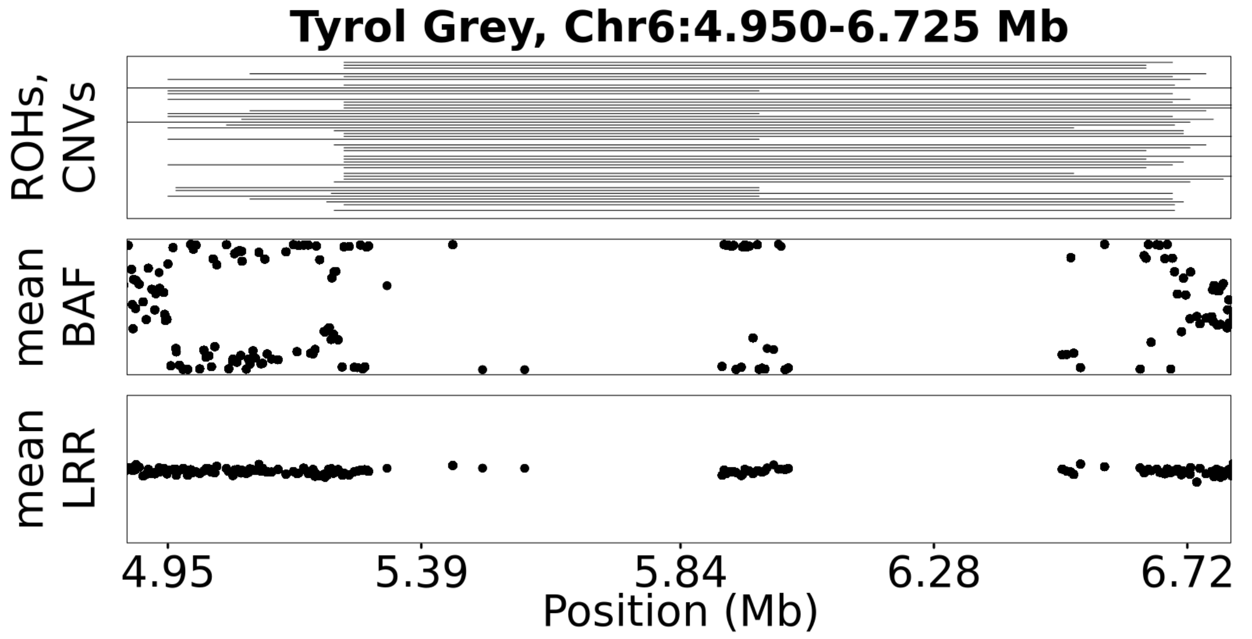
Tyrol Grey, Chr6:4.950-6.725 Mb
ROHs, CNVs
mean BAF
mean LRR
4.95
5.39
5.84
6.28
6.72
Position (Mb)

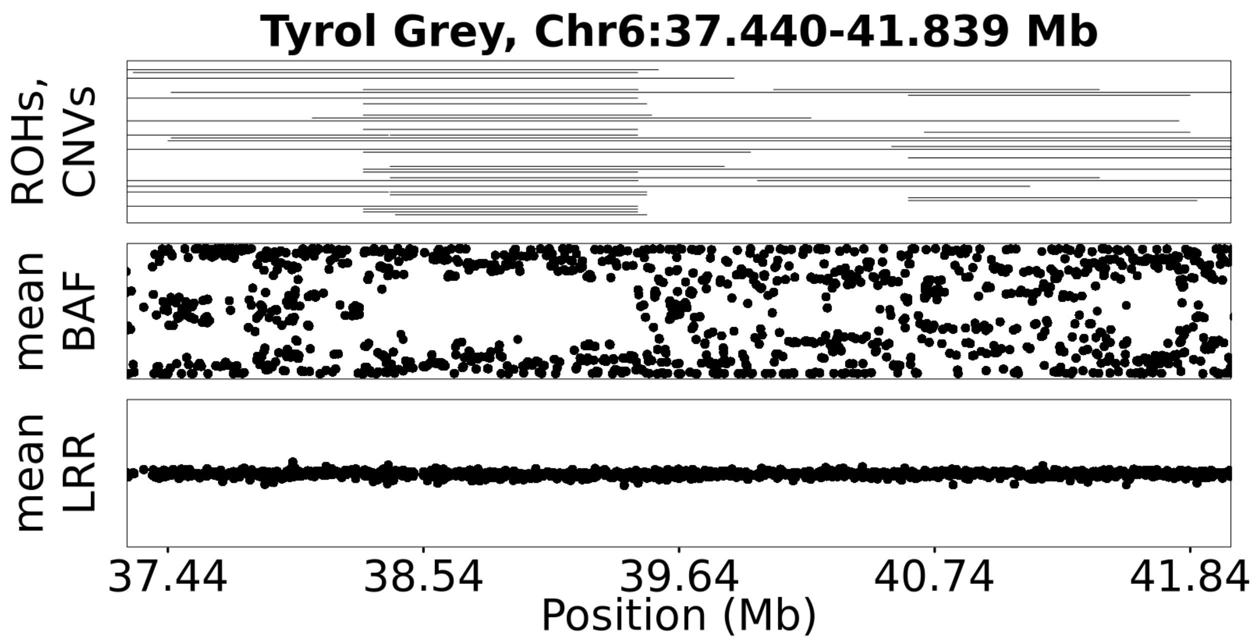
Tyrol Grey, Chr6:37.440-41.839 Mb
ROHs, CNVs
mean BAF
mean LRR
37.44
38.54
39.64
40.74
41.84
Position (Mb)

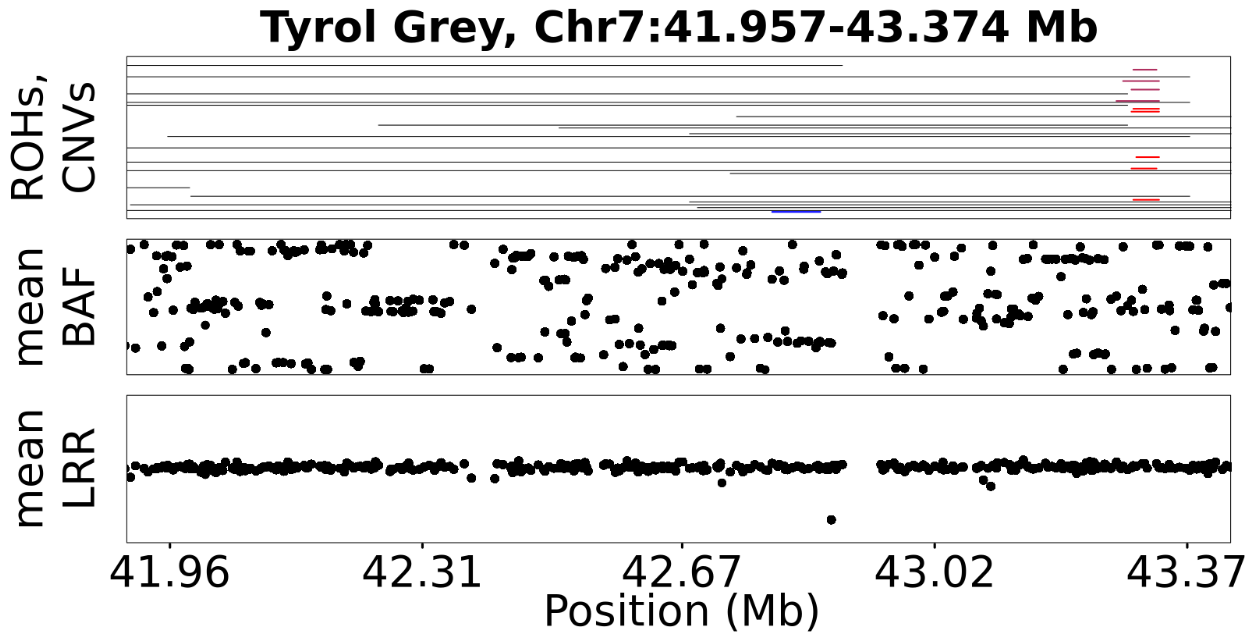
Tyrol Grey, Chr7:41.957-43.374 Mb
ROHs, CNVs
mean BAF
mean LRR
41.96
42.31
42.67
43.02
43.37
Position (Mb)

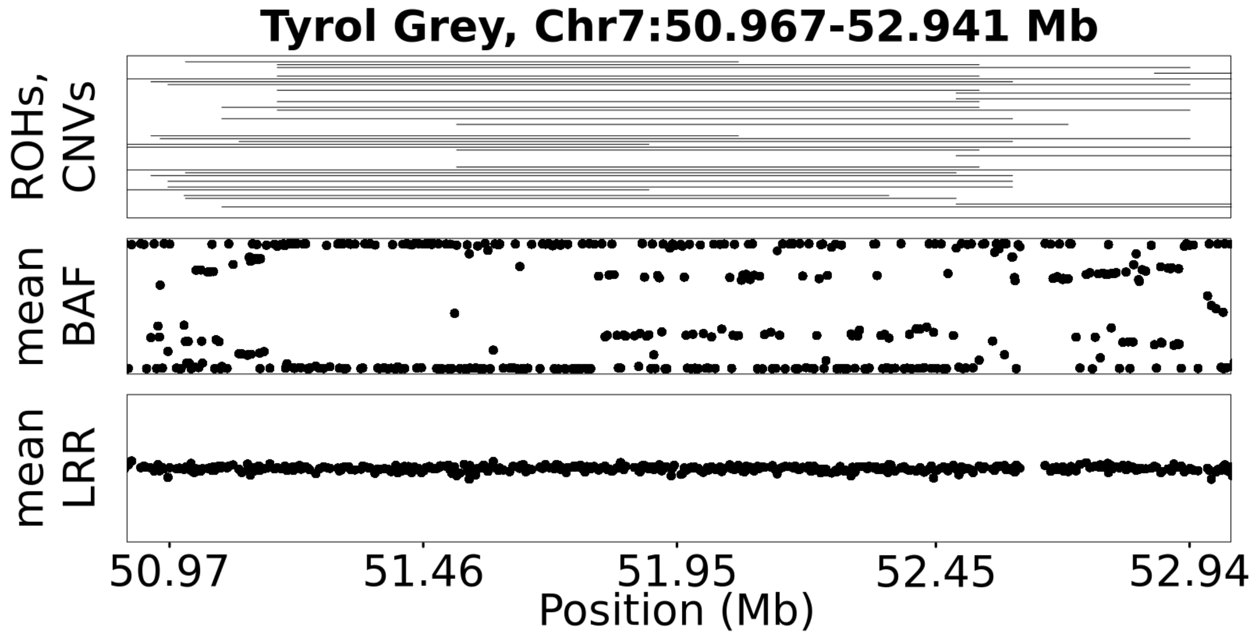
Tyrol Grey, Chr7:50.967-52.941 Mb
ROHs, CNVs
mean BAF
mean LRR
50.97
51.46
51.95
52.45
52.94
Position (Mb)

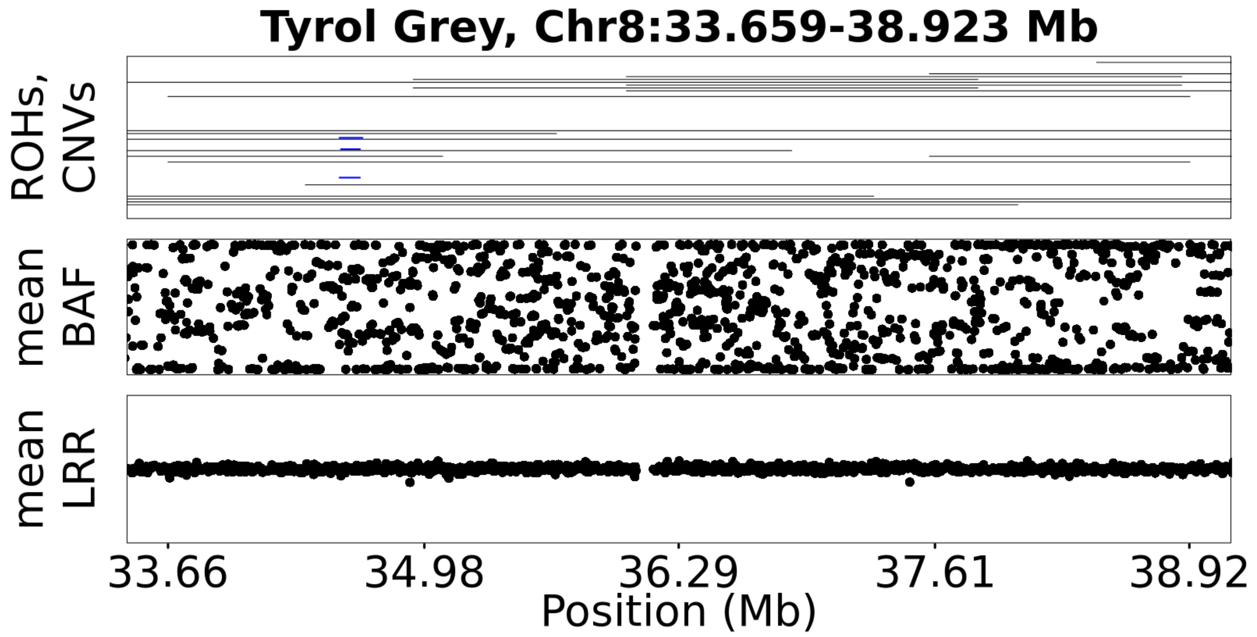
Tyrol Grey, Chr8:33.659-38.923 Mb
ROHs, CNVs
mean BAF
mean LRR
33.66
34.98
36.29
37.61
38.92
Position (Mb)

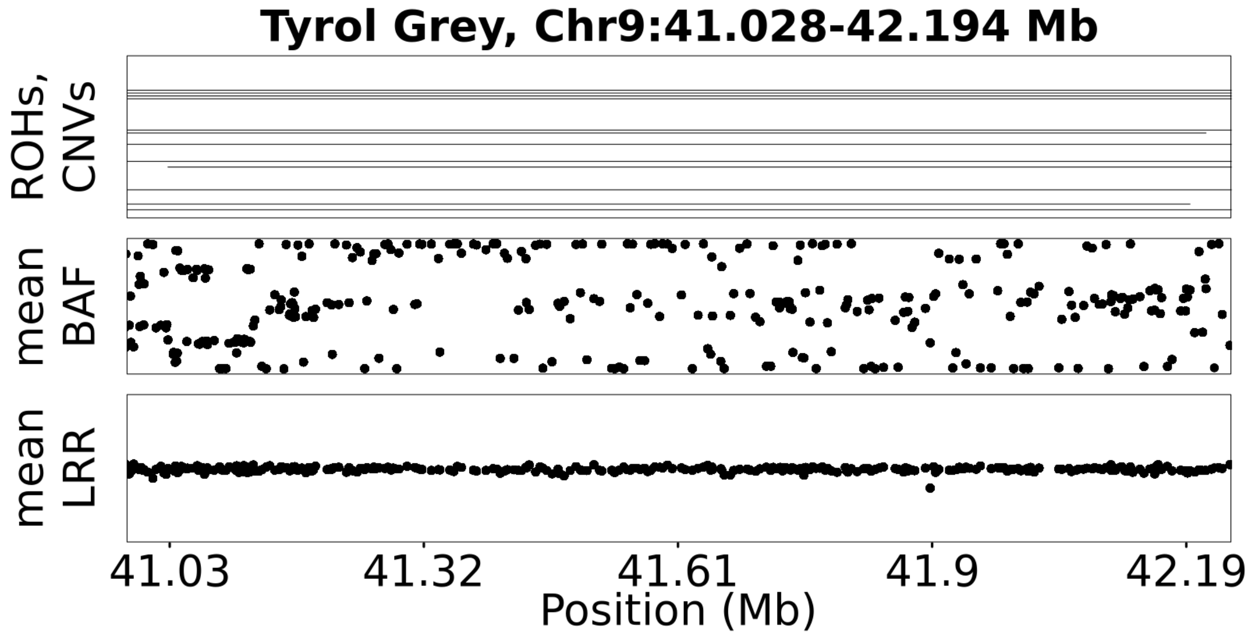
Tyrol Grey, Chr9:41.028-42.194 Mb
ROHs, CNVs
mean BAF
mean LRR
41.03
41.32
41.61
41.9
42.19
Position (Mb)

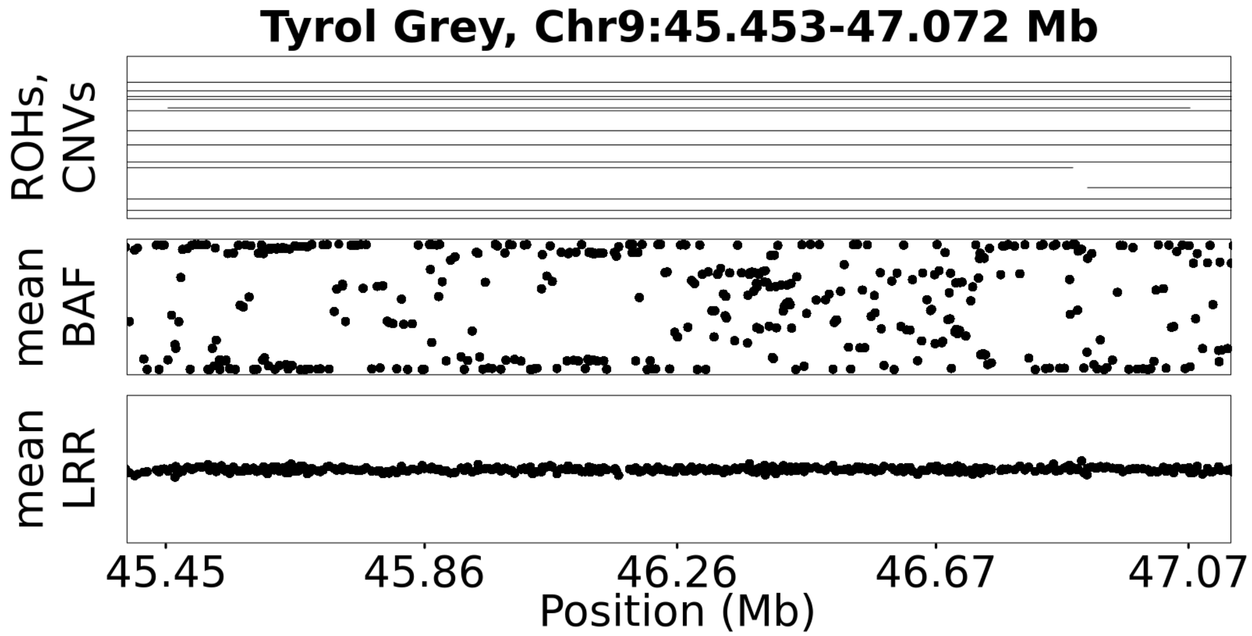
Tyrol Grey, Chr9:45.453-47.072 Mb
ROHs, CNVs
mean BAF
mean LRR
45.45
45.86
46.26
46.67
47.07
Position (Mb)

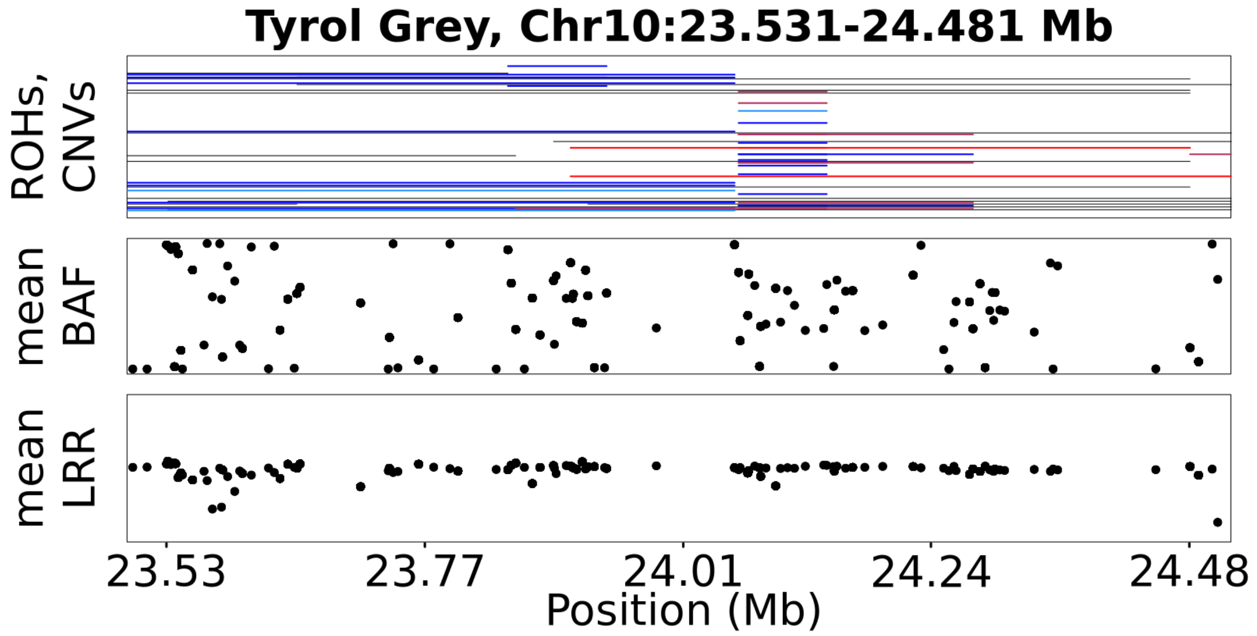
Tyrol Grey, Chr10:23.531-24.481 Mb
ROHs, CNVs
mean BAF
mean LRR
23.53
23.77
24.01
24.24
24.48
Position (Mb)

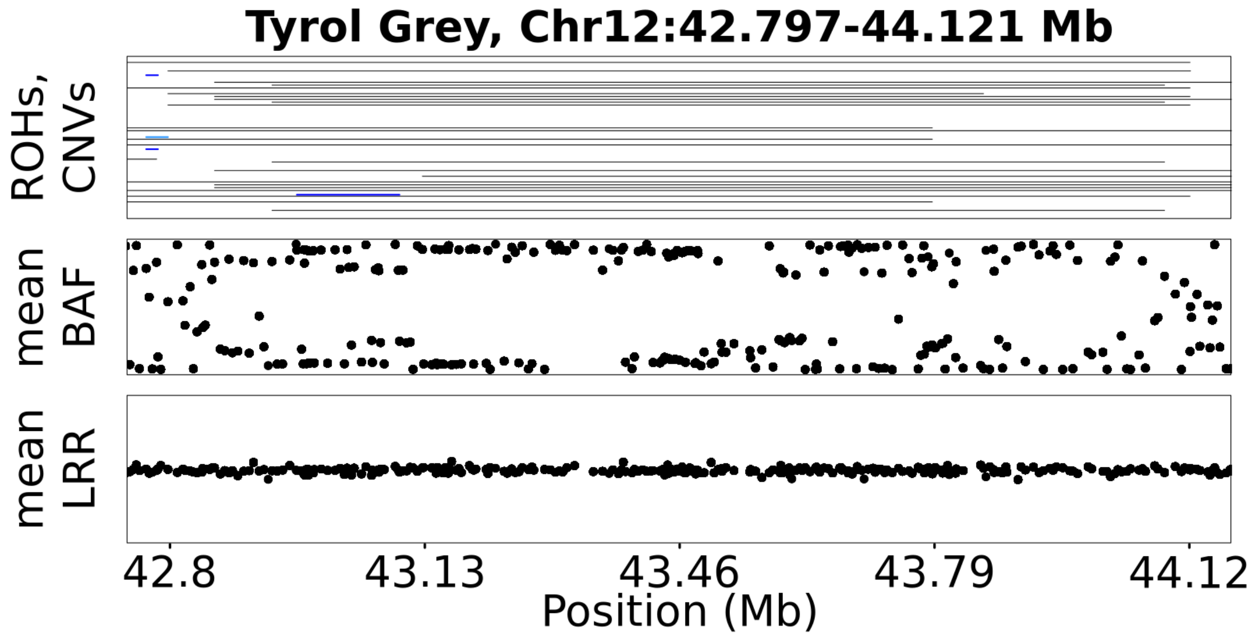
Tyrol Grey, Chr12:42.797-44.121 Mb
ROHs, CNVs
mean BAF
mean LRR
42.8
43.13
43.46
43.79
44.12
Position (Mb)

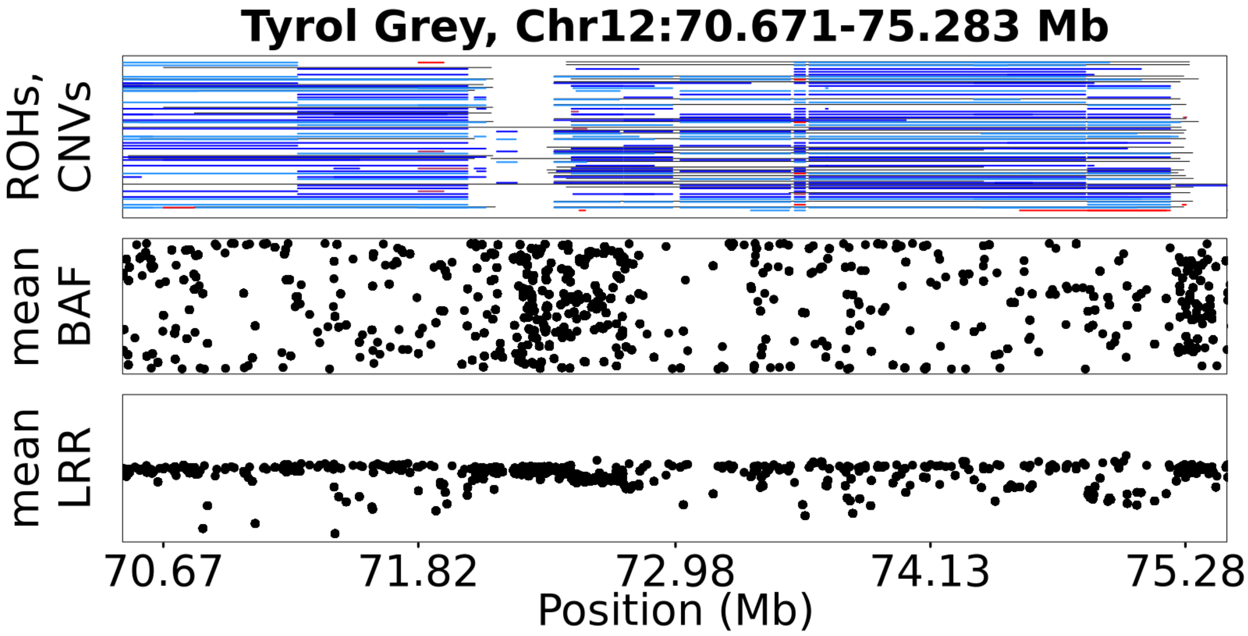
Tyrol Grey, Chr12:70.671-75.283 Mb
ROHs, CNVs
mean BAF
mean LRR
70.67
71.82
72.98
74.13
75.28
Position (Mb)

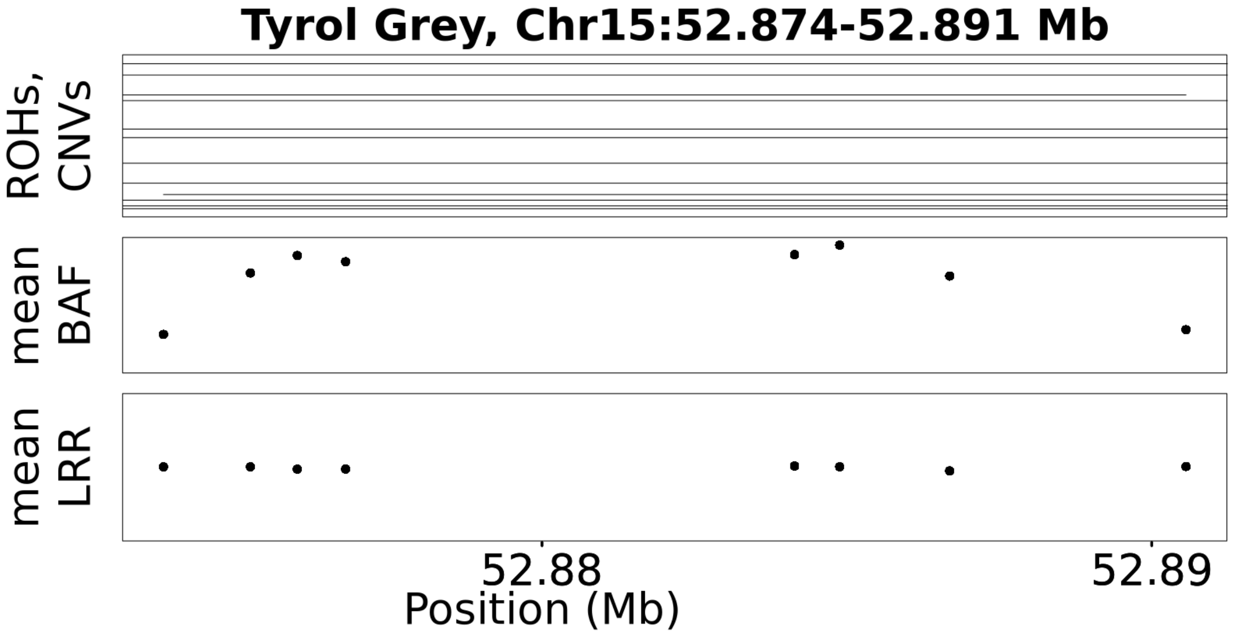
Tyrol Grey, Chr15:52.874-52.891 Mb
ROHs, CNVs
mean BAF
mean LRR
52.88
52.89
Position (Mb)

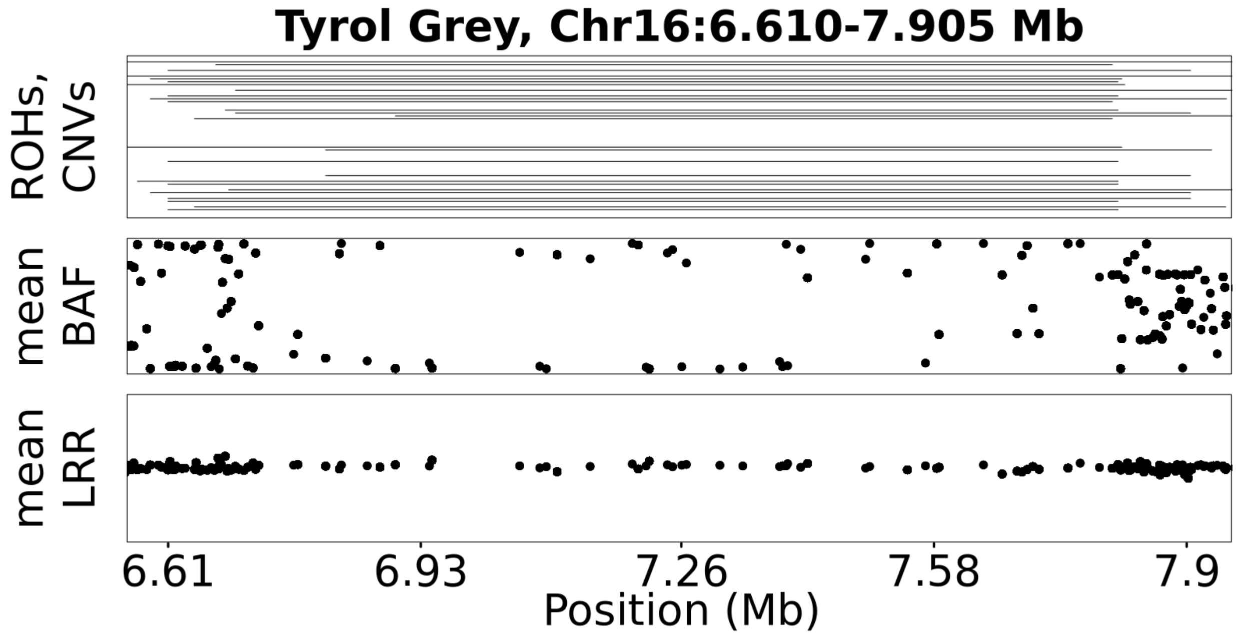
Tyrol Grey, Chr16:6.610-7.905 Mb
ROHs, CNVs
mean BAF
mean LRR
6.61
6.93
7.26
7.58
7.9
Position (Mb)

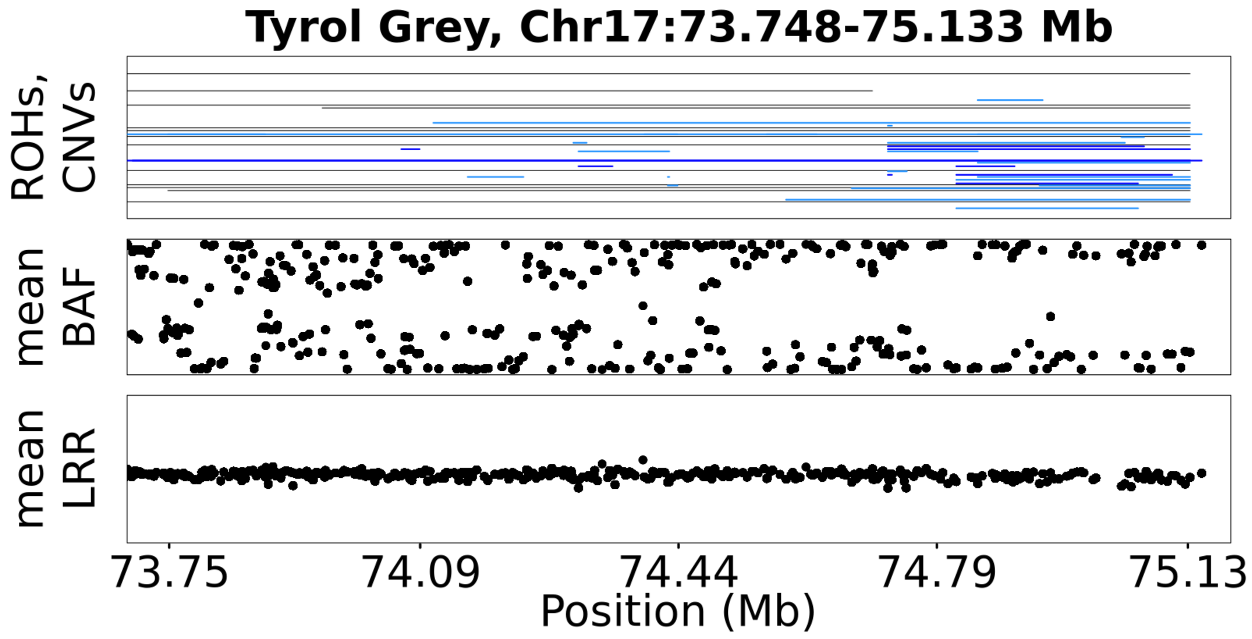
Tyrol Grey, Chr17:73.748-75.133 Mb
ROHs, CNVs
mean BAF
mean LRR
73.75
74.09
74.44
74.79
75.13
Position (Mb)

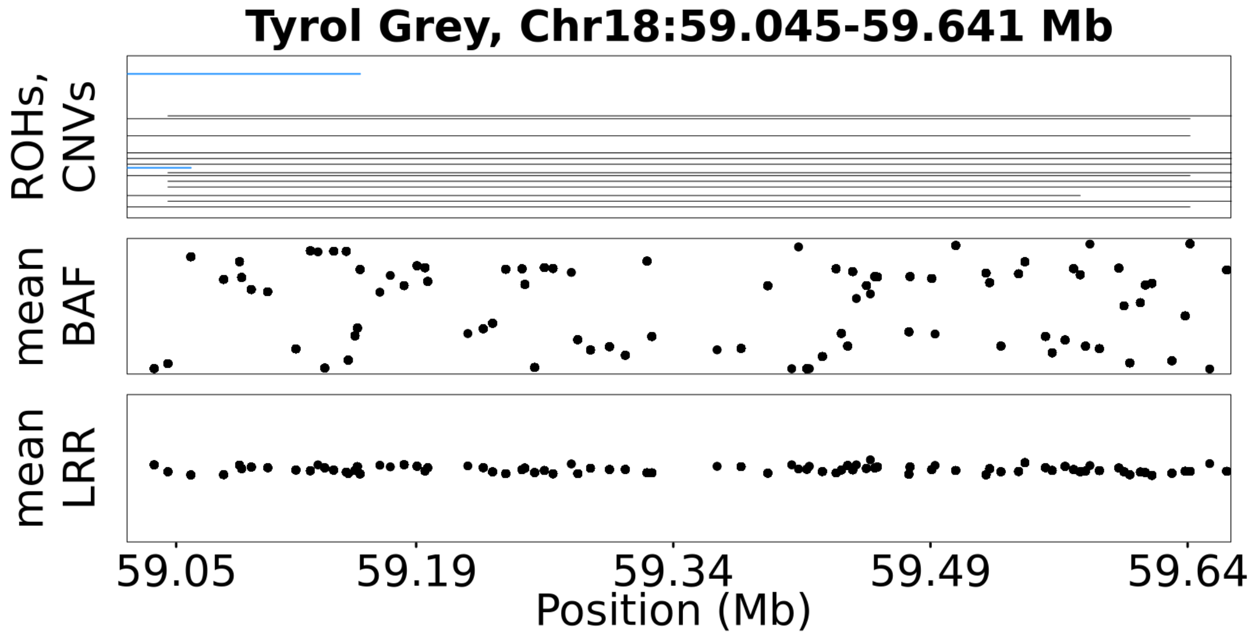
Tyrol Grey, Chr18:59.045-59.641 Mb
ROHs, CNVs
mean BAF
mean LRR
59.05
59.19
59.34
59.49
59.64
Position (Mb)

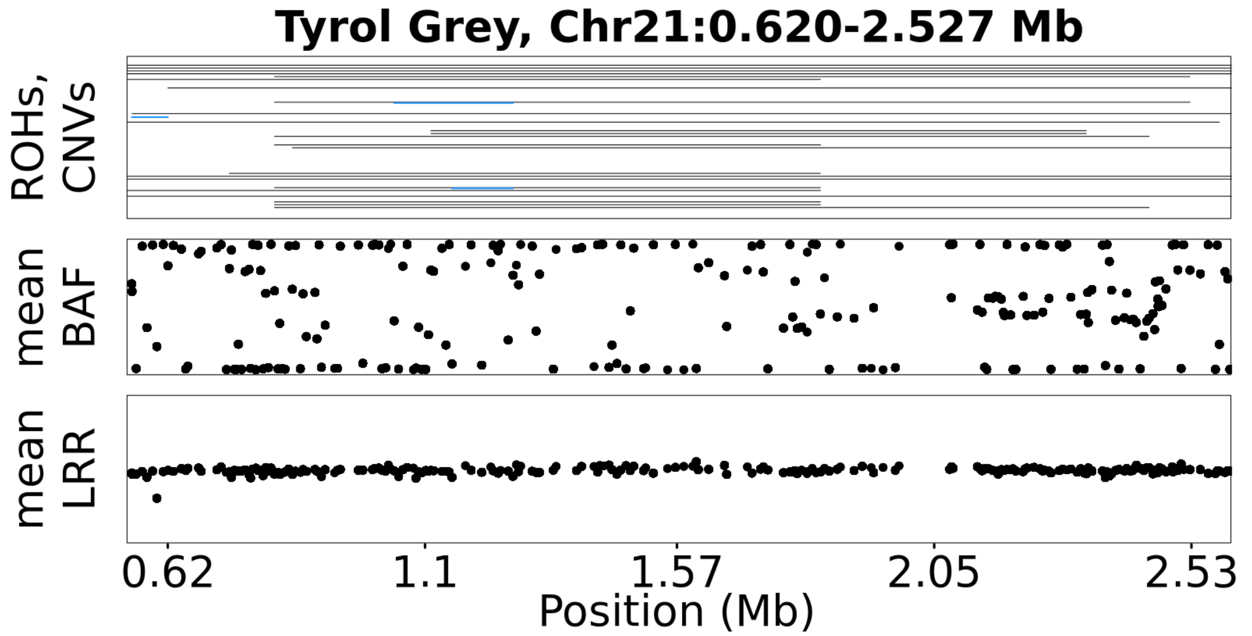
Tyrol Grey, Chr21:0.620-2.527 Mb
ROHs, CNVs
mean BAF
mean LRR
0.62
1.1
1.57
2.05
2.53
Position (Mb)

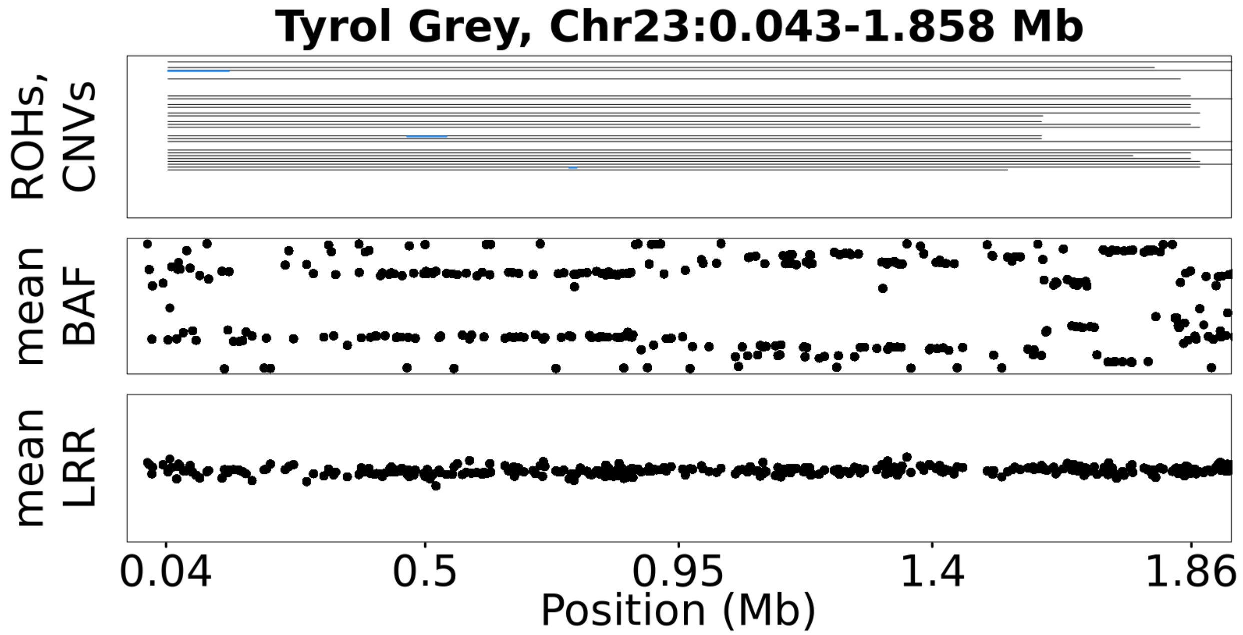
Tyrol Grey, Chr23:0.043-1.858 Mb
ROHs, CNVs
mean BAF
mean LRR
0.04
0.5
0.95
1.4
1.86
Position (Mb)

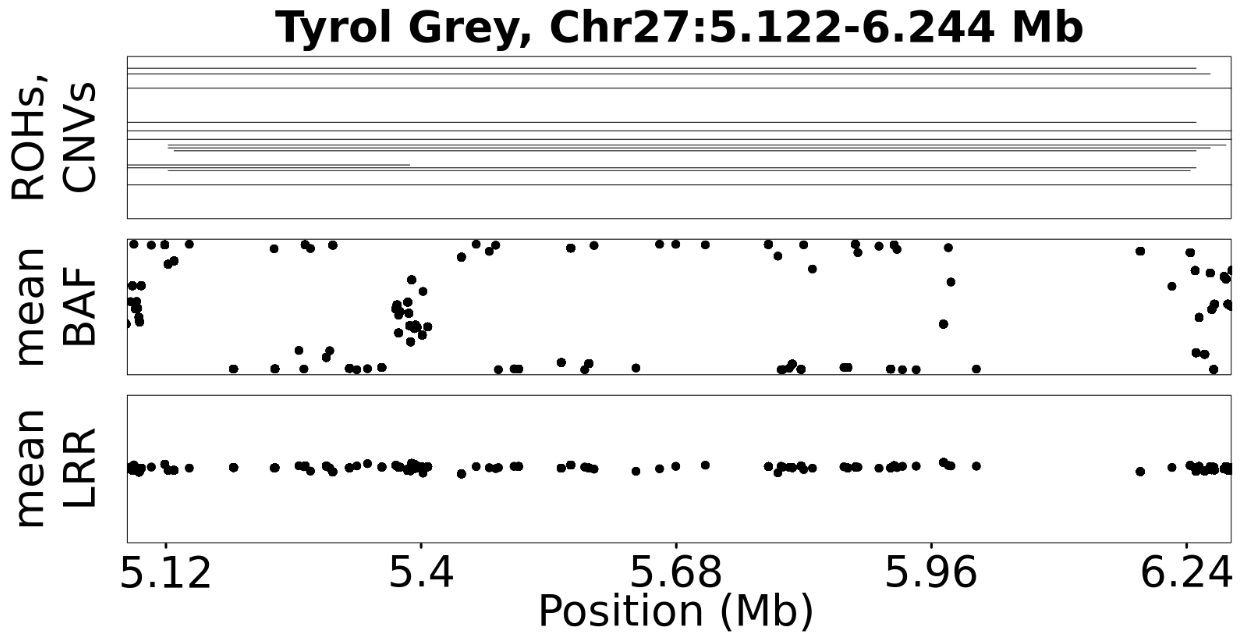
Tyrol Grey, Chr27:5.122-6.244 Mb
ROHs, CNVs
mean BAF
mean LRR
5.12
5.4
5.68
5.96
6.24
Position (Mb)

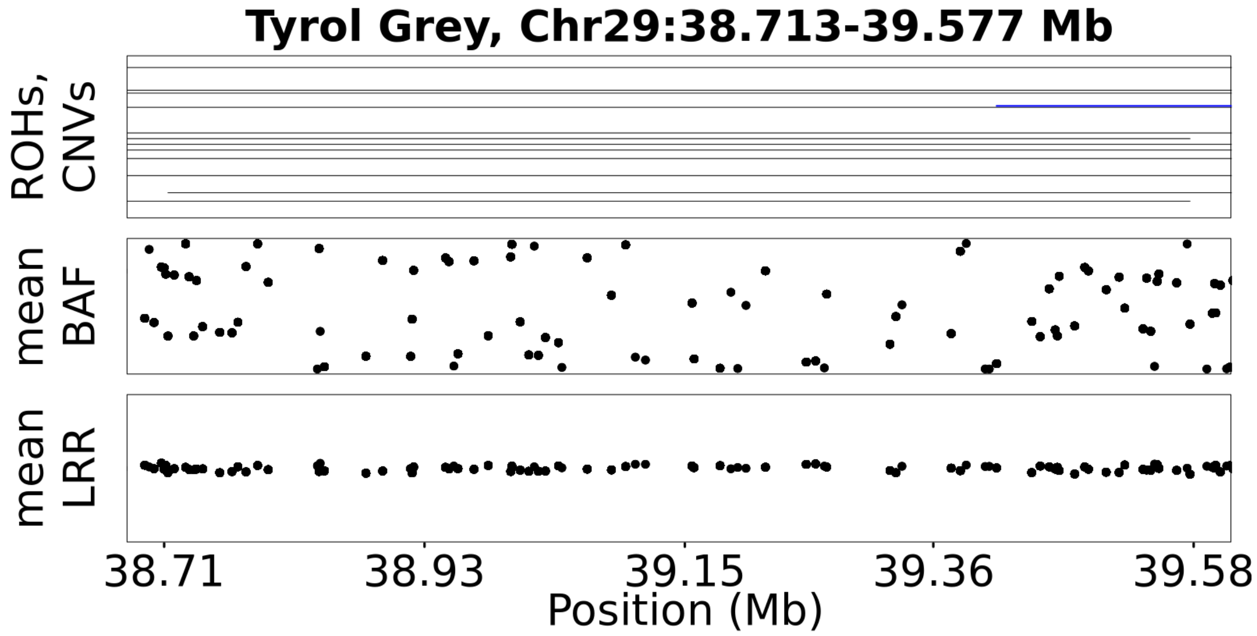
Tyrol Grey, Chr29:38.713-39.577 Mb
ROHs, CNVs
mean BAF
mean LRR
38.71
38.93
39.15
39.36
39.58
Position (Mb)
